# Photo-Triggerable Polymerization and Depolymerization of Stiff-Stilbene Lactones

**DOI:** 10.1021/jacs.5c10480

**Published:** 2025-08-21

**Authors:** Yong-Liang Su, Wei Xiong, Timothy M. Hunter, Kaitlyn S. Engle, Will R. Gutekunst

**Affiliations:** School of Chemistry and Biochemistry, 1372 Georgia Institute of Technology, Atlanta, Georgia 30332, United States

## Abstract

The development of photoswitchable polymers has unlocked new avenues for controlling polymerization and depolymerization through external stimuli. In this work, we introduce a strategy that integrates stiff-stilbene-based cyclic monomers with reversible *E*/*Z* photoisomerization to dynamically modulate the ring strain of monomers for ring-opening polymerization (ROP). A series of stiff-stilbene lactones with varying linker lengths were synthesized and systematically evaluated for their polymerization behavior. The resulting polymers exhibit tunable thermal and optical properties, with select derivatives displaying liquid crystalline behavior, and can be depolymerized to recover the original monomers. Density functional theory (DFT) calculations offer insights into the crucial role of *Z*/*E* photoisomerization in governing the polymerization and depolymerization process. This photoswitchable ROP system provides a new paradigm for designing sustainable polymers materials with adaptable and responsive properties.

## Graphical abstract

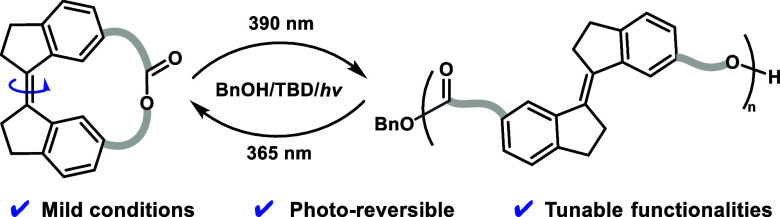

## Introduction

Ring-opening polymerization (ROP) has emerged as a cornerstone methodology for the synthesis of polymers with diverse structures and functionalities, driving advancements in fields such as advanced materials and sustainable technologies.^1^ Central to the success of ROP is the design of cyclic monomers, where enhanced ring strain is a critical factor in lowering the activation energy for ring opening and enabling thermodynamically favorable polymerization.^2^ Traditional strategies to induce ring strain rely on intrinsic structural features, such as small ring sizes or angular distortions.^3^ However, these conventional approaches often impose limitations on monomer diversity and polymerization conditions, restricting the scope of achievable materials.^4^

Chemically recyclable polymers synthesized through ROP have recently gained significant attention.^5^ Several polymer scaffolds, including cyclic (thio)­lactones,^6^ carbonates,^7^ thioethers,^8^ acetals,^9^ cycloalkenes,^10^ and others,^11^ have been developed. The balance between polymerization and depolymerization is fundamentally determined by thermodynamic factors, including enthalpic (Δ*H*_p_) and entropic (Δ*S*_p_) contributions, as defined by Δ*G*_p_ = Δ*H*_p_ – *T*Δ*S*_p_. By carefully adjusting reaction parameters such as temperature and concentration, these polymers can undergo controlled depolymerization, allowing for the recovery of pristine monomers.^12^ Despite these advancements, it remains highly desirable to develop monomer scaffolds that enable dynamic modulation of ring strain to facilitate both efficient ROP and reversible depolymerization for monomer recovery.

*E/Z* isomerization, a well-established tool in organic chemistry, offers a dynamic tool to modulate molecular conformations and electronic properties, thereby influencing reactivity in a variety of chemical transformations.^13^ Specifically, *E/Z* isomerization can significantly alter ring strain in cyclic structures, as demonstrated in applications such as the strain-promoted azide–alkyne cycloaddition reaction (SPAAC) in bioorthogonal chemistry,^14^ and energy storage (Figure 1a).^15^ However, the application of *E/Z* isomerization to polymerization processes remains relatively underexplored, with only limited examples for ring-opening metathesis polymerization (ROMP, Figure 1a).^16^ For instance, Wang and co-workers reported the ROMP of *trans*-cyclooctene, which was prepared from *cis*-cyclooctene under ultraviolet light (254 nm) in a flow chemistry setup.^16a^ Klausen and co-workers reported the ROMP of strained *trans*-silacycloheptene.^16b^ In both cases, the driving force for the polymerization arose from the high ring strain in the *E* isomer compared to the lower strain in the *Z* isomer, showcasing the potential of *E/Z* isomerization to enhance polymerization efficiency through ring strain modulation. Nevertheless, the process currently depends on independently prepared *E* isomers and relatively demanding conditions, which may pose challenges.

**Figure 1:**
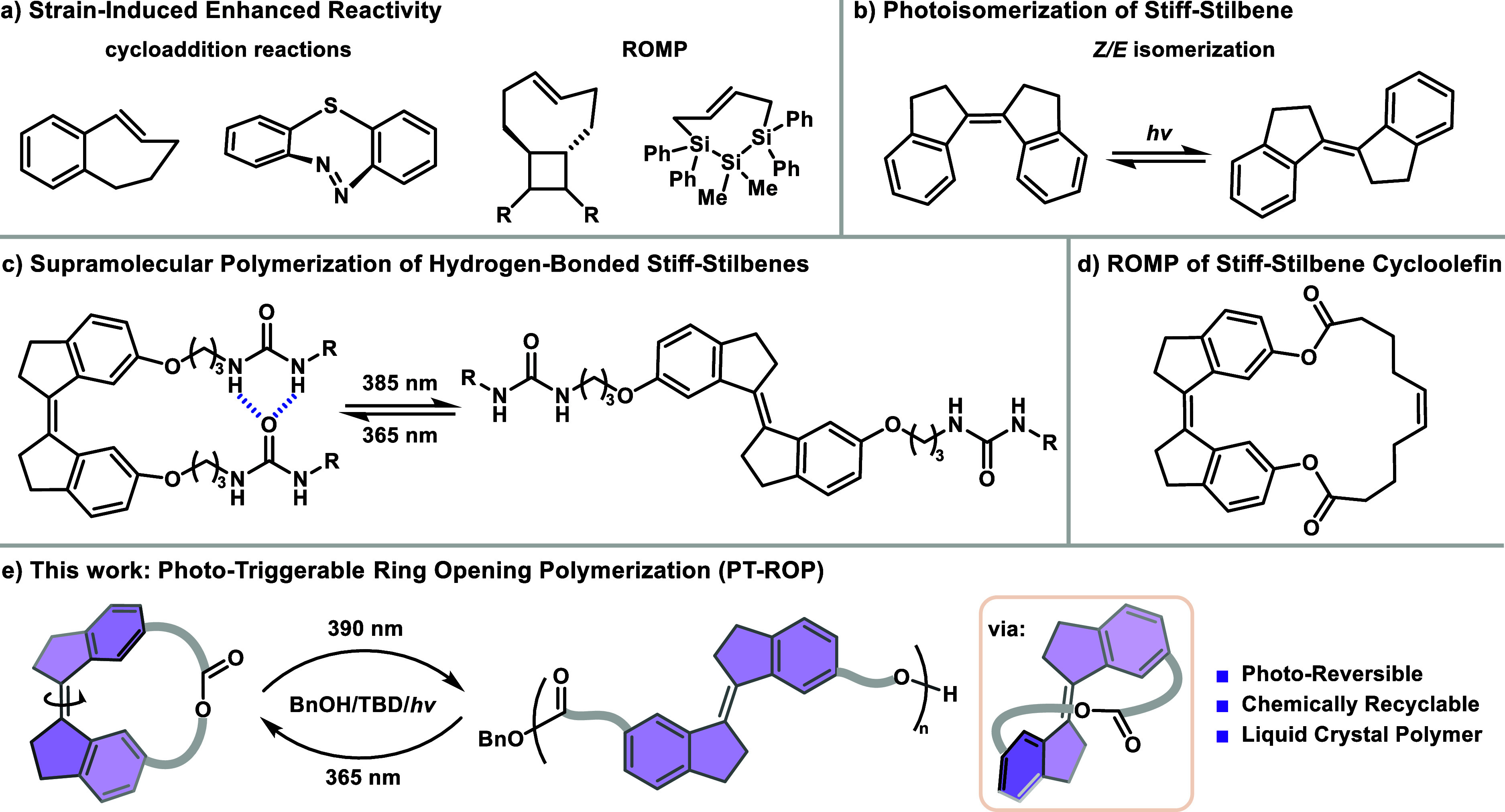
Concept of photoswitchable ring-opening polymerization. (a) Enhanced reactivity of the *E* isomer in cyclic scaffolds. (b) *Z/E* photoisomerization of stiff-stilbene. (c) Supramolecular polymers are assembled from stiff-stilbene monomers through noncovalent interactions. (d) ROMP of a stiff-stilbene cycloolefin by Cui. (e) Photoisomerization enabled synthesis of main-chain stiff-stilbene polymer.

Photoswitchable molecules, particularly those capable of reversible *E/Z* isomerization under different wavelengths of light, provide an attractive platform for integrating external stimuli into chemical processes.^17^ Among these, stiff-stilbene derivatives have garnered attention for their ability to undergo substantial geometric changes upon photoisomerization while also showing high stability.^18^ Specifically, stiff-stilbenes typically isomerize from *Z* to *E* when exposed to light at 300 nm and revert from *E* to *Z* at 360 nm illumination (Figure 1b).^19^ This reversible photoisomerization has made stiff stilbenes valuable building blocks in photodynamic triggers,^20^ molecular switches and machines,^21^ and switchable catalysts.^22^ For example, their ability to undergo dynamic supramolecular assembly, facilitated through hydrogen bonding,^23^ or metal–ligand coordination,^24^ offers promising avenues for constructing responsive and adaptive materials (Figure 1c). Cui and co-workers reported the ROMP of strained macrocycles containing stiff-stilbene units, although the resulting products were limited to oligomers, as characterized by MALDI- TOF (Figure 1d).^25^ Additionally, this system required independent isolation of the strained *E*-isomers prior to polymerization, possibly due to incompatibility of the ruthenium initiators at the wavelengths of light needed for olefin isomerization. Despite these promising developments, the integration of stiff-stilbene units into polymerization systems compatible with light to explore dynamic *Z/E* isomerization for designing photoswitchable monomers capable of reversable polymerization and depolymerization remains unexplored.^26^

Herein, we present a strategy for designing cyclic monomers that leverages the dynamic modulation of ring strain afforded by photoswitchable stiff-stilbene units. By incorporating this photoswitch into cyclic monomers, we enable light-driven control over ring strain, providing a unique pathway for achieving efficient and tunable ROP (Figure 1e). As proof of concept, we designed and synthesized stiff-stilbene-functionalized cyclic lactones with different linker lengths and investigated their polymerization behavior. The ring-opening process was further elaborated through DFT calculations, which support the enhanced ring strain through the *Z*/*E* isomerization. Furthermore, by switching the wavelength of light from 390 to 365 nm, the polymer was depolymerized back to the original monomer under diluted conditions, demonstrating a proof-of-concept for chemical recycling.

## Results and Discussion

### Monomer Design and Synthesis

The design of photoswitchable cyclic monomers was guided by the goal of integrating stiff-stilbene into a cyclic framework to study the effects of ring size and linker flexibility on isomerization dynamics and polymerizability. The esters were selected as linkers due to their synthetic accessibility, photostability, and ability to facilitate efficient ROP. Given the structural role of the linker cyclizing the stiff stilbene, it was hypothesized that its length would significantly influence the isomerization behavior of the corresponding monomer.^19b^ To systematically assess the effect of linker length on isomerization and subsequent polymerization, a series of cyclic ester monomers featuring a stiff-stilbene core and varying linker lengths were designed and synthesized (Figure 2). The monomers were prepared from readily available precursors, and the final cyclization was achieved via the McMurry reaction (Figure 2a and Supporting Information Scheme S1 for full synthetic protocols).^27^ The resulting monomers, **M1** ^**4**^–**M6** ^**11**^ (superscripted number refers to the number of atoms in the linker), were fully characterized with ^1^H NMR, ^13^C NMR and mass spectrometry. Notably, the structures of **M3** ^**8**^ and **M6** ^**11**^ were confirmed by single-crystal X-ray diffraction (SC-XRD, Figure 2d). The monomer **M6** ^**11**^ exhibited two distinct stable conformations in the *Z*-isomer crystals, highlighting the flexibility of the linker in larger sizes.

**Figure 2:**
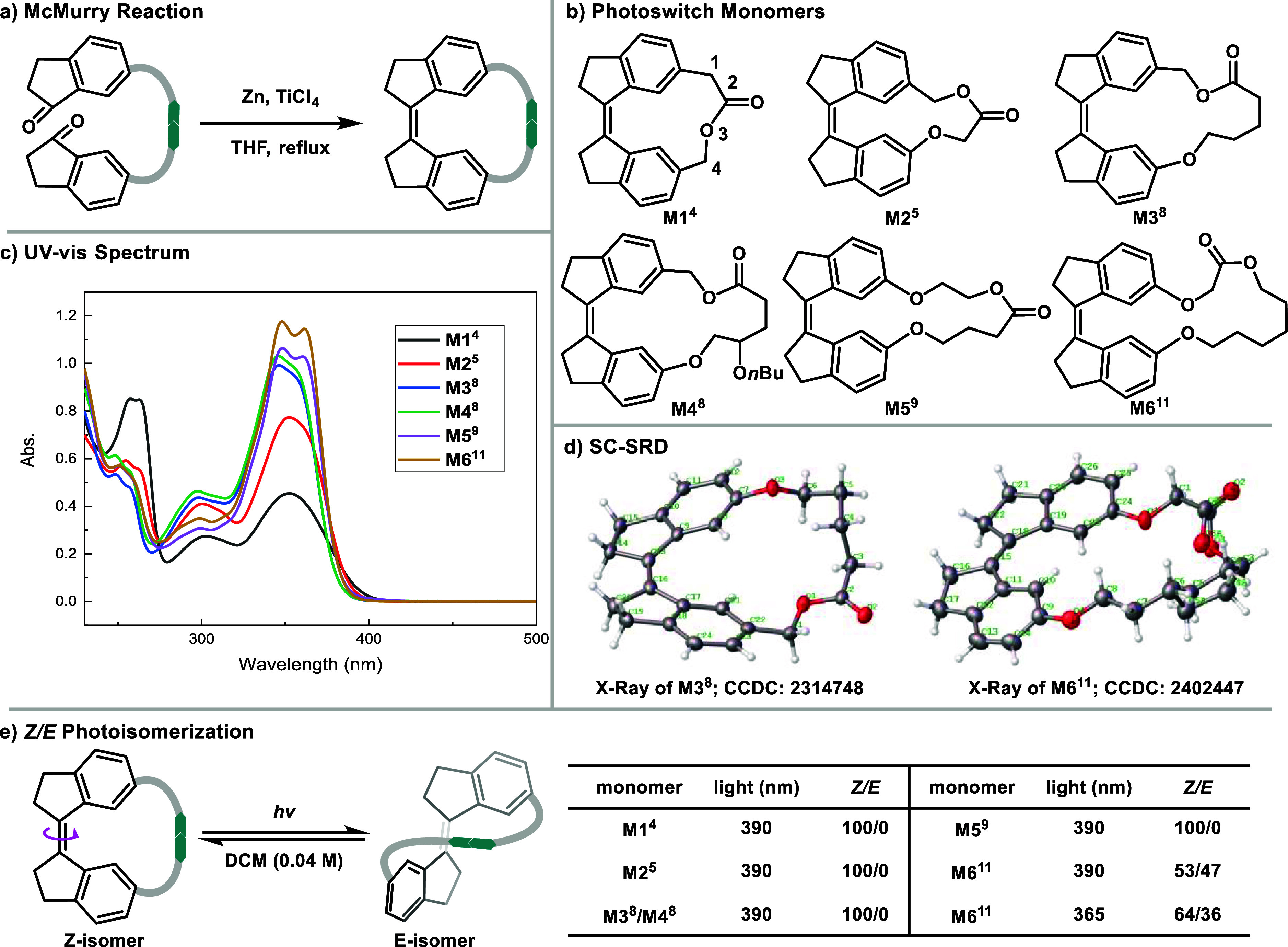
Design, synthesis, and characterization of photoswitch monomers. (a) McMurry reaction for cyclization. (b) Structures of monomers with varying linker lengths. Mi^j^ (i): monomer number; (j): linker length. (c) UV–vis spectra of the monomers. (d) The molecular structures of M3^8^ and M6^11^ as determined by SC-XRD. (e) *Z/E* photoisomerization of the monomers and their photostationary state ratios under different wavelengths of light.

UV–vis absorption spectroscopy was employed to investigate the photophysical properties of the monomers, revealing a clear dependence on linker length (Figure 2c). Subsequently, the isomerization behavior of these monomers was investigated using ^1^H NMR spectroscopy (Figure 2e–Supporting Information Figures S1–S7). Interestingly, no detectable isomerization was observed for monomers **M1** ^**4**^–**M5** ^**9**^, with linker lengths ranging from *L* = 4 to *L* = 9, likely due to two possible explanations: (i) the linkers in these monomers are too short to permit *E/Z* isomerization, or (ii) while isomerization is possible, the resulting isomers are energetically unfavorable and do not persist long enough to be observed by NMR. In the latter scenario, this would not exclude a photomediated polymerization if the strained *E*-isomer is sufficiently long-lived to react with an initiator or propagating chain-end. In contrast, monomer **M6** ^**11**^ exhibited observable *Z/E* isomerization, rapidly reaching a photostationary state. Additionally, the *Z/E* ratio could be effectively tuned by altering the irradiation wavelength, demonstrating the monomer’s photoswitchable behavior.

The polymerization of photoswitchable monomers (**M1** ^**4**^ **–M6** ^**11**^) was investigated under various conditions to evaluate their reactivity and potential for polymerization. These monomers exhibited distinct polymerization behavior depending on their molecular structures (Table 1). Monomers **M1** ^**4**^ and **M2** ^**5**^ displayed minimal reactivity, reaching only low conversions after 16 h of 390 nm irradiation, with no detectable polymer peak observed in the GPC trace (Table 1, entries 1 and 2). The ring–opening exchange reaction (single addition) with stoichiometric benzyl alcohol similarly exhibited low conversion across various catalysts commonly used in ester exchange reactions, including 1,5,7-triazabicyclo[4.4.0]­dec-5-ene (TBD), NHC-carbene 1,3-bis­(2,4,6-trimethylphenyl)-1,3-dihydro-2H-imidazole-2-ylidene (IMes), *p*-toluenesulfonic acid (PTSA) and phosphazene base P_4_-*t*Bu, further confirming the low reactivity (Supporting Information Table S1). In contrast, monomer **M3** ^**8**^ exhibited higher reactivity, achieving 85% conversion after optimization of solvents and concentration conditions (Table 1, entry 3 and Supporting Information Table S2). However, due to the limited solubility of both the monomer and the resulting polymer, the obtained product consisted of oligomers with approximately 10 repeating units. To enhance solubility, an *n*-butoxy group was introduced as a side chain to give monomer **M4** ^**8**^, which led to an increase in molecular weight and solubility throughout the reaction (Table 1, entry 4). Notably, the resulting polymer exhibited a predominant *E*-configuration with a detectable amount of the *Z*-isomer (*E*/*Z* = 4.6/1). Although increasing the loading of TBD relative to the initiator improved conversion, the molecular weight remained largely unchanged, with the highest *M*_n_ reaching 7.4 kDa (Table 1, entries 6 and 7). Notably, the polymerization of **M4** ^**8**^ in the absence of light resulted in less than 5% conversion, highlighting the crucial role of light in the polymerization process (Table 1, entry 5 and Supporting Information Table S4). For monomer **M5** ^**9**^, polymerization was conducted in toluene at 100 °C due to its poor solubility. Furthermore, the polymerization of **M6** ^**11**^ under optimized conditions led to molecular weights increasing as a function of the monomer-to-initiator ratio, ranging from 8.5 kDa to 12.5 kDa (Table 1, entries 9–13). The absence of light during polymerization significantly reduced conversion to 18%, further supporting the role of isomerization for productive propagation (Table 1, entry 14). The structure of **P6** was analyzed by matrix-assisted laser desorption/ionization-time-of-flight (MALDI-TOF) mass spectrometry (Figure 3b). The major group of molecular ion peaks were separated by 404.5 Da, consistent with the molar mass of the monomer **M6** ^**11**^. While linear polymers with benzyl alcohol derived chain ends were the predominant species, cyclic structures were also detected in the lower molecular weight region (<2000 Da). This is likely the origin of lower than anticipated molecular weights based on monomer-to-initiator ratios.

**Table 1:**
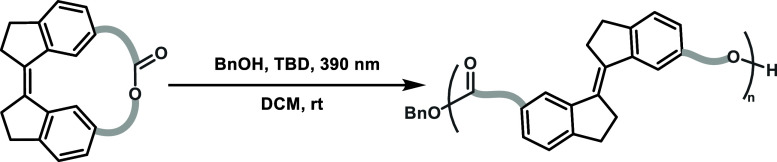
PT-ROP Results of Photoswitch Monomers

entry^a^	monomer	conc. (M)	[M]_0_:[I]_0_:[base]_0_	time (h)	conv. (%)^b^	*M*_n,SEC_ ^c^ (kDa)	Đ^c^
1	M1^4^	0.5	20:1:1	16	11^d^	^e^	
2	M2^5^	0.67	20:1:1	16	15^d^	^e^	
3	M3^8^	1	10:1:1	16	85	3.6	1.91
4	M4^8^	1.5	20:1:1	18	54^f^	6.2	2.09
5^g^	M4^8^	1.5	20:1:1	18	<5	^e^	
6	M4^8^	1.5	20:1:3	18	83	6.9	2.04
7	M4^8^	1.5	50:1:3	18	65	7.4	2.03
8^h^	M5^9^	0.5	10:1:1	6	73	3.4	2.99
9	M6^11^	1.0	20:1:1	16	>99^i^	8.5	2.36
10	M6^11^	1.0	30:1:1	16	91	10.7	2.04
11	M6^11^	1.0	40:1:1	16	85	11.0	2.18
12	M6^11^	1.0	50:1:1	16	76	12.5	2.04
13	M6^11^	1.0	60:1:1	16	68	12.4	1.93
14^g^	M6^11^	1.0	50:1:1	16	18^d^	^e^	

a Polymerizations were performed under N_2_ atmosphere with BnOH as the initiator.

b Monomer conversion, determined by ^1^H NMR spectroscopy in CDCl_3_.

c Determined by CHCl_3_ size-exclusion chromatography (SEC) calibrated using polystyrene standards.

d No polymer peak in GPC, only oligomers observed by ^1^H NMR.

e No polymer was detected by GPC.

f 47% isolated yield with a *E/Z* ratio of 4.6:1 in the polymer.

g Without light.

h Toluene, 100 °C.

i 86% isolated yield with exclusive *E*-isomer in the polymer. Conc.: initial monomer concentration.

**Figure 3:**
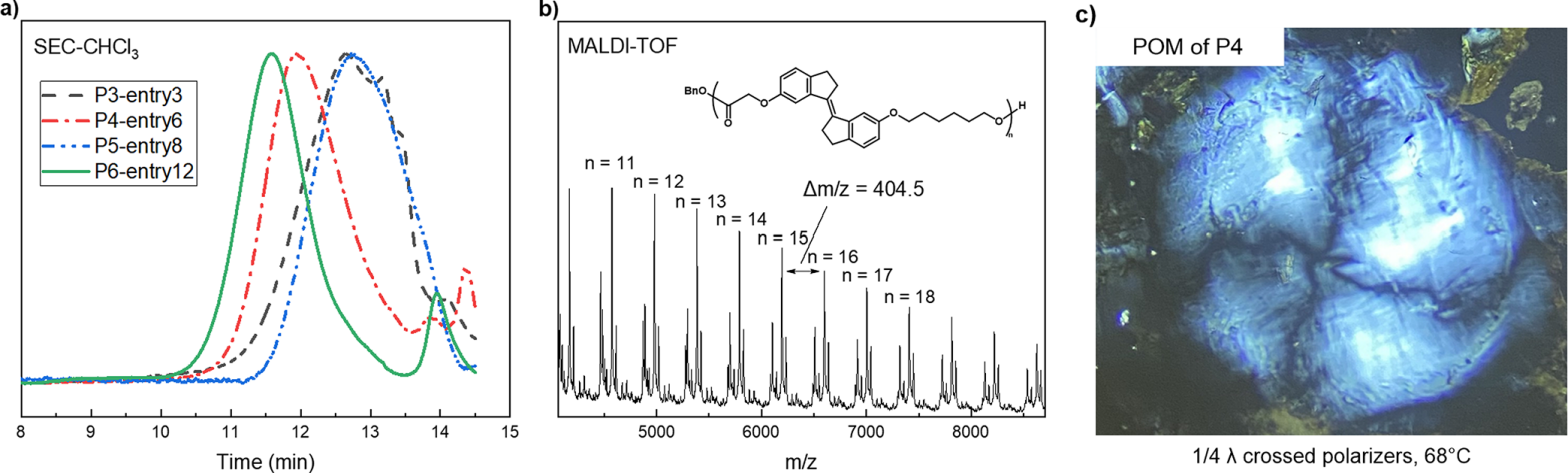
PT-ROP of photoswitch monomers and characterization of the polymers. (a) SEC curves for P3 to P6. (b) MALDI-TOF mass spectrum of P6 (Table 1, entry 13). (c) POM of P4 (Table 1, entry 7).

Differential scanning calorimetry (DSC) provided insight into the thermal properties of the polymers, revealing a glass transition temperature (*T*_g_) of 44 °C for **P4** and 63 °C for **P6** (Supporting Information Figure S19). Due to the presence of a rigid rod-like indene unit connected by flexible alkyl chain linkers, we hypothesized that this polymer might exhibit liquid crystalline behavior. While DSC traces show only a glass transition at 44 °C, the absence of a distinct liquid crystal transition peak is not uncommon among main-chain liquid crystalline polymers with high dispersity.^28^ The polarized optical microscopy (POM, Figure 3c) analysis of **P4** revealed birefringence and flow behavior above its *T*_g_, with a schlieren-like texture observed at 68 °C upon annealing. These observations indicate the formation of ordered domains, suggesting that the polymer exhibits a certain level of structural organization above its glass transition temperature.

To expand the structural and functional diversity of the polymer system, monomer **M4** ^**8**^ was copolymerized with ε-caprolactone (CL) under varying feed ratios (Table 2). The incorporation of **M4** ^**8**^ was systematically controlled by adjusting the initial monomer feed ratio ([**M4** ^**8**^]_0_­[CL]_0_­[BnOH]_0_), allowing tuning of copolymer composition and properties. Higher CL content resulted in increased molecular weight, with *M*_n_ ranging from 8.2 kDa (20:20:1) to 27.1 kDa (20:200:1). DSC analysis revealed a progressive decrease in *T*_g_ from 5 °C to −44 °C with increasing CL incorporation, while the melting temperature increased from 38 to 53 °C, indicating enhanced crystallinity ­(Figure 4b). TGA measurements further demonstrated improved thermal stability, with the 5% weight loss temperature (*T*_d,5%_) increasing from 331 to 372 °C as the CL fraction increased. DOSY NMR spectra (Figure 4c) confirmed the formation of well-integrated copolymers, as evidenced by a single diffusion coefficient, suggesting a homogeneous microstructure.

**Table 2:**
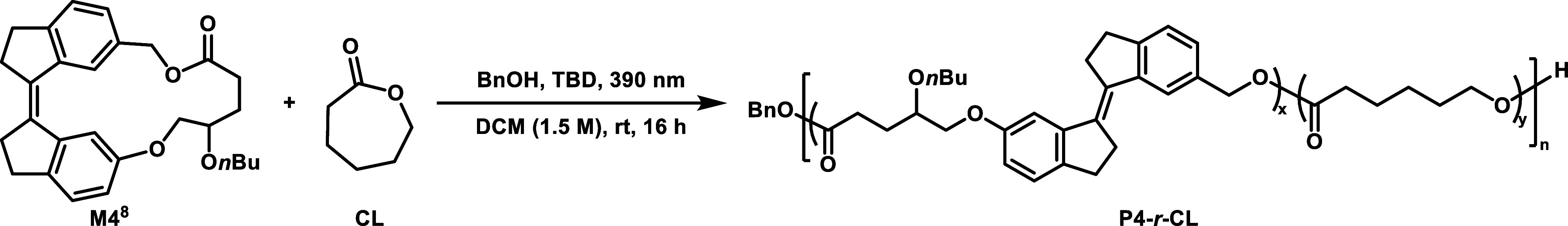
Copolymerization of Photoswitch Monomer M4^8^ with Caprolactone (CL)

entry^a^	[M4^8^]_0_:[CL]_0_:[I]_0_	conv. (M4^8^, %)^b^	conv. (CL %)^b^	M4^8^ (%)^b^	*M*_n,SEC_ (kDa)^c^	Đ^c^	*T*_d,5%_ (°C)^d^	*T*_g_ (°C)^e^	*T*_m_ (°C)^e^
1	20:20:1	87	99	41.6^f^	8.2	1.98	331	5	^g^
2^h^	20:20:1	30	99	23.6	4.7	1.58		–27	^g^
3	20:50:1	86	99	24.2	10.5	2.01	357	–20	^g^
4	20:100:1	70	99	11.0	17.2	1.84	370	–44	38
5	20:150:1	62	99	6.9	21.4	2.05	372	^g^	49
6	20:200:1	60	99	5.1	27.1	2.09	372	^g^	53

a Copolymerizations were performed under N_2_ atmosphere with BnOH as the initiator.

b Monomer conversion, and M4^8^ incorporation of the copolymer were determined by ^1^H NMR spectroscopy in CDCl_3_.

c Determined by CHCl_3_ size-exclusion chromatography (SEC) calibrated using polystyrene standards.

d Temperature causing a 5% weight loss, measured by thermogravimetric analysis (TGA).

e Glass transition temperature and melting temperature, measured by differential scanning calorimetry (DSC).

f 73% isolated yield with a *E/Z* ratio of 4.1:1 in the copolymer.

g The *T*_g_ or *T*_m_ could not be detected by DSC.

h without light.

**Figure 4:**
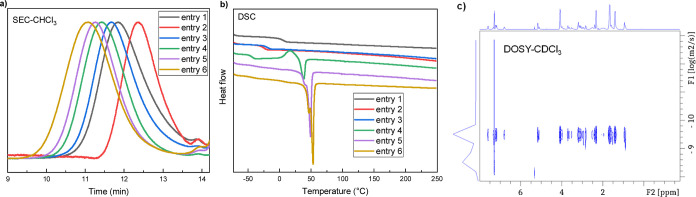
Copolymerization of photoswitch monomer M4^8^ with caprolactone (CL). (a) SEC curves for copolymers produced at different [M4^8^]_0_[CL]_0_[BnOH]_0_ ratios. (b) DSC curves of the produced copolymers. (c) DOSY NMR spectrum of copolymer (Table 2, entry 3).

### Proposed Mechanism

The experimental results reveal distinct ROP behavior among monomers with varying linker length, highlighting the significant influences of ring size on reactivity. To better understand the effect of ring size, the *Z/E* isomerization of the stiff-stilbene moiety was first examined computationally for select monomers. Geometry optimizations of the *Z*- and *E*-configurations of **M1** ^**4**^, **M3** ^**8**^ and **M6** ^**11**^ were performed, with an acyclic stiff-stilbene serving as a reference (Figure 5a–d). Given that isomerization involves significant changes in the dihedral angle (θ), this parameter was used as a descriptor of the isomerization. As shown in Figure 5, the optimized dihedral angles of the stiff-stilbene moiety for free stiff-stilbene, **M1** ^**4**^, **M3** ^**8**^ and **M6** ^**11**^ in their *Z*-configurations are 9.6°, 1.0°, 10.8°, and 12.3°, respectively. For the *E*-configurations, free stiff-stilbene adopts a nearly planar structure with a dihedral angle of 179°, while the angle decreases to 132° in **M3** ^**8**^ and 155° in **M6** ^**11**^, reflecting the increasing structural constraint imposed by the tethered ring. Notably, an energy minimum ***E***-**M1** ^**4**^ could not be located, likely due to the extreme angular strain imposed, making this configuration thermodynamically inaccessible.

**Figure 5:**
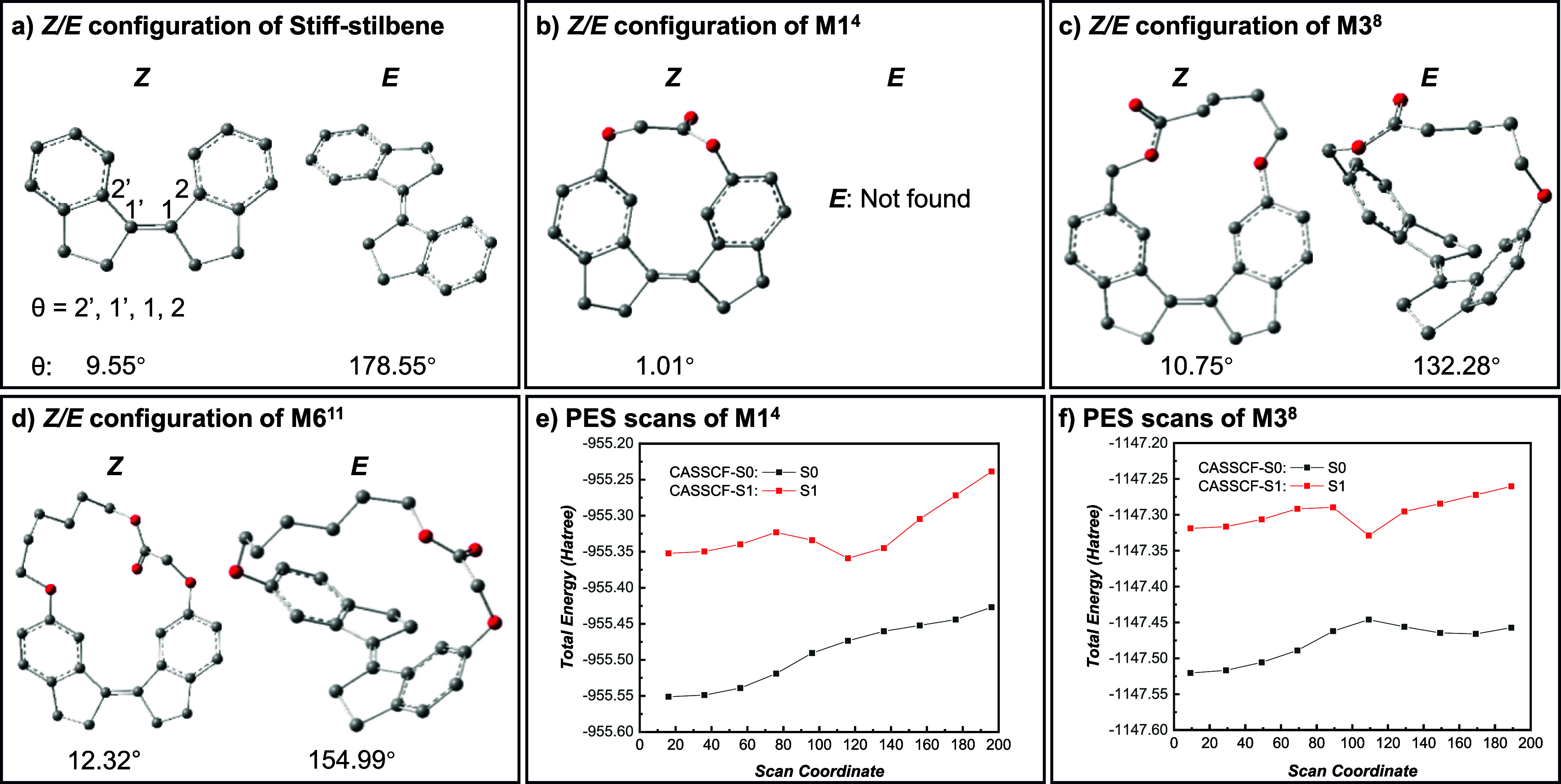
Optimized *Z/E* geometries of (a) an acyclic stiff-stilbene, (b) M1^4^, (c) M3^8^, (d) M6^11^, and potential energy surface (PES) scans of (e) S0 and S1 states of M1^4^ and (f) S0 and S1 states of M3^8^.

To gain deeper insight into the photoinduced *Z/E* isomerization process, potential energy surface (PES) scans of the monomer **M3** ^**8**^ and **M1** ^**4**^ were performed using constrained geometry optimization with systematic variation of the dihedral angle. This approach provides a practical and efficient method for capturing key features of both the ground state (*S*_0_) and first excited state (*S*_1_) potential energy landscapes during the isomerization process. The PES scan was conducted at the complete active space self-consistent field (CASSCF) level of theory, a method that has been validated as an effective tool for studying the *Z/E* isomerization of stilbene derivatives.^29^ As shown in Figure 5f, the *S*_0_ PES of **M3** ^**8**^ exhibits a energy profile for double-bond rotation: the energy initially increases as the dihedral angle deviates from planarity, reaching a maximum near ∼110°, before decreasing again. In contrast, the *S*_1_ PES follows an inverse trend, with the energy reaching a minimum at ∼110°. The minimum *S*_0_/*S*_1_ energy gap at ∼110° suggests the presence of a conical intersection (CI) between the *S*_0_ and *S*_1_ PES near this geometry, a hallmark of *Z*–*E* photoisomerization in stilbene derivatives, enabling rapid isomerization through a nonradiative decay pathway. In contrast, the *S*_0_ PES of **M1** ^**4**^ exhibits a fundamentally different profile, lacking a distinct energy maximum throughout the dihedral scan in the *S*_0_ state (Figure 5e). This can be attributed to substantial accumulation of ring strain, which counteracts the energy reduction associated with the formation of a conjugated π-system. This suggests that the *E*-configuration of **M1** ^**4**^ is thermodynamically inaccessible. Additionally, a critical difference between **M3** ^**8**^ and **M1** ^**4**^ may be the absence of a conical intersection between the *S*_0_ and *S*_1_ PES in **M1** ^**4**^ which prevents *Z/E* photoisomerization altogether. This result is consistent with experimental observations, where **M1** ^**4**^ does not undergo ROP under photoirradiation, further supporting the hypothesis that *Z/E* isomerization is critical for the photoinduced polymerization process.

The increasing ring strain energy associated with isomerization is reflected in the Gibbs free energy differences (Δ*G*) between *Z* and *E* isomers: 28.9 kcal/mol for **M3** ^**8**^ and 12.8 kcal/mol for **M6** ^**11**^ ­(Figure 6). Additionally, the transition states for the *Z*/*E* isomerization of **M3** ^**8**^ and **M6** ^**11**^ were identified, with energy barriers of ∼39.4 kcal/mol and ∼36.4 kcal/mol, respectively (Figure 6). Notably, the reverse *E*-to-*Z* isomerization differs between the two monomers: **M3** ^**8**^ exhibits a lower energy barrier of ∼10.5 kcal/mol, allowing for thermal retro-isomerization, whereas **M6** ^**11**^ has a higher barrier of ∼23.6 kcal/mol that leads to a kinetically trapped *E*-isomer after photoisomerization. This is consistent with experimental findings in which *E* **-M6** ^**11**^ can be observed via ^1^H NMR spectroscopy (Supporting Information Figures S6 and S7) at room temperature following photoirradiation.

**Figure 6:**
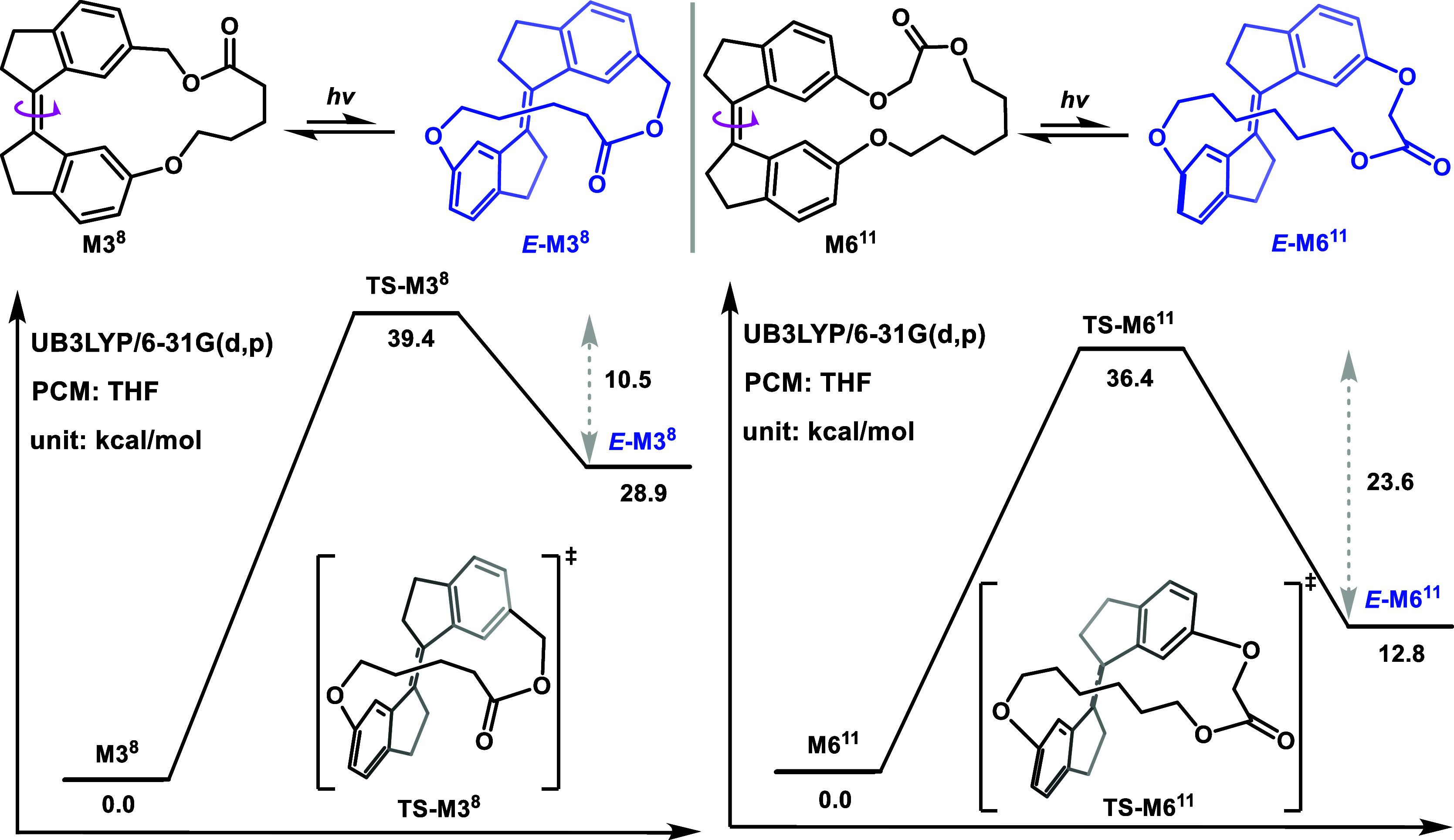
Free energy profile of *Z/E* isomerization of M3^8^ and M6^11^.

To further elucidate the detailed reaction pathway of ring-opening polymerization (ROP), density functional theory (DFT) calculations were performed on all relevant intermediates (**INT**) and transition states (**TS**) for monomer **M3** ^**8**^ following previous studies by Goodman (Figure 7).^30^ To simplify the computational model, methanol was chosen as the initiator, and TBD was employed as the catalyst. The ring-opening process consists of two primary steps: nucleophilic addition and subsequent ring-opening, with TBD acting as a proton shuttle in both steps. First, the energy profile of the ring-opening process for the *Z* **-M3** ^**8**^ was investigated. The calculated Gibbs free energy difference (Δ*G*) for the entire ROP process is approximately 3.8 kcal/mol (**INT4** to *Z* **-M3** ^**8**^), aligning well with the experimentally observed low conversion (<5%) of the *Z*-form in the absence of light. In contrast, the ROP of *E* **-M3** ^**8**^, shows a much lower Δ*G* of −24.0 kcal/mol (**INT2** to *E* **-M3** ^**8**^), indicating a thermodynamically favorable polymerization pathway. These results suggest that, even though *Z/E* isomerization is not directly observed in **M3** ^**8**^ to **M5** ^**9**^, it still plays a critical role in determining the efficiency of the ROP process.

**Figure 7:**
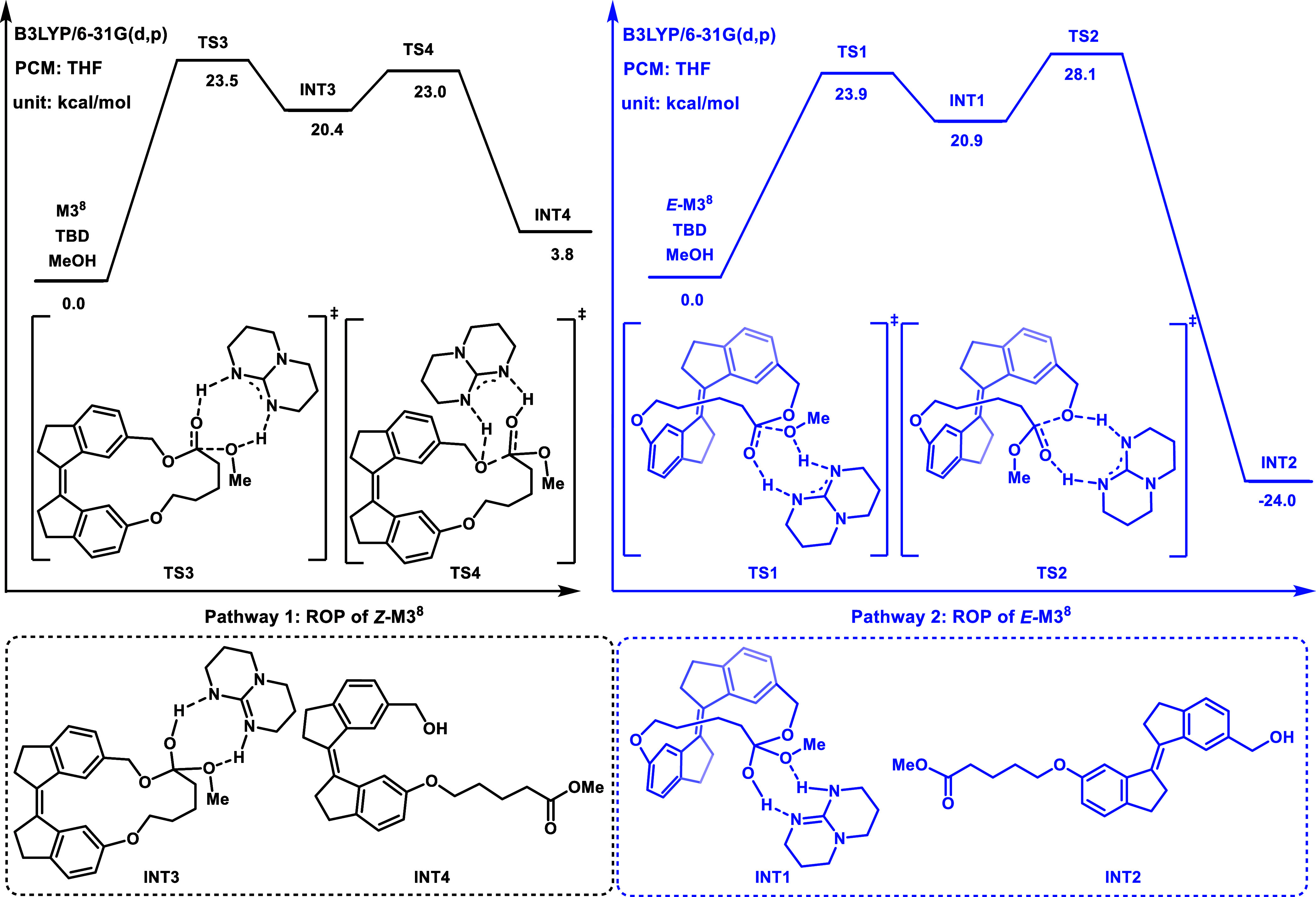
Free energy profile for the initiation step of ROP of *Z/E*-M3^8^.

### Chemical Recyclability

As a model reaction to evaluate the recyclability of the resulting polymers, the ring–opening exchange product of **M4** ^**8**^ with benzyl alcohol, **ROEM4** ^**8**^, was subjected to *Z/E* photoisomerization under controlled conditions. Upon irradiation at 390 nm, the equilibrium exclusively favored the *E*-isomer (*Z/E*: 0/100), whereas exposure to 365 nm light resulted in a partial shift to the *Z*-isomer (*Z/E*: 10/90), as shown in Figure 8a. This reversible photoisomerization suggests that the polymer structure can be dynamically modulated through selective wavelength exposure, potentially leading to a pathway for ring-closing depolymerization. The UV–vis absorption spectra of *E* **-ROEM4** ^**8**^, *Z* **-ROEM4** ^**8**^, and **P4** further confirmed the distinct electronic environments associated with different configurations. While *E* **-ROEM4** ^**8**^ exhibited similar absorption profile with **P4**, the *Z* **-ROEM4** ^**8**^ showed a characteristic hypochromic shift permitting absorbance at longer wavelengths of light (Figure 8c). To evaluate the potential for wavelength-selective depolymerization, 365 nm irradiation was employed in the presence of TBD at a concentration of 10 mg/mL in DCM. Gratifyingly, this led to the successful recovery of monomer **M4** ^**8**^ in a yield of 46% (Figure 8b,d). Importantly, no formation of **M4** ^**8**^ was observed when 390 nm light was used under identical conditions, and only 21% conversion was detected in the dark, highlighting the key role of photoisomerization in facilitating depolymerization. Additional control experiments showed that under 365 nm irradiation, both *Z*- and *E*-isomers of **M6** ^**11**^ were generated from depolymerization of **P6** (31% conversion), while negligible depolymerization was observed in the dark (Supporting Information Table S11). While chain transfer may occur during the depolymerization process, these results support the need for a terminal unit in the Z-configuration to facilitate cyclization back to the original monomer. Collectively, these results highlight the reversible nature of the polymerization process, though with moderate efficiency, and establish a proof-of-concept for photoswitchable polymers for recyclable materials applications.

**Figure 8:**
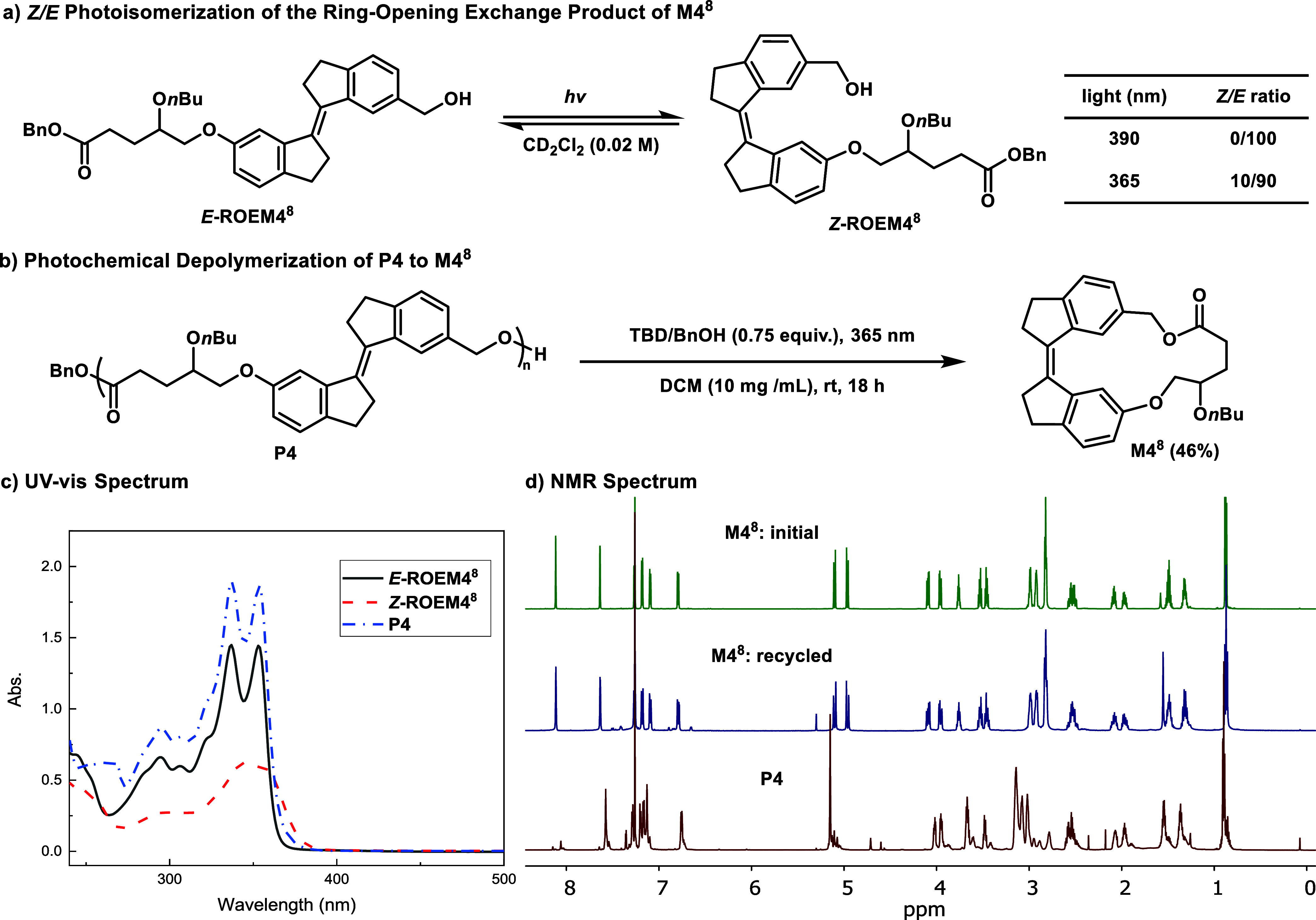
Photoisomerization-driven structural modulation and depolymerization of P4. (a) Photoisomerization between *E*-ROEM4^8^, and *Z*-ROEM4^8^. (b) Depolymerization of P4. (c) UV–vis spectra of *E*-ROEM4^8^, *Z*-ROEM4^8^, and P4. (d) Overlays of ^1^H NMR spectra.

## Conclusions

In this study, we have demonstrated a photoswitchable polymerization strategy by incorporating stiff-stilbene-functionalized cyclic monomers into a ROP framework, enabling light-triggered modulation of polymerization and depolymerization pathways. The designed monomers exhibited *Z*/*E* isomerization-dependent polymerization behavior, where the *E*-configuration in the monomer facilitated efficient polymerization through enhanced ring strain, and the *Z*-configuration in the polymer promoted depolymerization, enabling chemical recyclability. Moreover, DFT calculations provided insight into the isomerization-induced ring strain variations, supporting the proposed mechanism. The resulting polymers exhibited tunable thermal properties, with certain derivatives displaying liquid crystalline behavior, broadening their potential applications in responsive and adaptive materials. This approach opens new avenues for designing stimuli-responsive polymers with applications in sustainable and functional materials.

## Supplementary Material



## Notes

Experimental procedure and spectroscopic data for all new compounds (PDF), and X-ray crystallographic data for **M3** ^**8**^ and **M6** ^**11**^ (PDF)
