# Supplementary material for: Photo-Triggerable Polymerization and Depolymerization of Stiff-Stilbene Lactones
Source: J Am Chem Soc. 2025 Aug 21;147(35):32054–63. doi: 10.1021/jacs.5c10480 (PMC12412160; doi:10.1021/jacs.5c10480)
Supplement: Supplementary file 1 [file ja5c10480_si_001.pdf]

Supporting Information

Photo-Triggerable Polymerization and Depolymerization of Stiff-Stilbene Lactones

Yong-Liang Su[1], Wei Xiong[1], Timothy M. Hunter[1], Kaitlyn S. Engle[1], and Will R. Gutekunst[1,*]

[1]*School of Chemistry and Biochemistry, Georgia Institute of Technology, 901 Atlantic Drive NW, Atlanta, Georgia 30332, United States.*

[*]Correspondence to: willgute@gatech.edu

Table of Contents

1. General information S2
2. Synthesis of photoswitch monomers S3
3. Photoisomerization of monomers M1[4]-M6[11]. S9
4. Optimization of the polymerization conditions S13
5. General polymerization procedure and characterization of polymers S16
6. Characterizing polymer chain-ends using MALDI-TOF S23
7. TGA and DSC studies of (co)polymers S24
8. Chemical recycling of phoswitchable polymers S25
9. Polarized optical microscopy (POM) of P4 and P6 .... S29
10. X-ray crystal structure of the monomer M3[8] and M6[11] S30
11. Computational details S34
12. NMR spectrum of products S64
13. References S84

1. General information

General methods. All reactions were carried out under a nitrogen atmosphere with dry solvents using anhydrous conditions unless otherwise stated. Dry, degassed dichloromethane (DCM), *N*,*N*-dimethylformamide (DMF), toluene, and tetrahydrofuran (THF) were obtained from a JC Meyer solvent purification system. Diphenyl ether (Ph_2O), o-xylene, trifluorotoluene ($PhCF_3$), and 1,2-dichloroethane (DCE) were purchased from commercial sources and further dried with activated 4Å molecular sieves and degassed. The compounds 2-(3-oxo-2,3-dihydro-1*H*-inden-5-yl)acetic acid, oxalyl chloride, 6-(hydroxymethyl)-2,3-dihydro-1*H*-inden-1-one, 6-hydroxy-2,3-dihydro-1*H*-inden-1-one, 2-bromoacetyl bromide, 5-bromopentanoyl chloride, triphenylphosphine (Ph_3P), diisopropyl azodicarboxylate (DIAD), tetrabutylammonium bromide (TBAB), K_2CO_3, LiOH, and Et_3N were purchased from Fisher Scientific. $TiCl_4$ was purchased from TCI. The photoreactor, equipped with a fan to keep the reactor at ambient temperature during the reaction processes, used two blue LED lamps (Kessil PR160L 390 nm, 50% intensity) approximately 11.0 cm away. Yields refer to chromatographically and spectroscopically (^{1}H-NMR) homogeneous materials, unless otherwise stated. Reactions were monitored by thin layer chromatography (TLC) carried out on 0.25 mm E. Merck silica gel plates (60F-254) using UV light as the visualizing agent and basic aqueous potassium permanganate ($KMnO_4$), and heat as developing agents. E. Merck silica gel (60, particle size 0.043–0.063 mm) was used for flash column chromatography. NMR spectra were recorded on Bruker Avance 400, 500 or 700 MHz instruments and calibrated using residual undeuterated solvent as an internal reference ($CHCl_3$ @ 7.26 ppm ^{1}H NMR, 77.16 ppm ^{13}C NMR). The following abbreviations (or combinations thereof) were used to explain the multiplicities: s = singlet, d = doublet, t = triplet, q = quartet, m = multiplet, br = broad, comp = composite of magnetically non-equivalent protons. Mass spectra (MS) were recorded on LC/MS (Agilent Technologies 1260 Infinity II/6120 Quadrupole) or a time-of-flight matrix assisted laser desorption/ionization (MALDI-TOF) using a *trans*-2-[3-(4-*tert*-butylphenyl)-2-methyl-2-propenylidene]malononitrile (DCTB) matrix. Polymer samples were analyzed using a Tosoh EcoSEC HLC 8320GPC system with TSKgel SuperHZ-L columns eluting $CHCl_3$ containing 0.25% NEt_3 at a flow rate of 0.45 mL/min. All number-average molecular weights and dispersities were calculated from refractive index chromatograms using PStQuick Mp-M polystyrene standards. Thermogravimetric analyses (TGA) were performed under nitrogen atmosphere on a Pyris 1 TGA (PerkinElmer) at a heating rate of 10 °C/min. Differential scanning calorimetry (DSC) analyses were measured on a DSC 3+ STARe system (Mettler Toledo). The reported data were obtained from the third heating cycle at a heating rate of 10 °C/min. Melting points were measured on a MEL-TEMP II Laboratory Devices (uncorrected).

2. Synthesis of photoswitch monomers

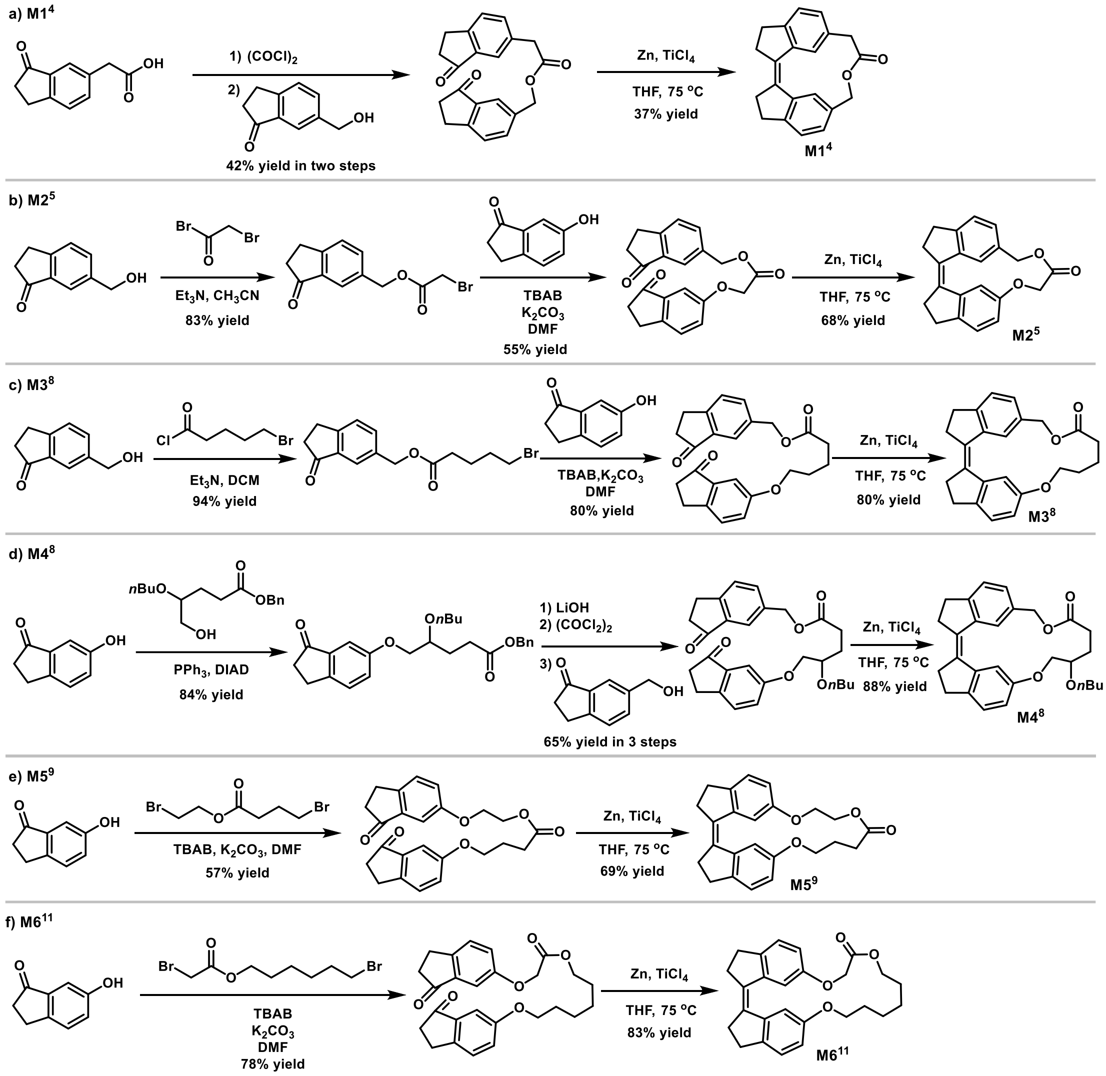

Scheme S1. Synthetic route of photoswitch monomers.

General procedure 1: Acyl chloride preparation

To a solution of acid precursor (1.0 equiv.) in DCM (0.2 M) at 0 °C was added oxalyl chloride (2.0 equiv.) dropwise, followed by the addition of 1-2 drops of DMF. The reaction mixture was stirred for 2 h and concentrated under reduced pressure to remove excess oxalyl chloride, affording the corresponding acyl chloride, which was used in the next step without further purification.

General procedure 2a: Esterification

To a solution of the alcohol substrate (1.0 equiv.) and pyridine (3.0 equiv.) in THF (0.2 M) at 0 °C was added the acyl chloride dropwise. The reaction mixture was then allowed to warm to rt and stirred overnight. After completion, the mixture was filtered through a Celite bed and concentrated under reduced pressure. The residue was extracted with ethyl acetate, and the combined organic layers were washed with brine, dried over sodium sulfate and concentrated in vacuo. The crude product was purified by column chromatography on silica gel (eluent: hexanes/ ethyl acetate = 2.5:1) to give the desired ester.

General procedure 2b: Esterification
To a solution of the alcohol substrate (1.0 equiv.), and Et_3N (2.0 equiv.) in DCM or CH_3CN (0.2 M) at 0 °C was added the commercially available acyl chloride or acyl bromide dropwise under N_2. The reaction mixture was stirred at rt overnight. After completion, the reaction was quenched with H_2O and extracted with DCM. The combined organic layers was washed with brine, dried over sodium sulfate, and concentrated in vacuo. The crude product was purified by column chromatography on silica gel (eluent: hexanes/ ethyl acetate = 10:1)
General procedure 3: Etherification
To a solution of 6-hydroxy-2,3-dihydro-1H-inden-1-one (1.0 equiv.) in DMF (0.3 M) was added alkyl bromide (1.0 equiv.), tetrabutylammonium bromide (TBAB, 0.1 equiv.), and K_2CO_3 (2.0 equiv.) under N_2. The reaction mixture was stirred at 65 °C overnight. After cooling to rt, the reaction was quenched with H_2O and extracted with DCM. The organic phase was washed with H_2O, dried over sodium sulfate, and concentrated in vacuo. The crude product was purified by column chromatography on silica gel (eluent: hexanes/ ethyl acetate = 2:1)
General procedure 4: McMurry reaction
Zinc (Zn) was activated by sequential washing with 2% HCl, deionized water, ethanol, acetone, and diethyl ether. To a stirred suspension of zinc powder (12 equiv.) in dry THF (0.01 M), $TiCl_4$ (6.0 equiv.) was added dropwise at 0 °C. The resulting slurry was heated to reflux and stirred for 1.5 h. A solution of di-indenone was added dropwise over 5 h using a syringe pump to the refluxing mixture. After the addition was complete, reflux continued for an additional 30 min. The reaction mixture was cooled to RT and quenched with a saturated aqueous solution of ammonium chloride. The mixture was extracted with DCM, and the combined organic layers were washed with brine, dried over sodium sulfate and concentrated in vacuo. The crude product was purified by column chromatography on silica gel (eluent: hexanes/ diethyl ether = 10:1) to give the desired monomer.
General procedure 5: Mitsunobu reaction
To a stirred solution of 6-hydroxy-2,3-dihydro-1H-inden-1-one (1.0 equiv.), benzyl 4-butoxy-5-hydroxypentanoate (1.0 equiv.), and PPh_3 (1.0 equiv.) in THF (0.3 M) was added diisopropylazodicarboxylate (DIAD, 1.0 equiv.) dropwise at rt. The reaction mixture was stirred overnight. After completion, the mixture was concentrated under reduced pressure, and the residue was purified by column chromatography on silica gel (eluent: hexanes/ diethyl ether = 5:1) to give the desired product.
General procedure 6: Hydrolysis reaction
To a stirred solution of benzyl 4-butoxy-5-((3-oxo-2,3-dihydro-1H-inden-5-yl)oxy)pentanoate (1.0 equiv.) in 1,4-dioxane/H_2O (3/1, 0.1 M) was added $LiOH \cdot H_2O$ (1.5 equiv.). The reaction mixture was stirred for 2 h at rt. After completion, the mixture was concentrated under reduced pressure, and the residue was purified by column chromatography on silica gel (DCM: MeOH = 40:1) to give the desired product.

(*Z*)-1,2,6,9,13,14-Hexahydro-8*H*-3,5:10,12-diethenodicyclopenta[*e,g*][1]oxacyclododecin-8-one.
Follow general procedures 1, 2a and 4.

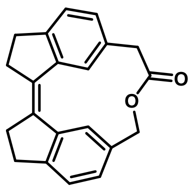

M1[4]

^{1}H NMR (700 MHz, chloroform-*d*) δ 7.80 (d, *J* = 6.2 Hz, 2H), 7.16 (d, *J* = 7.6 Hz, 1H), 7.14 (d, *J* = 7.6 Hz, 1H), 6.98 (dd, *J* = 7.6, 1.6 Hz, 1H), 6.95 (dd, *J* = 7.6, 1.7 Hz, 1H), 5.23 (s, 2H), 3.57 (s, 2H), 3.01 – 2.94 (comp, 4H), 2.80 – 2.73 (comp, 4H). **^{13}C NMR** (176 MHz, chloroform-*d*) δ 171.5, 146.3, 145.5, 140.8, 134.6, 134.3, 134.2, 130.7, 127.7, 125.88, 125.86, 125.6, 125.2, 124.2, 66.8, 41.9, 32.7, 32.4, 31.3, 31.2. Peak overlapping was observed. **MS (m/z)**: calcd for $C_{21}H_{18}NaO_2$, $[M+Na]^+$: 325.12; found, 325.4.

(*Z*)-2,10,14,15-Tetrahydro-1*H*-3,5:11,13-diethenodicyclopenta[*g,i*][1,4]dioxacyclotridecin-8-(7*H*)-one.

Follow general procedures 2b, 3 and 4.

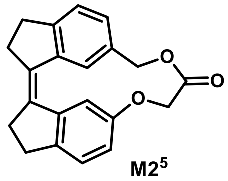

M2[5]

^{1}H NMR (700 MHz, chloroform-*d*) δ 8.23 – 8.17 (m, 1H), 7.29 (d, *J* = 2.6 Hz, 1H), 7.18 (comp, 2H), 7.03 (dd, *J* = 7.5, 1.7 Hz, 1H), 6.86 (dd, *J* = 8.3, 2.6 Hz, 1H), 5.40 (s, 2H), 4.62 (s, 2H), 2.98 (dd, *J* = 8.0, 5.5 Hz, 2H), 2.90 (dd, *J* = 8.0, 5.2 Hz, 2H), 2.83 – 2.75 (comp, 4H). **^{13}C NMR** (176 MHz, chloroform-*d*) δ 170.4, 154.9, 147.8, 141.4, 141.3, 141.0, 135.8, 135.4, 134.0, 126.41, 126.35, 124.6, 124.4, 117.0, 107.2, 67.2, 65.4, 34.3, 33.7, 30.5, 29.9. **MS (m/z)**: calcd for $C_{21}H_{19}O_3$, $[M+H]^+$: 319.13; found, 319.2. Overall three-step yield: 28%. The spectral data were in accordance with those reported in the literature.[1]

(*Z*)-2,7,8,9,10,13,17,18-octahydro-1*H*,11*H*-3,5:14,16-Diethenodicyclopenta[*j,l*][1,7]dioxacyclo-hexadecin-11-one.

Follow general procedures 2a, 3 and 4.

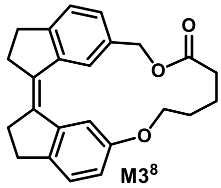

M3[8]

^{1}H NMR (700 MHz, chloroform-*d*) δ 8.13 – 8.08 (m, 1H), 7.62 (d, *J* = 2.4 Hz, 1H), 7.28 – 7.26 (m, 1H), 7.20 – 7.16 (m, 1H), 7.10 (dd, *J* = 7.6, 1.5 Hz, 1H), 6.77 (dd, *J* = 8.2, 2.4 Hz, 1H), 5.04 (s, 2H), 4.24 – 3.95 (m, 2H), 2.99 (dd, *J* = 8.2, 5.4 Hz, 2H), 2.92 (dd, *J* = 8.2, 5.1 Hz, 2H), 2.87 – 2.75 (comp, 4H), 2.55 – 2.32 (m, 2H), 1.95 – 1.78 (comp, 4H). **^{13}C NMR** (126 MHz, chloroform-*d*) δ 173.5, 157.5, 148.8, 141.6, 141.5, 141.1, 136.2, 134.6, 133.3, 127.8, 125.9, 125.2, 123.4, 116.7, 109.2, 68.4, 66.9, 35.6, 35.3, 34.2, 30.7, 30.1, 28.5, 21.3. **MS (m/z)**: calcd for $C_{24}H_{25}O_3$, $[M+H]^+$: 361.18; found, 361.2.

(*Z*)-8-Butoxy-2,7,8,9,10,13,17,18-octahydro-1*H*,11*H*-3,5:14,16-diethenodicyclopenta[*j,l*][1,7]-dioxacyclohexadecin-11-one

Follow general procedures 5, 6, 1, 2a and 4.

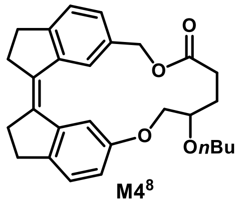

M4[8]

^{1}H NMR (700 MHz, chloroform-*d*) δ 8.14 – 8.10 (m, 1H), 7.64 (d, *J* = 2.4 Hz, 1H), 7.27 (d, *J* = 7.1 Hz, 1H), 7.18 (d, *J* = 8.2 Hz, 1H), 7.09 (dd, *J* = 7.6, 1.5 Hz, 1H), 6.79 (dd, *J* = 8.2, 2.4 Hz, 1H), 5.10 (d, *J* = 12.2 Hz, 1H), 4.96 (d, *J* = 12.2 Hz, 1H), 4.09 (dd, *J* = 10.9, 6.2 Hz, 1H), 3.96 (dd, *J* = 10.9, 5.0 Hz, 1H), 3.81 – 3.73 (m, 1H), 3.53 (dt, *J* = 9.2, 6.6 Hz, 1H), 3.46 (dt, *J* = 9.2, 6.7 Hz, 1H), 3.04 – 2.95 (m, 2H), 2.94 – 2.88 (m, 2H), 2.86 – 2.77 (comp, 4H), 2.60 – 2.46 (m, 2H), 2.09 (ddt, *J* = 14.1, 8.9, 5.1 Hz, 1H), 2.01 – 1.92 (m, 1H), 1.54 – 1.43 (m, 2H), 1.36 – 1.28 (m, 2H), 0.87 (t, *J* = 7.4 Hz, 3H). **^{13}C NMR** (176 MHz, chloroform-*d*) δ 173.5, 157.5, 148.7, 141.6, 141.4, 141.3, 136.1, 134.7, 133.3, 127.8, 125.9, 125.2, 123.4, 116.5,

109.6, 76.7, 71.0, 70.2, 66.9, 35.5, 35.2, 32.3, 30.7, 30.1, 30.1, 26.9, 19.4, 14.0. **MS (m/z)**: calcd for $C_{28}H_{33}O_4$, $[M+H]^+$: 433.24; found, 433.3.

(*Z*)-1,2,7,8,12,13,18,19-Octahydro-3,5:15,17-diethenodicyclopenta[*l,n*][1,4,9]trioxacyclohept-adecin-10(11*H*)-one
Follow general procedures 3 and 4.

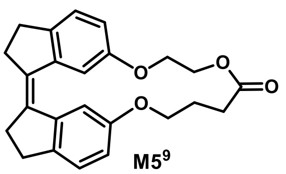
M5[9]

¹H NMR (700 MHz, chloroform-*d*) δ 7.80 (d, *J* = 2.4 Hz, 1H), 7.63 (d, *J* = 2.4 Hz, 1H), 7.19 (d, *J* = 8.1 Hz, 1H), 7.15 (d, *J* = 8.2 Hz, 1H), 6.75 (dd, *J* = 8.2, 2.4 Hz, 1H), 6.68 (dd, *J* = 8.1, 2.4 Hz, 1H), 4.53 – 4.45 (m, 2H), 4.20 – 4.18 (m, 2H), 3.94 (t, *J* = 7.3 Hz, 2H), 2.96 – 2.88 (comp, 4H), 2.84 – 2.78 (comp, 4H), 2.60 – 2.47 (m, 2H), 2.29 – 2.16 (m, 2H). **¹³C NMR** (126 MHz, chloroform-*d*) δ 173.1, 157.3, 157.0, 141.9, 141.3, 141.2, 140.7, 136.3, 134.6, 125.8, 125.5, 116.1, 112.7, 111.9, 107.8, 67.5, 66.8, 62.3, 35.14, 35.13, 31.1, 29.9, 25.2. Peak overlapping was observed. **MS (m/z)**: calcd for $C_{24}H_{25}O_4$, $[M+H]^+$: 377.17; found, 377.2.

(*Z*)-1,2,10,11,12,13,14,15,20,21-Decahydro-3,5:17,19-diethenodicyclopenta[*n,p*][1,4,11]trioxa-cyclononadecin-8(7*H*)-one.
Follow general procedures 3 and 4.

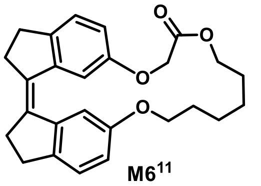
M6[11]

¹H NMR (500 MHz, chloroform-*d*) δ 7.73 (d, *J* = 2.6 Hz, 1H), 7.66 (d, *J* = 2.5 Hz, 1H), 7.21 – 7.14 (comp, 2H), 6.82 (dd, *J* = 8.2, 2.6 Hz, 1H), 6.75 (dd, *J* = 8.2, 2.5 Hz, 1H), 4.60 (s, 2H), 4.30 (t, *J* = 5.3 Hz, 2H), 3.96 (t, *J* = 5.9 Hz, 2H), 3.07 – 2.88 (comp, 4H), 2.88 – 2.79 (comp, 4H), 1.85 – 1.67 (comp, 4H), 1.67 – 1.50 (comp, 4H). **¹³C NMR** (176 MHz, chloroform-*d*) δ 168.9, 157.8, 156.3, 141.9, 141.8, 141.6, 140.7, 136.0, 135.3, 125.8, 125.7, 114.1, 113.5, 109.8, 109.7, 67.3, 66.23, 66.17, 35.6, 35.5, 29.88, 29.87, 28.8, 27.5, 26.2, 26.1. **MS (m/z)**: calcd for $C_{26}H_{29}O_4$, $[M+H]^+$: 405.21; found, 405.3. The proton spectral data were in accordance with those reported in the literature.[1]

(3-Oxo-2,3-dihydro-1*H*-inden-5-yl)methyl 2-(3-oxo-2,3-dihydro-1*H*-inden-5-yl)acetate.

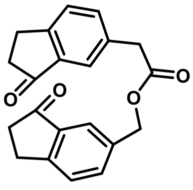

¹H NMR (400 MHz, chloroform-*d*) δ 7.69 – 7.59 (comp, 2H), 7.57 – 7.50 (comp, 2H), 7.49 – 7.42 (comp, 2H), 5.17 (s, 2H), 3.73 (s, 2H), 3.21 – 3.03 (comp, 4H), 2.82 – 2.60 (comp, 4H). **¹³C NMR** (126 MHz, chloroform-*d*) δ 206.9, 206.7, 171.0, 155.3, 154.4, 137.7, 137.6, 136.0, 135.4, 134.6, 133.3, 127.1, 124.5, 123.3, 66.2, 40.9, 36.7, 36.6, 25.8, 25.7. Peak overlapping was observed. **MS (m/z)**: calcd for $C_{21}H_{19}O_4$, $[M+H]^+$: 335.13; found, 335.2.

(3-Oxo-2,3-dihydro-1*H*-inden-5-yl)methyl 2-((3-oxo-2,3-dihydro-1*H*-inden-5-yl)oxy)acetate.

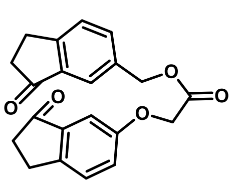

¹H NMR (700 MHz, chloroform-*d*) δ 7.70 (d, *J* = 1.0 Hz, 1H), 7.58 (dd, *J* = 7.9, 1.7 Hz, 1H), 7.49 (dd, *J* = 7.8, 1.0 Hz, 1H), 7.42 – 7.38 (m, 1H), 7.28 – 7.26 (m, 1H), 7.08 (d, *J* = 2.6 Hz, 1H), 5.28 (s, 2H), 4.71 (s, 2H), 3.18 – 3.11 (m, 2H), 3.11 – 3.06 (m, 2H), 2.74 – 2.68 (comp, 4H). **¹³C NMR** (126 MHz, chloroform-*d*) δ 206.8, 206.6, 168.4, 157.6, 155.5, 149.1, 138.3, 137.7, 134.8, 134.8, 127.9, 127.2, 124.6, 123.6, 105.9, 66.6, 65.4, 37.1, 36.6, 25.9, 25.3. **MS (m/z)**: calcd for $C_{21}H_{19}O_5$, $[M+H]^+$: 351.12; found, 351.1.

(3-Oxo-2,3-dihydro-1*H*-inden-5-yl)methyl 5-bromopentanoate.

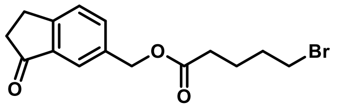

¹H NMR (500 MHz, chloroform-*d*) δ 7.74 (s, 1H), 7.58 (d, *J* = 7.9 Hz, 1H), 7.48 (d, *J* = 7.9 Hz, 1H), 5.16 (s, 2H), 3.40 (t, *J* = 6.5 Hz, 2H), 3.19 – 3.08 (m, 2H), 2.72 (dd, *J* = 6.8, 4.9 Hz, 2H), 2.40 (t, *J* = 7.2 Hz, 2H), 1.96 – 1.86 (m, 2H), 1.85 – 1.76 (m, 2H). **¹³C NMR** (126 MHz, chloroform-*d*) δ 206.8, 173.0, 155.3, 137.6, 135.7, 134.6, 127.1, 123.3, 65.7, 36.6, 33.4, 33.1, 32.1, 25.8, 23.6. **MS (m/z)**: calcd for $C_{15}H_{18}BrO_3$, $[M+H]^+$: 325.04; found, 325.1.

(3-Oxo-2,3-dihydro-1*H*-inden-5-yl)methyl 5-((3-oxo-2,3-dihydro-1*H*-inden-5-yl)oxy)pentanoate.

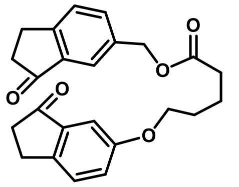

¹H NMR (500 MHz, chloroform-*d*) δ 7.74 (s, 1H), 7.61 – 7.55 (m, 1H), 7.48 (d, *J* = 7.9 Hz, 1H), 7.35 (d, *J* = 8.1 Hz, 1H), 7.20 – 7.12 (comp, 2H), 5.16 (s, 2H), 3.98 (s, 2H), 3.19 – 3.11 (m, 2H), 3.08 – 3.03 (m, 2H), 2.76 – 2.64 (comp, 4H), 2.50 – 2.42 (m, 2H), 1.90 – 1.77 (comp, 4H). **¹³C NMR** (126 MHz, chloroform-*d*) δ 207.3, 206.9, 173.2, 158.7, 155.2, 148.1, 138.2, 137.5, 135.7, 134.6, 127.5, 127.0, 124.5, 123.3, 105.7, 67.8, 65.6, 37.1, 36.6, 33.9, 28.6, 25.8, 25.2, 21.7. **MS (m/z)**: calcd for $C_{24}H_{25}O_5$, $[M+H]^+$: 393.17; found, 393.2.

Benzyl 4-butoxy-5-((3-oxo-2,3-dihydro-1*H*-inden-5-yl)oxy)pentanoate.

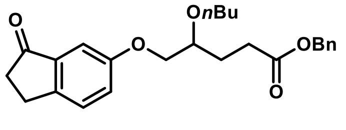

¹H NMR (700 MHz, chloroform-*d*) δ 7.41 – 7.28 (comp, 6H), 7.21 – 7.13 (comp, 2H), 5.12 (s, 2H), 4.00 (dd, *J* = 9.6, 5.3 Hz, 1H), 3.95 (dd, *J* = 9.6, 4.8 Hz, 1H), 3.69 – 3.58 (m, 2H), 3.45 (dt, *J* = 9.1, 6.6 Hz, 1H), 3.18 – 3.00 (m, 2H), 2.71 (dd, *J* = 6.5, 4.8 Hz, 2H), 2.60 – 2.43 (m, 2H), 2.02 (dtd, *J* = 15.0, 7.7, 4.1 Hz, 1H), 1.92 (dtd, *J* = 14.4, 8.1, 6.1 Hz, 1H), 1.58 – 1.47 (m, 2H), 1.39 – 1.31 (m, 2H), 0.89 (t, *J* = 7.4 Hz, 3H). **¹³C NMR** (176 MHz, chloroform-*d*) δ 207.1, 173.4, 158.6, 148.3, 138.3, 136.1, 128.7, 128.37, 128.36, 127.5, 124.5, 105.8, 76.7, 70.5, 70.3, 66.4, 37.1, 32.3, 30.3, 27.4, 25.3, 19.4, 14.0. **MS (m/z)**: calcd for $C_{25}H_{31}O_5$, $[M+H]^+$: 411.22; found, 411.2.

(3-Oxo-2,3-dihydro-1H-inden-5-yl)methyl 4-butoxy-5-((3-oxo-2,3-dihydro-1H-inden-5-yl)oxy)pentanoate.

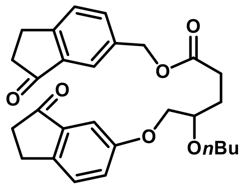

¹H NMR (700 MHz, chloroform-*d*) δ 7.74 (s, 1H), 7.58 (d, *J* = 9.0 Hz, 1H), 7.48 (d, *J* = 7.9 Hz, 1H), 7.36 (d, *J* = 8.3 Hz, 1H), 7.19 (dd, *J* = 8.3, 2.5 Hz, 1H), 7.16 (d, *J* = 2.4 Hz, 1H), 5.16 (s, 2H), 4.00 (dd, *J* = 9.7, 5.2 Hz, 1H), 3.94 (dd, *J* = 9.7, 4.9 Hz, 1H), 3.68 – 3.60 (m, 2H), 3.46 (dt, *J* = 9.2, 6.7 Hz, 1H), 3.16 – 3.12 (m, 2H), 3.09 – 3.03 (m, 2H), 2.76 – 2.67 (comp, 4H), 2.59 – 2.46 (m, 2H), 2.04 – 1.99 (m, 1H), 1.92 (dtd, *J* = 14.4, 8.3, 6.2 Hz, 1H), 1.56 – 1.47 (m, 2H), 1.39 – 1.30 (m, 2H), 0.89 (t, *J* = 7.4 Hz, 3H). **¹³C NMR** (176 MHz, chloroform-*d*) δ 207.1, 206.7, 173.3, 158.6, 155.2, 148.3, 138.3, 137.6, 135.7, 134.6, 127.5, 127.1, 124.5, 123.3, 105.9, 76.7, 70.5, 70.2, 65.7, 37.1, 36.6, 32.3, 30.2, 27.4, 25.8, 25.3, 19.4, 14.0. **MS (m/z)**: calcd for $C_{28}H_{33}O_6$, $[M+H]^+$: 465.23; found, 465.3.

2-((3-Oxo-2,3-dihydro-1*H*-inden-5-yl)oxy)ethyl 4-((3-oxo-2,3-dihydro-1*H*-inden-5-yl)oxy)butanoate.

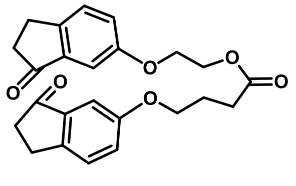

¹H NMR (500 MHz, chloroform-*d*) δ 7.41 – 7.32 (comp, 2H), 7.22 – 7.12 (comp, 4H), 4.46 (t, *J* = 4.6 Hz, 2H), 4.19 (t, *J* = 4.6 Hz, 2H), 4.02 (t, *J* = 6.2 Hz, 2H), 3.11 – 3.02 (comp, 4H), 2.76 – 2.67 (comp, 4H), 2.58 (t, *J* = 7.3 Hz, 2H), 2.14 (p, *J* = 6.6 Hz, 2H). **¹³C NMR** (126 MHz,

chloroform-*d*) δ 207.1, 207.0, 173.1, 158.6, 158.3, 148.5, 148.2, 138.4, 138.3, 127.7, 127.5, 124.6, 124.4, 105.9, 105.8, 67.2, 66.4, 62.8, 37.1, 30.8, 30.8, 25.3, 25.3, 24.6. **MS (m/z)**: calcd for $C_{24}H_{25}O_6$, $[M+H]^+$: 409.16; found, 409.2.

6-((3-Oxo-2,3-dihydro-1*H*-inden-5-yl)oxy)hexyl 2-((3-oxo-2,3-dihydro-1*H*-inden-5-yl)oxy)acetate.

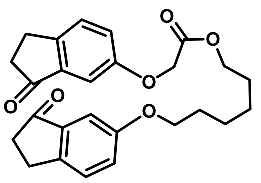

^{1}H NMR (500 MHz, chloroform-*d*) δ 7.39 (d, *J* = 8.4 Hz, 1H), 7.35 (d, *J* = 9.2 Hz, 1H), 7.30 – 7.25 (m, 1H), 7.20 – 7.14 (comp, 2H), 7.12 (d, *J* = 2.6 Hz, 1H), 4.66 (s, 2H), 4.21 (t, *J* = 6.6 Hz, 2H), 3.97 (t, *J* = 6.4 Hz, 2H), 3.17 – 3.00 (comp, 4H), 2.76 – 2.66 (comp, 4H), 1.82 – 1.75 (m, 2H), 1.70 (p, *J* = 6.8 Hz, 2H), 1.53 – 1.44 (m, 2H), 1.44 – 1.35 (m, 2H). The proton spectral data were in accordance with those reported in the literature.[1] **^{13}C NMR** (126 MHz, chloroform-*d*) δ 207.3, 206.8, 168.7, 158.9, 157.7, 149.0, 148.0, 138.3, 127.8, 127.5, 124.6, 124.6, 105.9, 105.7, 68.3, 65.5, 65.5, 37.14, 37.11, 29.1, 28.6, 25.8, 25.7, 25.3, 25.2. Peak overlapping was observed. **MS (m/z)**: calcd for $C_{26}H_{29}O_6$, $[M+H]^+$: 437.20; found, 437.2.

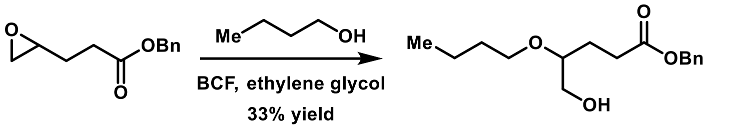

The epoxide alcoholysis was performed following a literature-reported procedure with slight modifications.[2]

To a stirred solution of tris(pentafluorophenyl)borane (BCF, 0.5 mol%), ethylene glycol (2 mol%), H_2O (6 mol%) in 1-butanol (20.0 equiv.) was added benzyl 3-(oxiran-2-yl)propanoate (1.0 equiv.). The reaction mixture was stirred at 60 °C for 2 h. After completion, the mixture was concentrated under reduced pressure, and the residue was purified by column chromatography on silica gel (eluent: hexanes/ ethyl acetate = 4:1) to give the desired product.

Benzyl 4-butoxy-5-hydroxypentanoate.

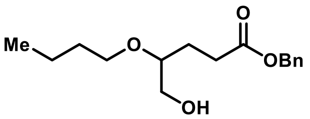

^{1}H NMR (500 MHz, chloroform-*d*) δ 7.40 – 7.29 (comp, 5H), 5.16 – 5.08 (m, 2H), 3.65 (dd, *J* = 11.5, 3.9 Hz, 1H), 3.53 – 3.43 (comp, 3H), 3.41 – 3.30 (m, 1H), 2.45 (t, *J* = 7.4 Hz, 2H), 1.96 – 1.78 (comp, 3H), 1.58 – 1.48 (m, 2H), 1.42 – 1.30 (m, 2H), 0.91 (t, *J* = 7.4 Hz, 3H). **^{13}C NMR** (126 MHz, chloroform-*d*) δ 173.5, 136.1, 128.7, 128.4, 78.7, 69.7, 66.5, 63.9, 32.3, 30.1, 26.1, 19.5, 14.0. **MS (m/z)**: calcd for $C_{16}H_{25}O_4$, $[M+H]^+$: 282.17; found, 281.2.

3. Photoisomerization of monomers M1[4]-M6[11].

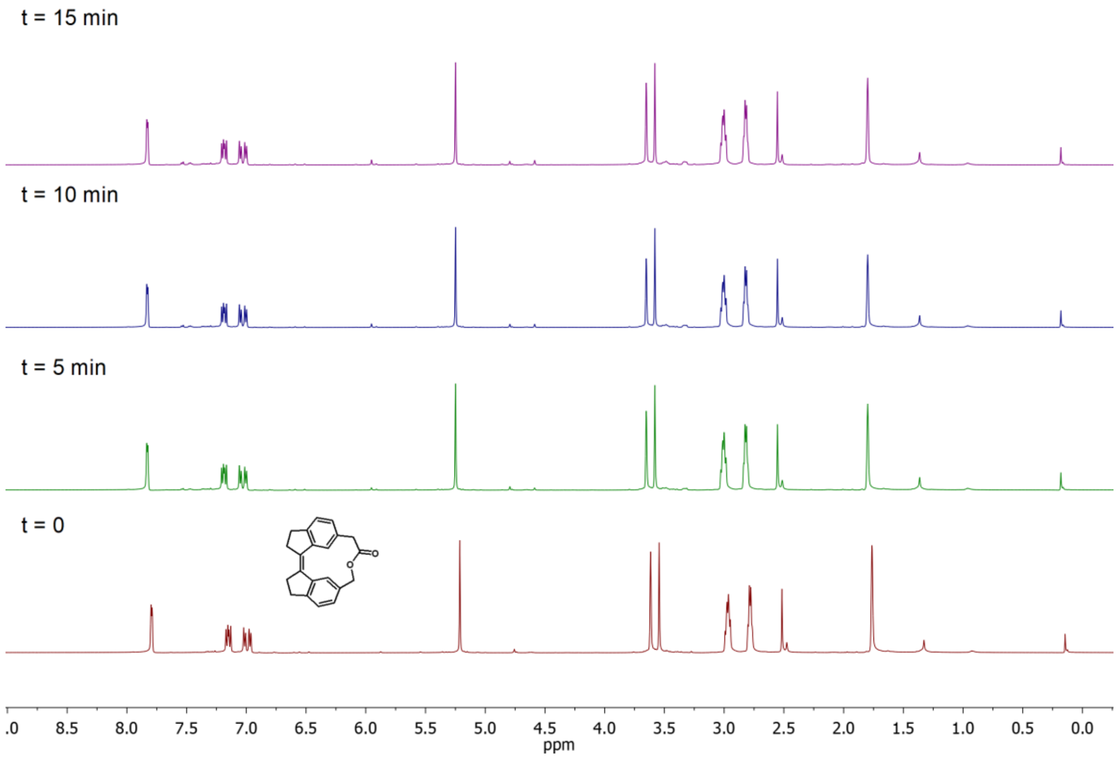

Figure S1. Time-dependent ^{1}H-NMR spectra of M1[4] under 390 nm irradiation in THF-d_8 (0.04 M).

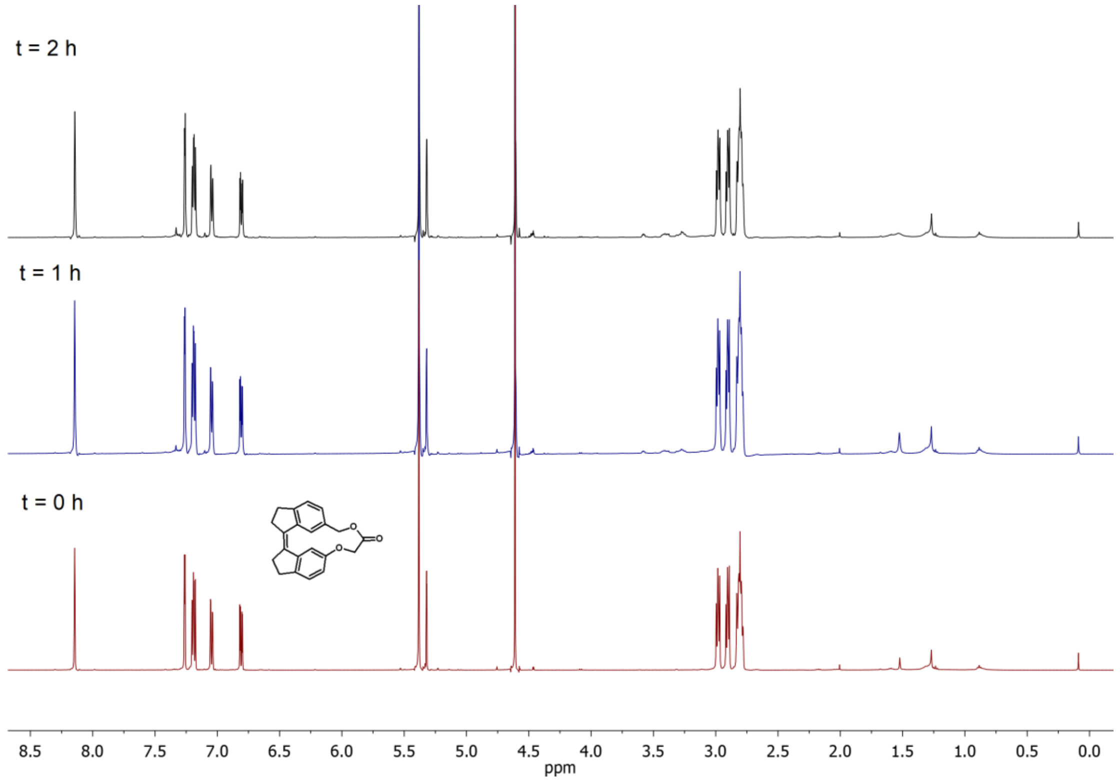

Figure S2. Time-dependent ^{1}H-NMR spectra of M2[5] under 390 nm irradiation in CD_2Cl_2 (0.04 M).

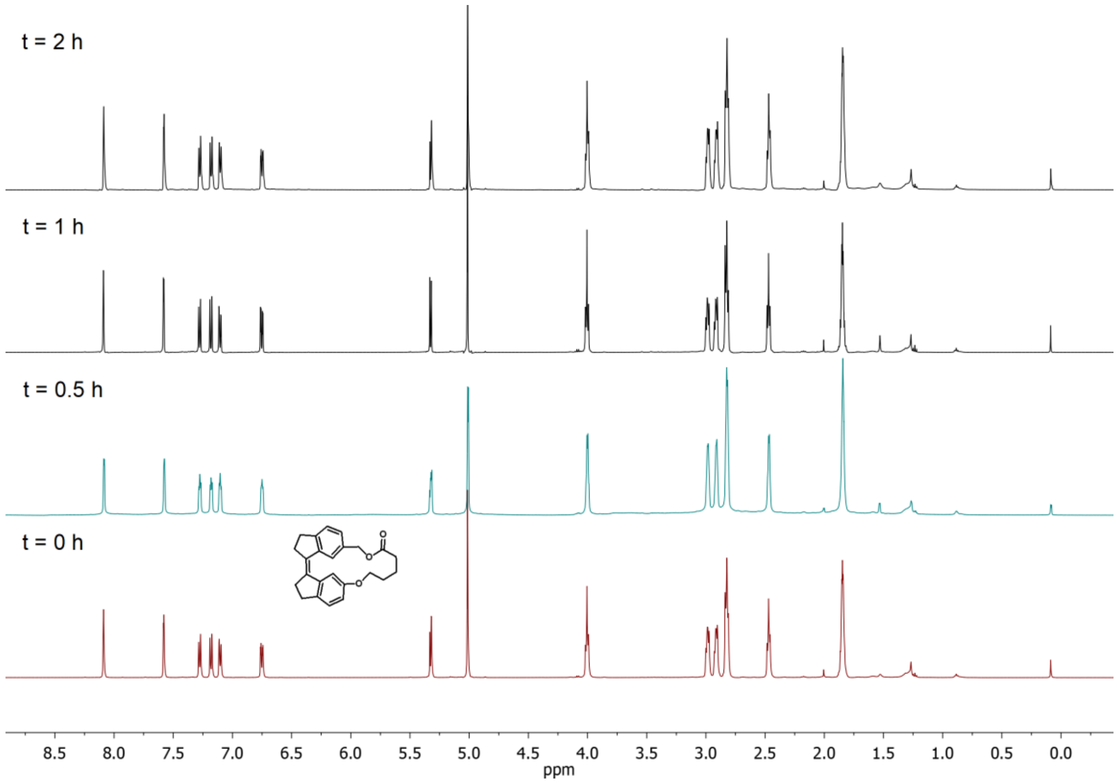

Figure S3. Time-dependent ^{1}H-NMR spectra of M3[8] under 390 nm irradiation in CD_2Cl_2 (0.04 M).

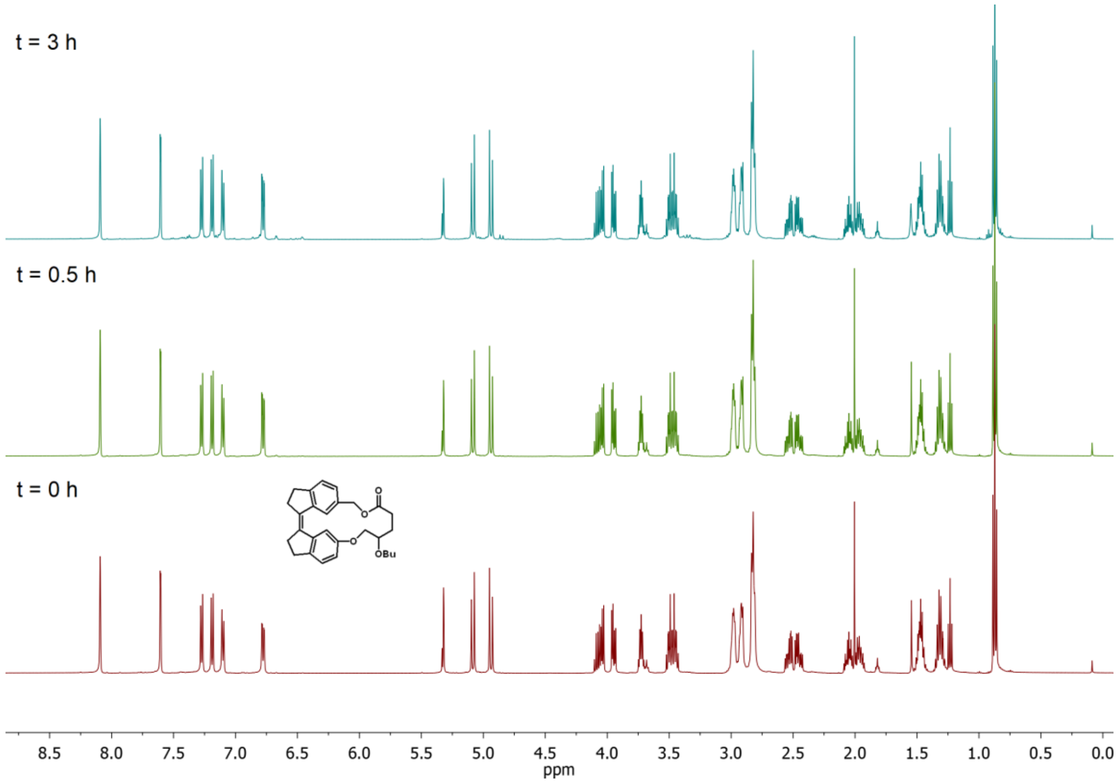

Figure S4. Time-dependent ^{1}H-NMR spectra of M4[8] under 390 nm irradiation in CD_2Cl_2 (0.04 M).

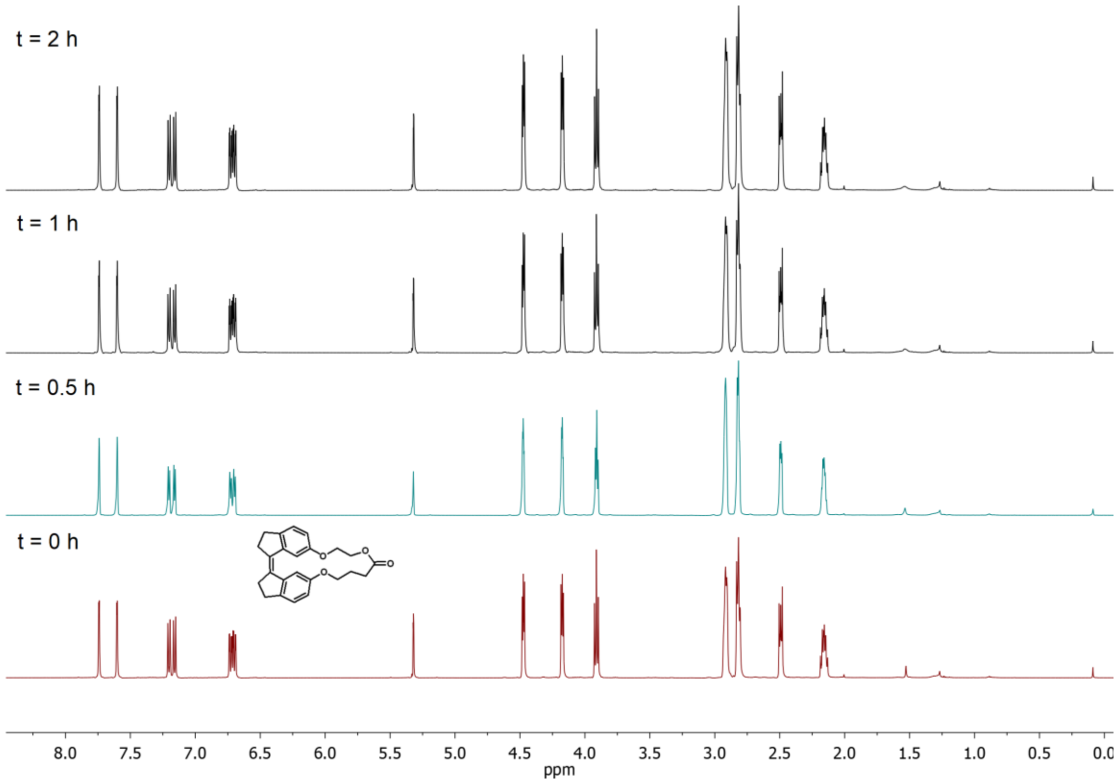

Figure S5. Time-dependent ^{1}H-NMR spectra of M5[9] under 390 nm irradiation in CD_2Cl_2 (0.04 M).

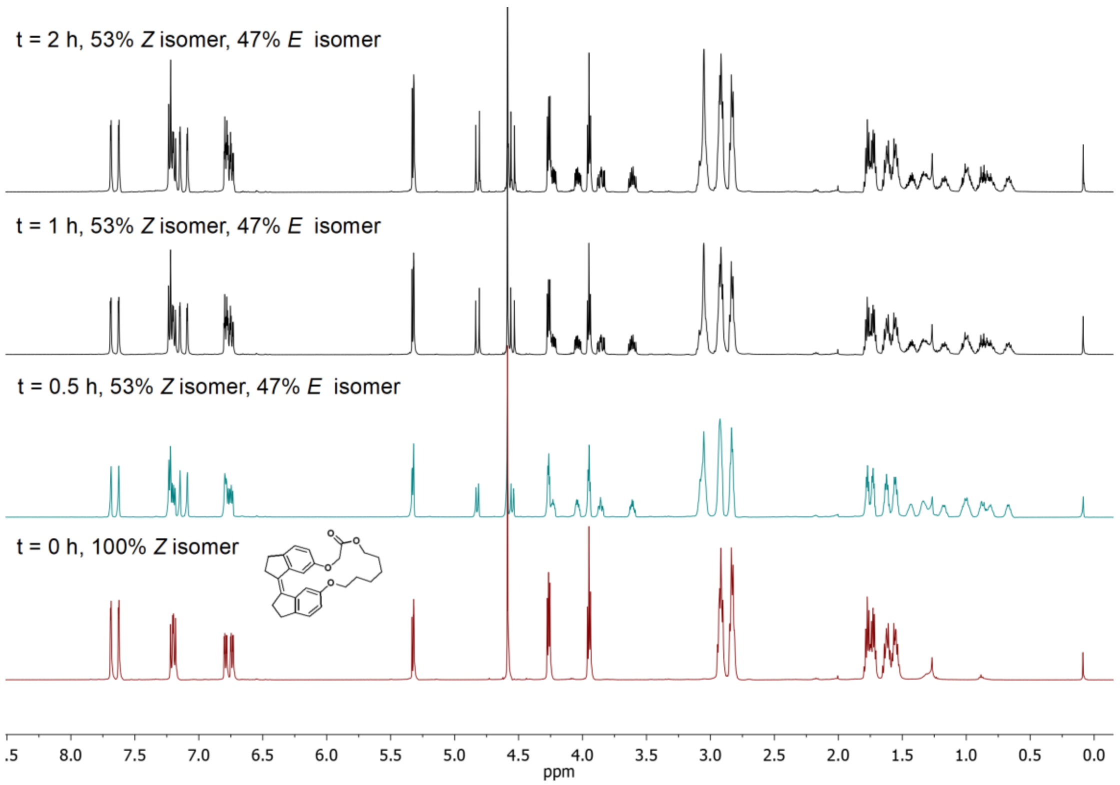

Figure S6. Time-dependent ^{1}H-NMR spectra of M6[11] under 390 nm irradiation in CD_2Cl_2 (0.04 M).

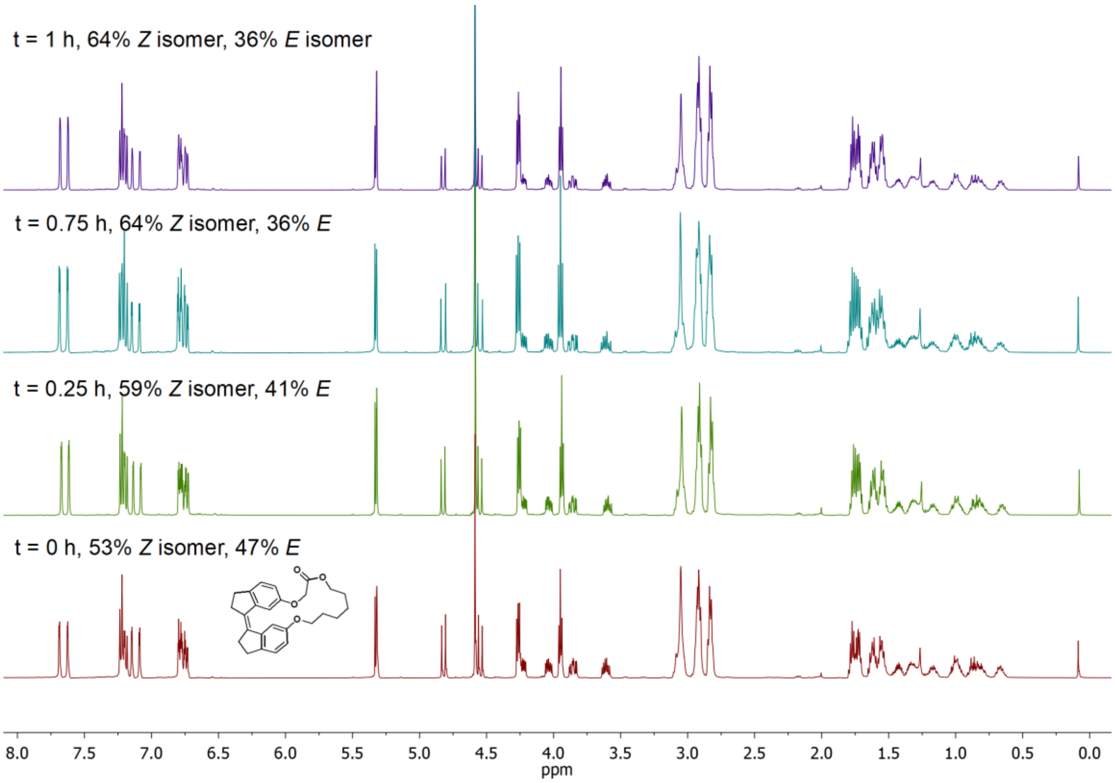

Figure S7. Time-dependent ^{1}H-NMR spectra of M6^{11} under 365 nm irradiation in CD_2Cl_2 (0.04 M).

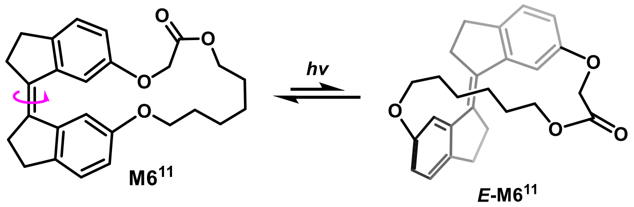

(*E*)-4,5,6,7,8,9,17,18,19,20-decahydro-2,21-(epiethane[1,2]diylidene)-14,16-ethenodicyclopenta[*n,p*][1,4,11]trioxacyclononadecin-11(12*H*)-one.

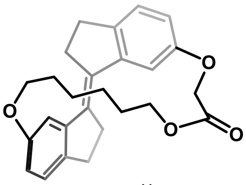

***E*-M6^{11}**

^{1}H NMR (500 MHz, chloroform-*d*) δ 7.25 – 7.20 (m, 2H), 7.15 (d, *J* = 2.3 Hz, 1H), 7.08 (d, *J* = 2.5 Hz, 1H), 6.85 (dd, *J* = 8.1, 2.5 Hz, 1H), 6.79 (dd, *J* = 8.1, 2.3 Hz, 1H), 4.82 (d, *J* = 14.2 Hz, 1H), 4.58 (d, *J* = 14.2 Hz, 1H), 4.24 (ddd, *J* = 11.6, 7.1, 4.0 Hz, 1H), 4.06 (ddd, *J* = 11.6, 6.7, 4.2 Hz, 1H), 3.87 (ddd, *J* = 12.5, 10.6, 3.9 Hz, 1H), 3.66 (td, *J* = 11.1, 5.6 Hz, 1H), 3.15 – 2.96 (comp, 6H), 2.95 – 2.82 (m, 2H), 1.50 – 1.40 (m, 1H), 1.38 – 1.29 (m, 1H), 1.23 – 1.13 (m, 1H), 1.08 – 0.95 (m, 2H), 0.93 – 0.77 (m, 2H), 0.76 – 0.63 (m, 1H). **^{13}C NMR** (126 MHz, chloroform-*d*) δ 169.2, 157.1, 156.6, 144.8, 144.7, 141.7, 141.3, 135.1, 134.9, 126.1, 126.0, 119.2, 117.7, 114.3, 108.8, 70.4, 67.9, 65.6, 36.0, 35.7, 31.9, 31.8, 29.1, 28.6, 26.6, 24.4. **MS (m/z)**: calcd for $C_{26}H_{29}O_4$, $[M+H]^+$: 405.21; found, 405.3.

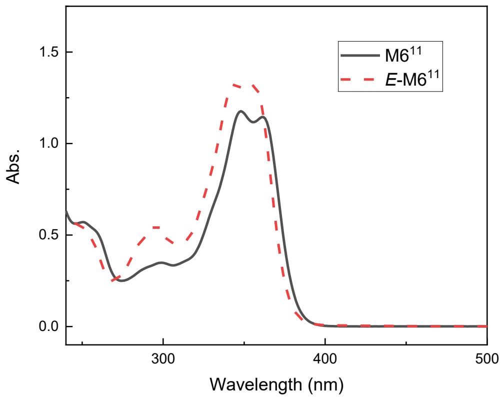

Figure S8. UV-Vis spectra of *Z*-M6[11] and *E*-M6[11]

4. Optimization of the polymerization conditions

Table S1. Investigation of ring-opening exchange reaction conditions of M2[5]

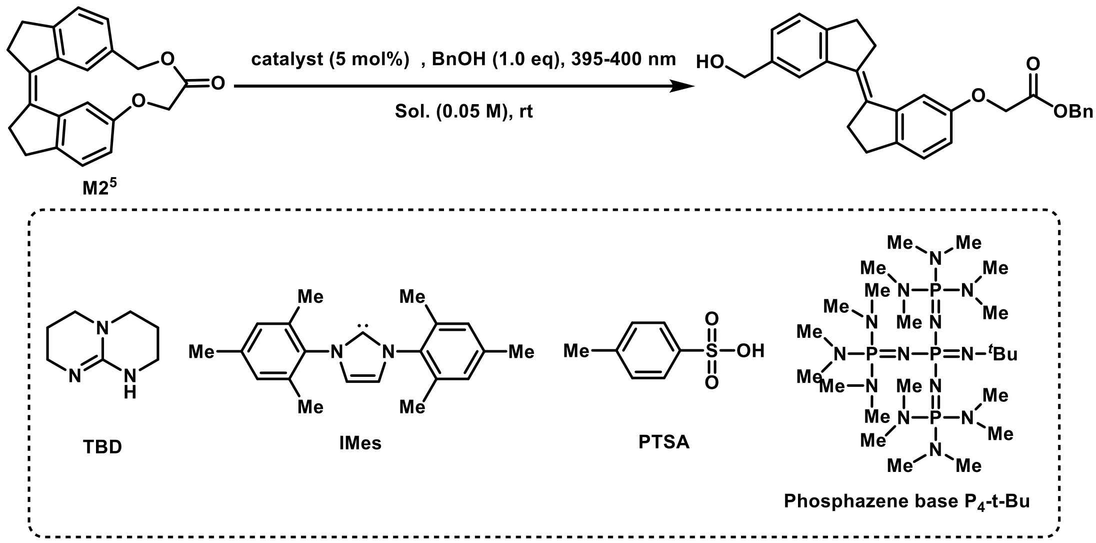

entry	catalyst	solvent	time (h)	conv. (%)^a
1	TBD	THF	23	<10
2	TBD	DCM	23	<10
3	IMes	THF	16	<5
4	PTSA	THF	16	<5
5	P_4-t-Bu	THF	21	<5

^{a}Conversions were determined by ^{1}H NMR spectroscopic analysis of the reaction mixture.

Table S2. Investigation of polymerization conditions for M3[8][a]

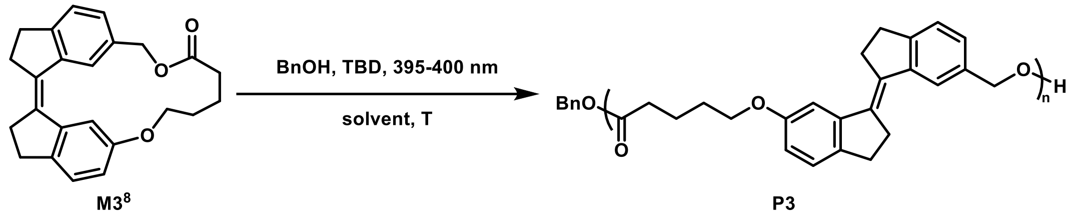

entry	solvent	conc. (M)	time (h)	conv. (%)[b]	$M_{n,SEC}$ (kDa)[c]	$Đ^c$
1	DCM	0.2	88	30	1.5	1.44
2	DCM	0.5	23	55	2.3	1.55
3	DCM	1	18	75	2.7	1.66
4[d]	DCM	1	16	85	3.6	1.91
5	DCE	1	18	17	-	-
6	THF	0.5	44	75	2.2	1.79
7	DMF	0.5	18	24	1.7	1.1
8	Ph_2O	0.5	18	11	-	-
9	o-xylene	0.5	18	80	2.0	1.92
10	1,2-dichlorobenzene	0.2	40	39	1.5	1.47
11	toluene	0.2	48	65	2.4	1.58
12[e]	toluene	0.5	16	97	2.2	1.50
13	THF/toluene (1/1)	0.2	14	17	-	-
14	$PhCF_3$	0.2	18	56	2.4	1.89

[a]$[M]_0/[I]_0/[catalyst]_0$ = 10/1/1. [b]Conversions were determined by ^1H NMR spectroscopic analysis of the reaction mixture. [c]Molecular weights ($M_{n,SEC}$)and dispersities ($Đ$)were determined by size-exclusion chromatography. [d]390 nm. [e]100℃.

Table S3. Investigation of solvents for the polymerization of M4[8][a]

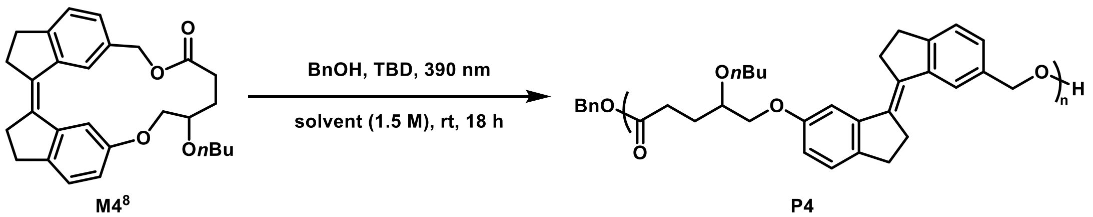

entry	solvent	conv. (%)[b]	$M_{n,SEC}$ (kDa)[c]	$Đ^c$
1	DCM	54	6.2	2.09
2	THF	31	2.2	1.46
3	DMF	9	0.99	1.16
4	o-Xylene	25	2.09	1.48

[a]$[M]_0/[I]_0/[catalyst]_0$ = 20/1/1. [b]Conversions were determined by ^1H NMR spectroscopic analysis of the reaction mixture. [c]Molecular weights ($M_{n,SEC}$)and dispersities ($Đ$)were determined by size-exclusion chromatography.

Table S4. Investigation of light for the polymerization of M4[8][a]

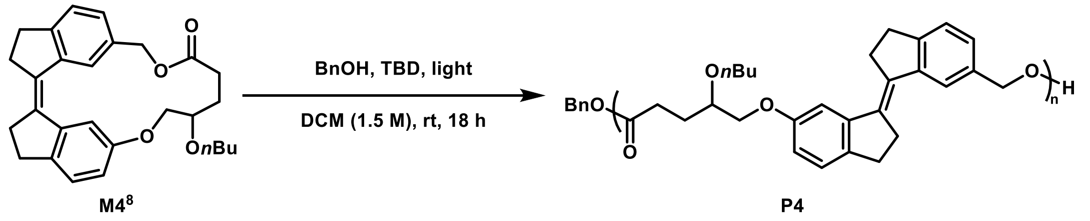

entry	light	conv. (%)[b]	$M_{n,SEC}$ (kDa)[c]	$Đ$[c]
1	370 nm	30	3.2	1.42
2	427 nm	17	2.4	1.29
3	white light	7	1.5	1.21
4	without light	<5%	-	-

[a][M]$_0$/[I]$_0$/[catalyst]$_0$ = 20/1/1. [b]Conversions were determined by ^{1}H NMR spectroscopic analysis of the reaction mixture. [c]Molecular weights ($M_{n,SEC}$)and dispersities ($Đ$)were determined by size-exclusion chromatography.

Table S5. Investigation catalysts and initiators for the polymerization of M4[8][a]

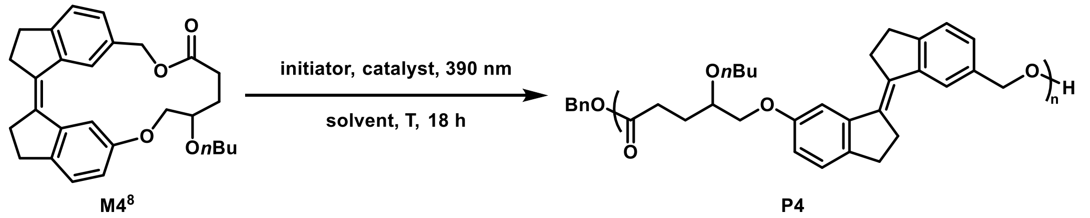

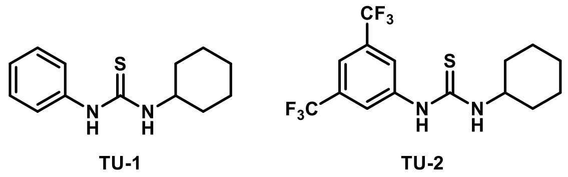

entry	initiator	catalyst	solvent	temp. (℃)	conc. (M)	conv. (%)[b]	$M_{n,SEC}$ (kDa)[c]	$Đ$[c]
1	BnOH	DBU/TU-2[d]	DCM	rt	1.25	10	0.85	1.12
2	BnOH	Sn(Oct)$_2$	toluene	150	1.0	52	5.4	2.62
3	NaOMe	-	DCM	rt	1.5	<2	-	-
4	NaOMe	-	toluene	rt	1.5	4	-	-
5	NaOMe	TU-1[e]	DCM	rt	1.5	<2	-	-
6	NaOMe	TU-2[e]	toluene	rt	1.5	7	-	-

[a][M]$_0$/[I]$_0$/[catalyst]$_0$ = 20/1/1. [b]Conversions were determined by ^{1}H NMR spectroscopic analysis of the reaction mixture. [c]Molecular weights ($M_{n,SEC}$)and dispersities ($Đ$)were determined by size-exclusion chromatography. [d]0.16 equiv. DBU and TU2. [e]0.15 equiv. thiourea (TU).

Table S6. Investigation of the amount of TBD for the polymerization of M4[8][a]

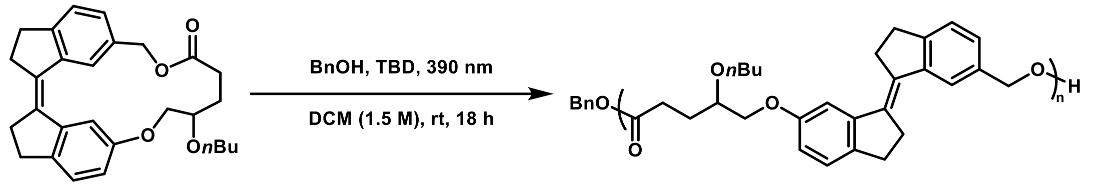

M4[8]

P4

entry	TBD (equiv.)	conv. (%)[b]	$M_{n,SEC}$ (kDa)[c]	*Đ*[c]
1	0.05	54	6.2	2.09
2	0.1	73	5.6	2.18
3	0.15	83	6.9	2.04
4	0.20	88	6.2	2.11
5	0.25	90	5.4	1.90

[a]$[M]_0/[I]_0/[catalyst]_0$ = 20/1/x. [b]Conversions were determined by ^{1}H NMR spectroscopic analysis of the reaction mixture. [c]Molecular weights ($M_{n,SEC}$)and dispersities (*Đ*)were determined by size-exclusion chromatography.

Table S7. Investigation of reaction conditions for the polymerization of M5[9][a]

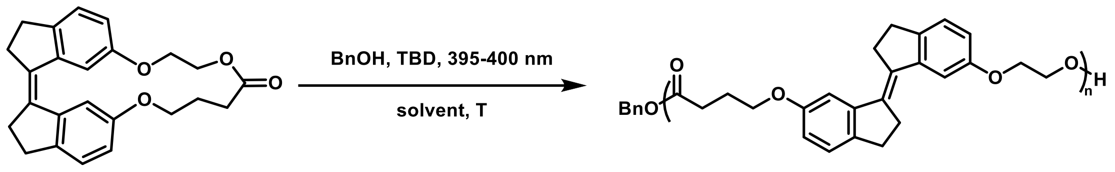

M5[9]

P5

entry	solvent	conc. (M)	temp. (°C)	time (h)	conv. (%)[b]	$M_{n,SEC}$ (kDa)[c]	*Đ*[c]
1	DCM	0.67	rt	16	19	-	-
2[d]	toluene	0.1	100	21	<2	-	-
3	toluene	0.1	100	21	34	-	-
4	toluene	0.5	100	6	63	3.4	2.99
5	toluene	1	100	6	73	3.1	1.93
6	o-xylene	0.5	100	6	82	2.6	2.75
7	DCE	0.5	100	6	10	-	-
8[e]	toluene	1	150	24	71	2.1	2.30
9[d,e]	toluene	1	150	24	18	-	-

[a]$[M]_0/[I]_0/[catalyst]_0$ = 10/1/1. [b]Conversions were determined by ^{1}H NMR spectroscopic analysis of the reaction mixture. [c]Molecular weights ($M_{n,SEC}$)and dispersities (*Đ*)were determined by size-exclusion chromatography. [d]Without light. [e]$Sn(Oct)_2$ as the catalyst.

5. General polymerization procedure and characterization of polymers

Preparation of stock solution: The desired amounts of BnOH and TBD were added into an oven-dried 2 mL vial under N_2. Dry, degassed DCM was then added to make a stock solution.

To an oven-dried microwave vial equipped with a magnetic stir bar was added the monomer (0.1 mmol). Following the evacuation and triple backfilling with N_2, the vial was charged with dry, degassed DCM, along with the initiator stock solution. After stirring for the indicated time, the reaction was quenched by three drops of trifluoracetic acid. An aliquot of the reaction mixture was taken for ^{1}H NMR to determine the conversion of the monomer. Another aliquot of the reaction mixture was taken for SEC analysis. The resulting polymer was precipitated from cold MeOH. The purified polymer was then characterized using SEC, ^{1}H-NMR, TGA and DSC.

In our photopolymerization setup, two blue LED lamps (Kessil PR160L, 390 nm) were positioned face-to face at a distance of 11.0 cm from each other, with the reaction vial placed at the center (Figure S9). According to the manufacturer's specifications, the light intensity at this position is approximately 150 mW/cm². To improve reproducibility and clarity, we have included both external and internal views of the photoreactor setup in the Supporting Information.

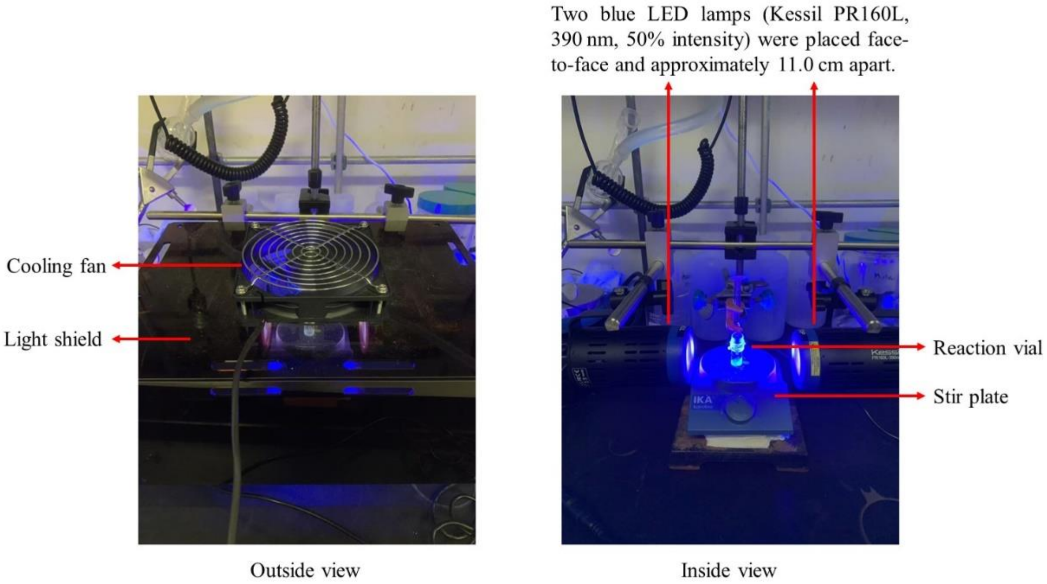

Figure S9. Schematic and actual setup of the photoirradiation system.

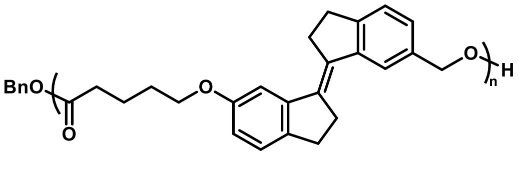

P3

^{1}H NMR (500 MHz, chloroform-*d*) δ 7.59 – 7.50 (m, 1H), 7.37 – 7.27 (m, 1H), 7.21 – 7.05 (comp, 3H), 6.78 – 6.66 (m, 1H), 5.17 – 5.06 (m, 2H), 4.08 – 3.85 (m, 2H), 3.24 – 2.89 (comp, 8H), 2.51 – 2.34 (m, 2H), 1.92 – 1.77 (comp, 4H).

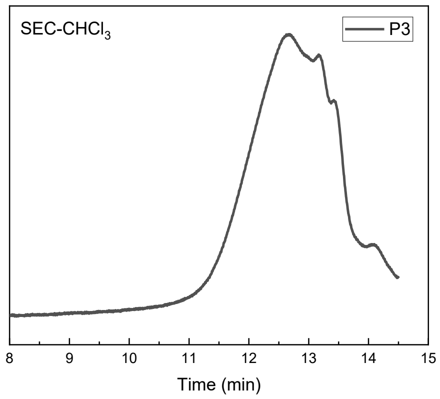

Figure S10. SEC trace of polymer P3.

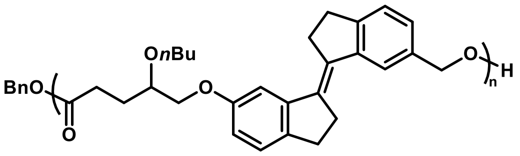

P4

^{1}H NMR (700 MHz, chloroform-*d*) δ 7.63 – 7.52 (m, 1H), 7.32 – 7.27 (m, 1H), 7.22 – 7.07 (comp, 3H), 6.80 – 6.71 (m, 1H), 5.15 (s, 2H), 4.02 (dd, *J* = 9.6, 5.2 Hz, 1H), 3.95 (dd, *J* = 9.6, 5.2 Hz, 1H), 3.71 – 3.64 (m, 2H), 3.51 – 3.44 (m, 1H), 3.17 – 3.10 (comp, 4H), 3.09 – 2.98 (comp, 4H), 2.64 – 2.47 (m, 2H), 2.11 – 2.05 (m, 1H), 2.00 – 1.94 (m, 1H), 1.59 – 1.51 (m, 2H), 1.41 – 1.32 (m, 2H), 0.90 (t, *J* = 7.4 Hz, 3H).

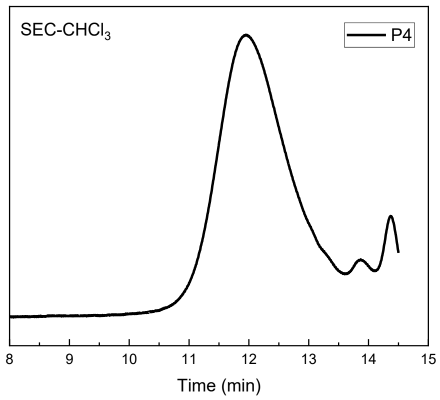

Figure S11. SEC trace of polymer P4.

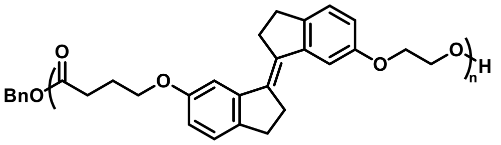

P5

^{1}H NMR (700 MHz, chloroform-*d*) δ 7.21 – 7.01 (comp, 4H), 6.80 – 6.67 (comp, 2H), 4.53 – 4.36 (m, 2H), 4.22 – 4.09 (m, 2H), 4.06 – 3.96 (m, 2H), 3.16 – 3.04 (comp, 4H), 3.02 – 2.86 (comp, 4H), 2.71 – 2.50 (m, 2H), 2.18 – 2.06 (m, 2H).

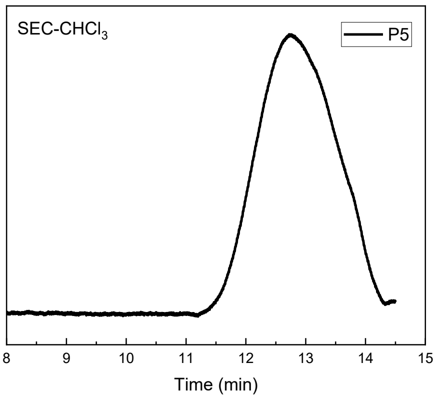

Figure S12. SEC trace of polymer P5.

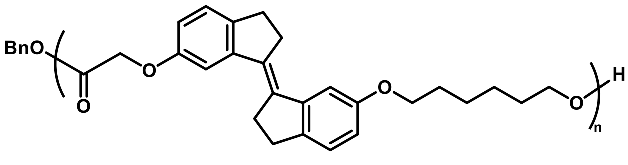

P6

^{1}H NMR (700 MHz, chloroform-*d*) δ 7.23 – 7.02 (comp, 4H), 6.79 – 6.69 (m, 2H), 4.66 – 4.55 (m, 2H), 4.27 – 4.13 (m, 2H), 4.00 – 3.85 (m, 2H), 3.21 – 3.02 (comp, 4H), 3.00 – 2.70 (comp, 4H), 1.81 – 1.62 (comp, 4H), 1.52 – 1.44 (m, 2H), 1.43 – 1.34 (m, 2H).

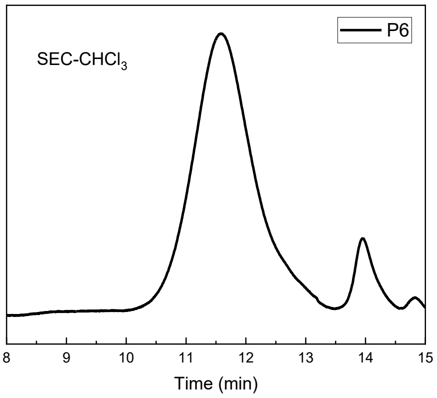

Figure S13. SEC trace of polymer P6.

Polymerization of *E*-M6[11] under dark conditions and *E*/*Z* ratio analysis of P6:

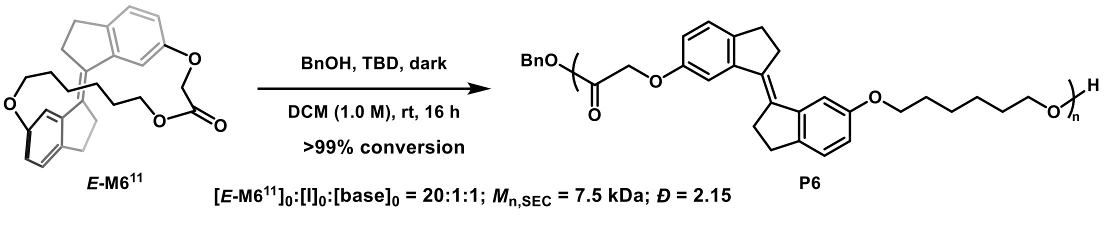

The polymerization of *E*-M6[11] was performed under dark conditions. Full monomer conversion was achieved, affording a polymer (P6) with an M_n of 7.5 kDa and a dispersity (*Đ*) of 2.15. Since this polymer was obtained from pure *E*-M6[11] in the absence of light, its structure should reflect a 100% *E*-configuration.

Then ^{1}H-NMR spectra of the two P6 samples−one from *E*-M6[11] (dark) and one from *Z*-M6[11] (390 nm)−were compared as shown in Figure S14. Aside from minor solvent residues, the spectra were

nearly identical, indicating that the P6 obtained from *Z*-M6[11]under light irradiation also adopts a 100% *E*-configuration.

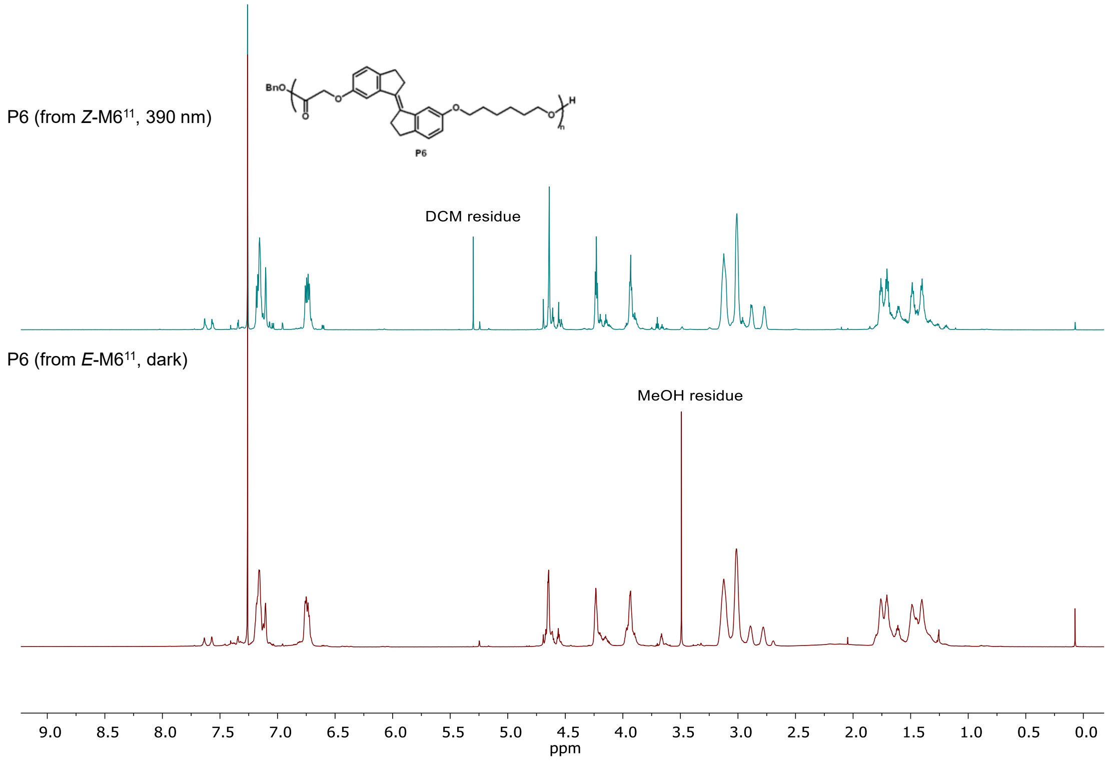

Figure S14. Overlay of ^{1}H-NMR spectra of P6 that were prepared from *Z*-M6[11] under 390 nm irradiation, and from *E*-M6[11] under dark conditions.

Table S8. Kinetic studies of the polymerization of *Z*-M6[11] under 390 nm irradiation[a]

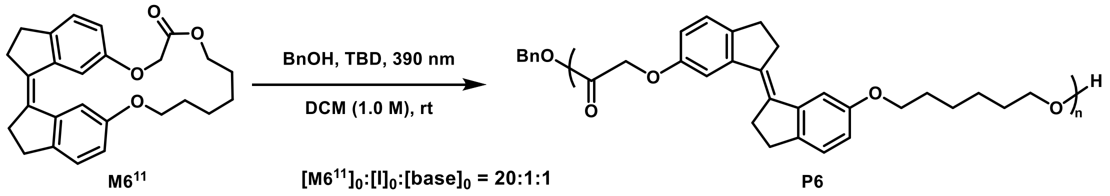

entry	time (h)	conv. (%)[b]	$M_{n,SEC}$ (kDa)[c]	$Đ$[c]
1	0.5	22	3.3	1.76
2	1	49	5.5	1.85
3	2	80	7.6	2.22
4	4	85	7.9	2.16
5	16	>99	8.5	2.36

[a]$[M6^{11}]_0/[I]_0/[base]_0$ = 20/1/1/1. [b]Monomer conversion was determined by ^{1}H NMR spectroscopy in $CDCl_3$. [c]Molecular weights ($M_{n,SEC}$) and dispersities ($Đ$), determined by $CHCl_3$ size-exclusion chromatography (SEC) calibrated using polystyrene standards.

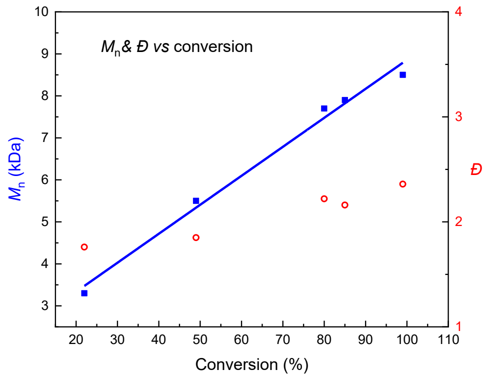

Figure S15. M_n–conversion correlation (blue) and $Đ$–conversion correlation (red) of polymerization of M6[11].

Copolymerization of M4[8] with caprolactone (CL) and *E*/*Z* ratio analysis of copolymers:

Preparation of stock solution: The desired amounts of BnOH and TBD were added into an oven-dried 2 mL vial under N_2. Dry, degassed DCM was then added to make a stock solution.

To an oven-dried microwave vial equipped with a magnetic stir bar was added the monomer M4 (0.1 mmol). Following the evacuation and triple backfilling with N_2, the vial was charged with dry, degassed DCM, CL (0.1 – 1.0 mmol), along with the initiator stock solution. After stirring for the indicated time, the reaction was quenched by three drops of trifluoracetic acid. An aliquot of the reaction mixture was taken for ^{1}H NMR to determine the conversion of the monomer. Another aliquot of the reaction mixture was taken for SEC analysis. The resulting polymer was precipitated from cold MeOH. The purified polymer was then characterized using SEC, ^{1}H-NMR, TGA and DSC.

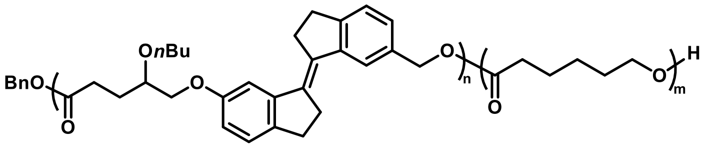

P4-co-CL

^{1}H NMR (500 MHz, chloroform-*d*) δ 7.60 – 7.48 (m, 1H), 7.38 – 7.28 (m, 1H), 7.23 – 7.03 (comp, 3H), 6.80 – 6.68 (m, 1H), 5.23 – 4.95 (m, 2H), 4.09 – 3.90 (comp, 4H), 3.72 – 3.57 (m, 2H), 3.53 – 3.40 (m, 1H), 3.25 – 3.03 (comp, 4H), 3.02 – 2.69 (comp, 4H), 2.61 – 2.40 (m, 2H), 2.33 – 2.24 (m, 2H), 2.12 – 1.81 (m, 2H), 1.67 – 1.50 (comp, 6H), 1.42 – 1.31 (comp, 4H), 0.99 – 0.82 (m, 3H).

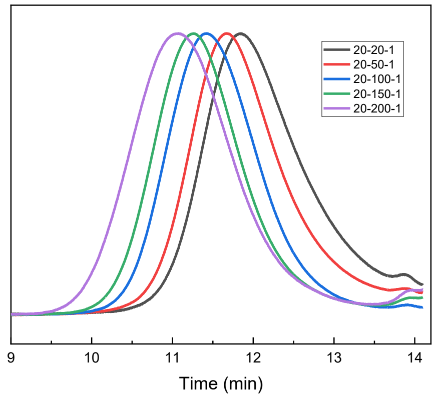

Figure S16. SEC trace of copolymers produced with varying $[M4^8]_0$: $[CL]_0$: $[BnOH]_0$ ratios.

The copolymerization of M4[8] and caprolactone (CL) was conducted under dark conditions. The monomer M4[8] was successfully incoporated into the copolymer, although its conversion was limited to 30%, while CL achieved full conversion (Table S9. entry 2). The resulting copolymer contained 23.6 mol% of M4[8], with an M_n of 4.7 kDa, a dispersity ($Đ$) of 1.58 and a glass transition temperature (T_g) of -27°C. The relatively low T_g may be attributed to both the stereochemistry and molecular weight of the copolymer. As this polymerization was performed in the absence of light, the incorporated M4[8] units are expected to retain 100% *Z*-configuration.

By comparing the ^{1}H-NMR spectra of P4-r-CL obtained under 390 nm irradiation and dark conditions, the *E*/*Z* ratio of the sample prepared under light was determined to be 4.1:1 (Figure S17). The *E*/*Z* ratios of other samples prepared with varying initial $[M4^8]_0$:$[CL]_0$:$[I]_0$ ratios have been updated accordingly (Table S9).

Table S9. Copolymerization of M4[8] with caprolactone (CL)[a]

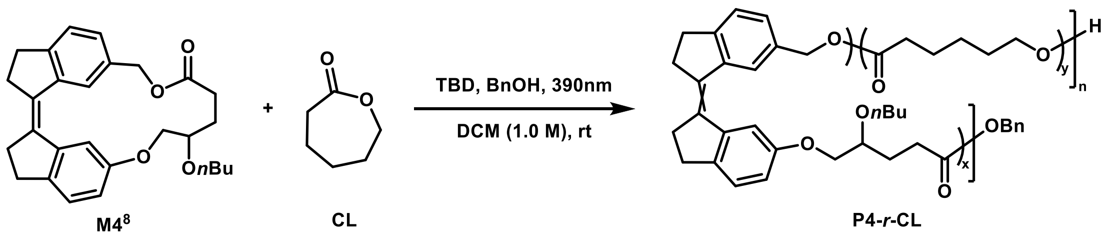

entry[a]	$[M4^8]_0$:$[CL]_0$:$[I]_0$	conv. (M4[8], %)[b]	conv. (CL%)[b]	M4[8] (%)[b]	*E*/*Z*[b]	$M_{n,SEC}$ (kDa)[c]	$Đ$[c]
1	20:20:1	87	99	41.6	4.1/1	8.2	1.98
2[d]	20:20:1	30	99	23.6	0/1	4.7	1.58
3	20:50:1	86	99	24.2	2.6/1	10.5	2.01
4	20:100:1	70	99	11.0	1/1	17.2	1.84
5	20:150:1	62	99	6.9	1/1.1	21.4	2.05
6	20:200:1	60	99	5.1	1/1.3	27.1	2.09

[a]$[M4^8]_0/[CL]_0/[I]_0/[base]_0$ = 20/1/1/1. [b]Monomer conversion, M4[8] incorporation and the *E*/*Z* ratios of the copolymer were determined by ^{1}H NMR spectroscopy in $CDCl_3$. [c]Molecular weights ($M_{n,SEC}$) and dispersities ($Đ$), determined by $CHCl_3$ size-exclusion chromatography (SEC) calibrated using polystyrene standards. [d]Without light.

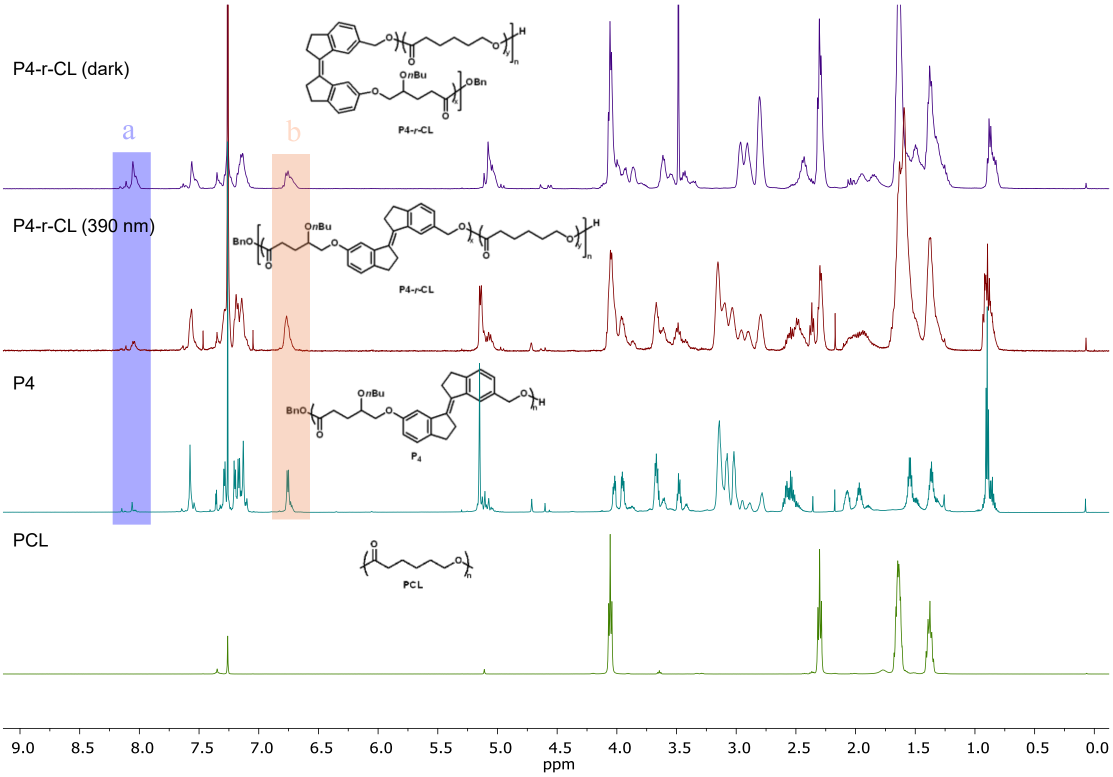

Figure S17. Overlay of ^{1}H-NMR spectra of P4-r-CL prepared from under dark conditions and under 390 nm irradiation, along with spectra of P4 and PCL for comparison.

6. Characterizing polymer chain-ends using MALDI-TOF

Polymerization of P6 targeting DP20:

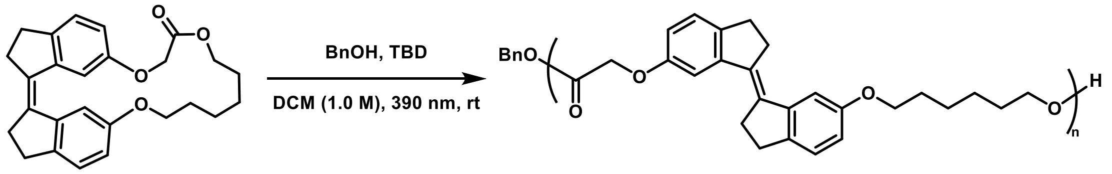

M6[11] **P6**

Preparation of stock solution: BnOH (10.81 mg, 10.35 µL, 0.1 mmol) and TBD (13.92 mg, 0.1 mmol) were added into an oven-dried 2 mL vial under N_2. Then dry DCM (1989.65 µL) was added to make a stock solution.

To an oven-dried microwave vial equipped with a magnetic stir bar was added the **M6**[11] monomer (40.5 mg, 0.1 mmol). After evacuation and backfilling with N_2 three times, dry DCM (30 µL) was added followed by the addition of initiator stock solution (40 µL). The total volume is around 100 µL and the initiating concentration of the monomer [**M6**[11]]$_0$ is around 1.0 M. After stirring for 16 h, the reaction was quenched with 3 drops of TFA. A sample of the crude material was taken for ^{1}H NMR to determine the conversion of the monomer. Another small amount of the crude material was taken for SEC analysis. The product was purified by precipitating from cold methanol (10 mL), then analyzed by matrix-assisted laser desorption/ionization-time-of-flight (MALDI-TOF) mass spectrometry.

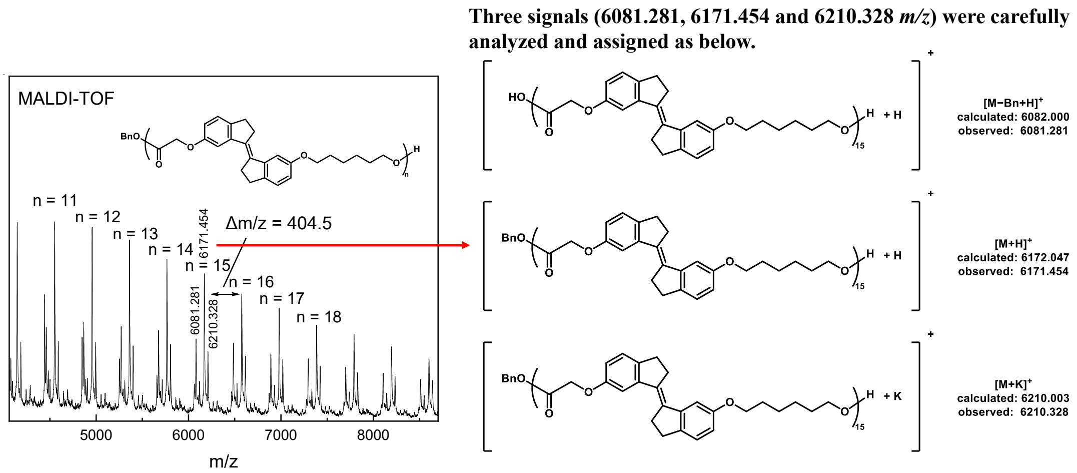

Figure S18. MALDI-TOF of P6 and detailed analysis of the ion peaks corresponding to 15 repeat units.

7. TGA and DSC studies of (co)polymers

TGA of purified (co)polymers were obtained in a nitrogen atmosphere at a heating rate of 10 °C/min. The $T_{d,5\%}$ (temperature causing a 5% weight loss) of each polymer was listed in Figure S19a. DSC analysis of purified (co)polymers were performed (-70 °C to 250 °C, heating rate: 10 °C/min, cooling rate: 10 °C/min). The glass transition temperature (T_g) of each polymer was listed in Figure S19b-f.

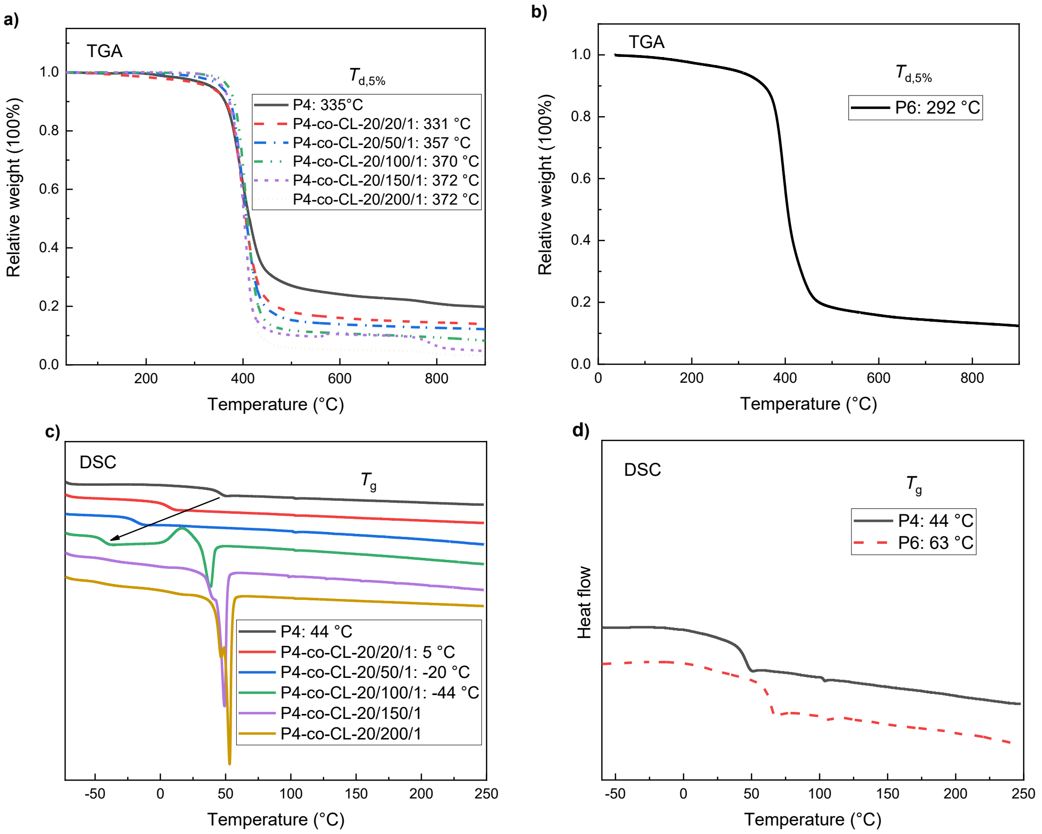

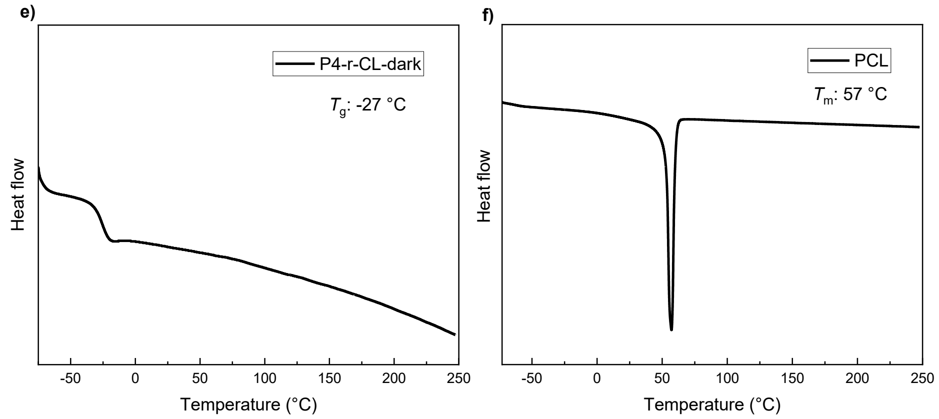

Figure S19. Thermal properties of (co)polymers. a, TGA curves of P4 and P4-co-CL. b, TGA curve of P6. c, DSC curves of P4 and P4-co-CL. d, DSC curve of P4 and P6. e, DSC curve of P4-r-CL that was prepared under dark conditions. e, DSC curve of polycaprolactone (PCL).

8. Chemical recycling of phoswitchable polymers

Preparation of ring-opening exchange product ROEM4[8]:

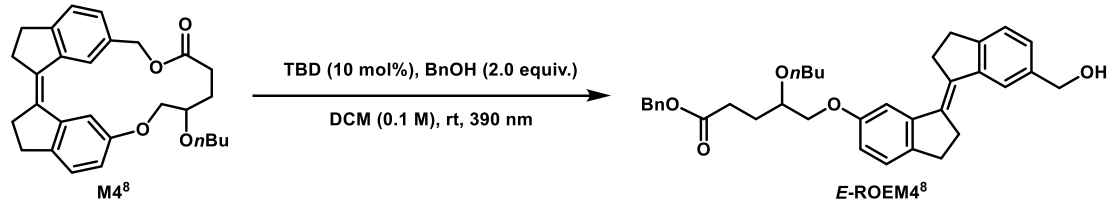

To an oven-dried microwave vial equipped with a magnetic stir bar was added the monomer **M4[8]** (0.1 mmol) and TBD (10 mol%). After evacuation and backfilling with N_2 three times, dry DCM (1.0 mL) was added, followed by the addition of BnOH (0.2 mmol, 2.0 equiv.). The reaction mixture was stirred at rt under 390 nm for 4 h. Upon completion, the reaction was quenched with 3 drops of TFA, concentrated under reduced pressure, and the residue was purified by column chromatography on silica gel (eluent: hexanes/ ethyl acetate = 4:1) to give the desired product (30.5 mg, 56% yield).

Benzyl (*E*)-4-butoxy-5-((6'-(hydroxymethyl)-2,2',3,3'-tetrahydro-[1,1'-biindenylidene]-6-yl)oxy)pentanoate.

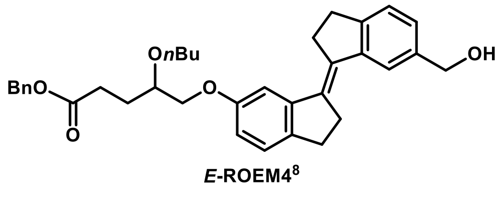

^{1}**H NMR** (700 MHz, chloroform-*d*) δ 7.68 – 7.57 (m, 1H), 7.39 – 7.35 (comp, 4H), 7.35 – 7.29 (comp, 2H), 7.23 – 7.18 (comp, 2H), 7.16 (d, *J* = 2.3 Hz, 1H), 6.78 (dd, *J* = 8.2, 2.4 Hz, 1H), 5.15 – 5.10 (m, 2H), 4.73 (s, 2H), 4.03 (dd, *J* = 9.6, 5.2 Hz, 1H), 3.96 (dd, *J* = 9.6, 5.2 Hz, 1H), 3.71 – 3.62 (m, 2H), 3.48 (dt, *J* = 9.3, 6.7 Hz, 1H), 3.21 – 3.15 (comp, 4H), 3.11 (dd, *J* = 8.7, 4.6 Hz, 2H), 3.07 – 3.00 (m, 2H), 2.63 – 2.50 (m, 2H), 2.07 (dddd, *J* = 14.3, 8.4, 7.0, 4.2 Hz, 1H), 1.96 (dtd, *J* = 14.3, 8.4, 6.1 Hz, 1H), 1.62 – 1.48 (comp, 2H), 1.40 – 1.31 (m, 2H), 0.91 (t, *J* = 7.4 Hz, 3H). ^{13}**C NMR** (176 MHz, chloroform-*d*) δ 173.5, 157.9, 147.0, 144.5, 143.8, 139.8, 139.2, 136.1, 136.0, 135.5, 128.7, 128.37, 128.36, 126.2, 125.4, 125.2, 123.5, 113.7, 111.1, 70.4, 70.3, 66.4, 66.0, 32.8, 32.3, 32.2, 31.0, 30.4, 30.3, 27.5, 19.5, 14.1. Peak overlapping was observed. **MS (m/z)**: calcd for $C_{13}H_{14}ClOS$, $[M+H]^+$: 253.04; found, 253.1.

E/Z photoisomerization of ROEM4[8]:

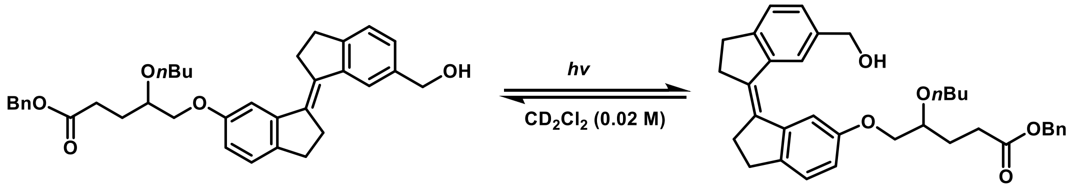

E-ROEM4[8] *Z*-ROEM4[8]

Benzyl (*Z*)-4-butoxy-5-((6'-(hydroxymethyl)-2,2',3,3'-tetrahydro-[1,1'-biindenylidene]-6-yl)oxy)pentanoate

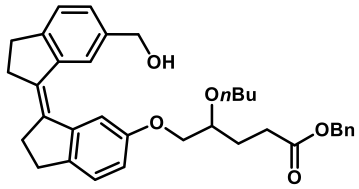

^{1}H NMR (700 MHz, chloroform-*d*) δ 8.16 (d, *J* = 1.5 Hz, 1H), 7.65 (d, *J* = 2.4 Hz, 1H), 7.39 – 7.30 (comp, 5H), 7.27 (s, 1H), 7.17 (d, *J* = 8.2 Hz, 1H), 7.13 (dd, *J* = 7.7, 1.5 Hz, 1H), 6.76 (dd, *J* = 8.2, 2.4 Hz, 1H), 5.14 – 5.03 (m, 2H), 4.62 (s, 2H), 4.00 (dd, *J* = 9.7, 5.2 Hz, 1H), 3.92 (dd, *J* = 9.7, 5.2 Hz, 1H), 3.64 – 3.57 (m, 2H), 3.44 (dt, *J* = 9.3, 6.7 Hz, 1H), 2.97 (dd, *J* = 8.2, 5.4 Hz, 2H), 2.93 (dd, *J* = 8.2, 5.0 Hz, 2H), 2.85 – 2.78 (comp, 4H), 2.59 – 2.40 (m, 2H), 2.03 (dtd, *J* = 15.0, 7.5, 4.4 Hz, 1H), 1.92 (dtd, *J* = 14.3, 7.9, 6.4 Hz, 1H), 1.54 – 1.46 (m, 2H), 1.37 – 1.29 (m, 2H), 0.88 (t, *J* = 7.4 Hz, 3H). **^{13}C NMR** (176 MHz, chloroform-*d*) δ 173.9, 157.3, 147.9, 141.7, 141.0, 140.9, 138.7, 136.0, 135.7, 135.0, 128.7, 128.41, 128.37, 126.4, 125.7, 125.3, 122.4, 115.7, 108.5, 77.0, 70.3, 70.2, 66.5, 65.4, 35.4, 35.1, 32.2, 30.6, 30.2, 30.0, 27.3, 19.4, 14.0. **MS (m/z)**: calcd for $C_{13}H_{14}ClOS$, $[M+H]^+$: 253.04; found, 253.1.

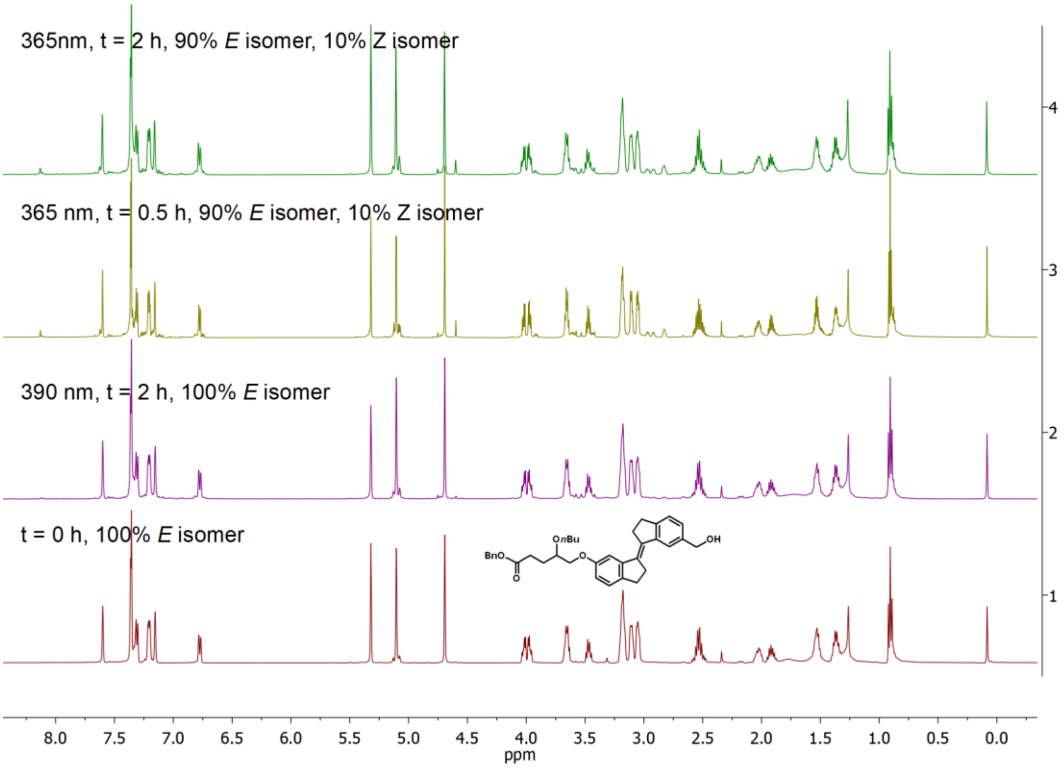

Figure S20. Time-dependent ^{1}H-NMR spectra of **ROEM4[8]** under 390/365 nm irradiation in CD_2Cl_2 (0.02 M).

Determination of the E/Z ratio in polymer P4:

This analysis was carried out by overlaying the ^{1}H-NMR spectra of *Z*-ROM4[8], *E*-ROM4[8] and P4 (Figure S21). The *E*/*Z* ratio in P4 was then determined by integrating the relative intensities of the highlighted peaks according to the given equation.

***E/Z* ratio Calculation of P4 (Table 1, entry 4):**

$$E:Z = \frac{Integration\ (7.76ppm-7.46ppm) - Integration\ (8.20\ ppm-7.90\ ppm)}{Integration\ (8.20\ ppm-7.90\ ppm)} = 4.6:1$$

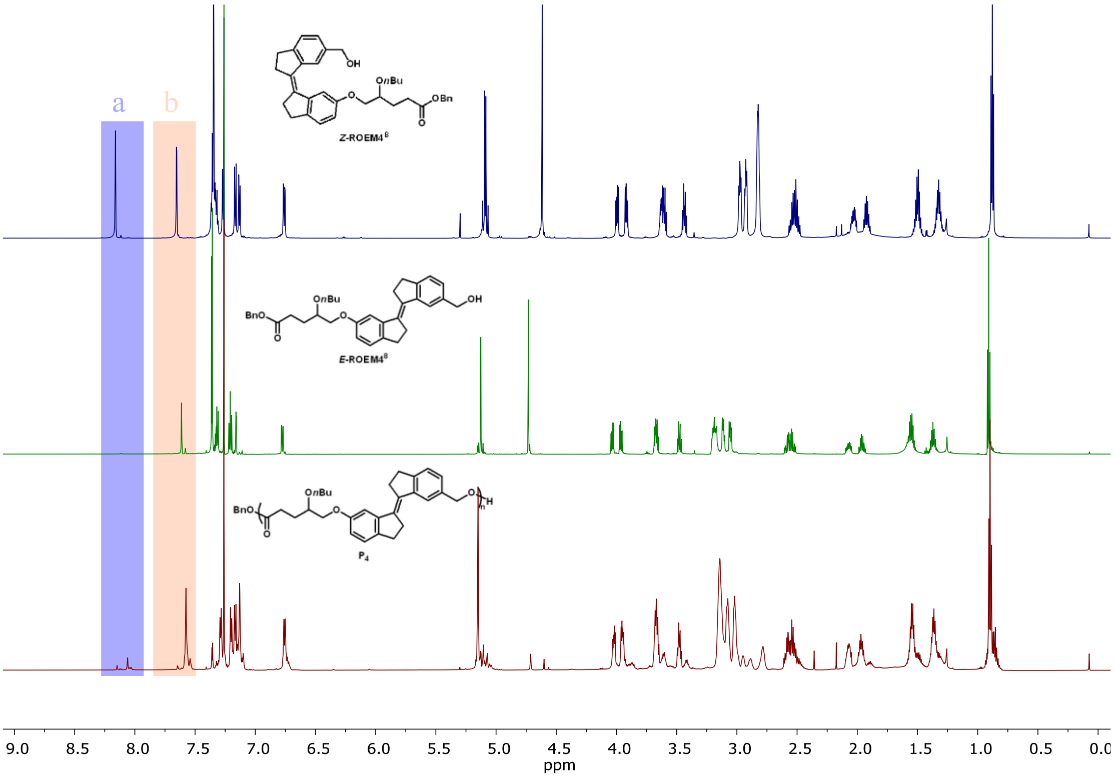

Figure S21. Overlay of ^{1}H-NMR spectra of *Z*-ROM4[8], *E*-ROM4[8] and P4.

Depolymerization of P4:

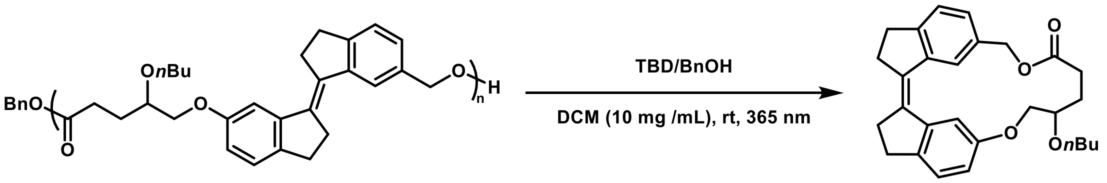

P4 (DP: 20, M_n: 6.9 kDa, *Đ*: 2.03) M4[8]

To a solution of P4 (DP20, 4.41 mg, 7.35×10^{-4} mmol, 1.0 equiv.; repeat unit: 1.0 × 10^{-2} mmol) in DCM was added the stock solution of TBD and BnOH in DCM. After stirring for 18 h under the 365 nm, the reaction was quenched by TFA. The solvent was removed by evaporation and the residue was analyzed by ^{1}H NMR spectroscopy and SEC. The crude mixture from the conditions of 0.75 equiv. TBD and BnOH was then purified by column chromatography on silica gel (eluent: hexanes/ethyl acetate = 10:1) to give product **M4[8]** (43% yield, 1.92 mg).

Table S10. Investigation of the amount of TBD and BnOH for the depolymerization[a]

entry	TBD	BnOH	conversion (%)[b]
1	0.1 equiv.	0.1 equiv.	8
2	0.2 equiv.	0.2 equiv.	13
3	0.5 equiv.	0.5 equiv.	43
4	0.75 equiv.	0.75 equiv.	46 (43)[c]
5[d]	0.75 equiv.	0.75 equiv.	21
6	1.0 equiv.	1.0 equiv.	46

[a]The amount of TBD and BnOH was relative to the repeat unit. [b]The conversion was determined by ^{1}H NMR spectroscopic analysis of the reaction mixture. [c]Isolated yield of recovered monomer in parentheses. [d]Without light.

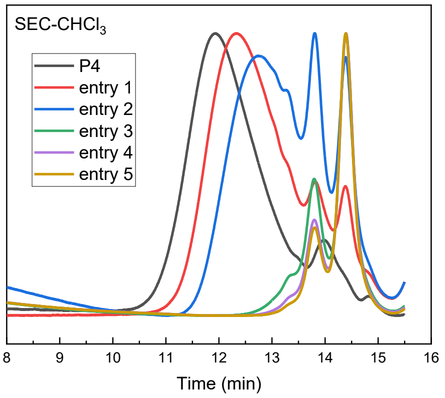

Figure S22. SEC curves for depolymerization of P4 under the conditions in Table S8.

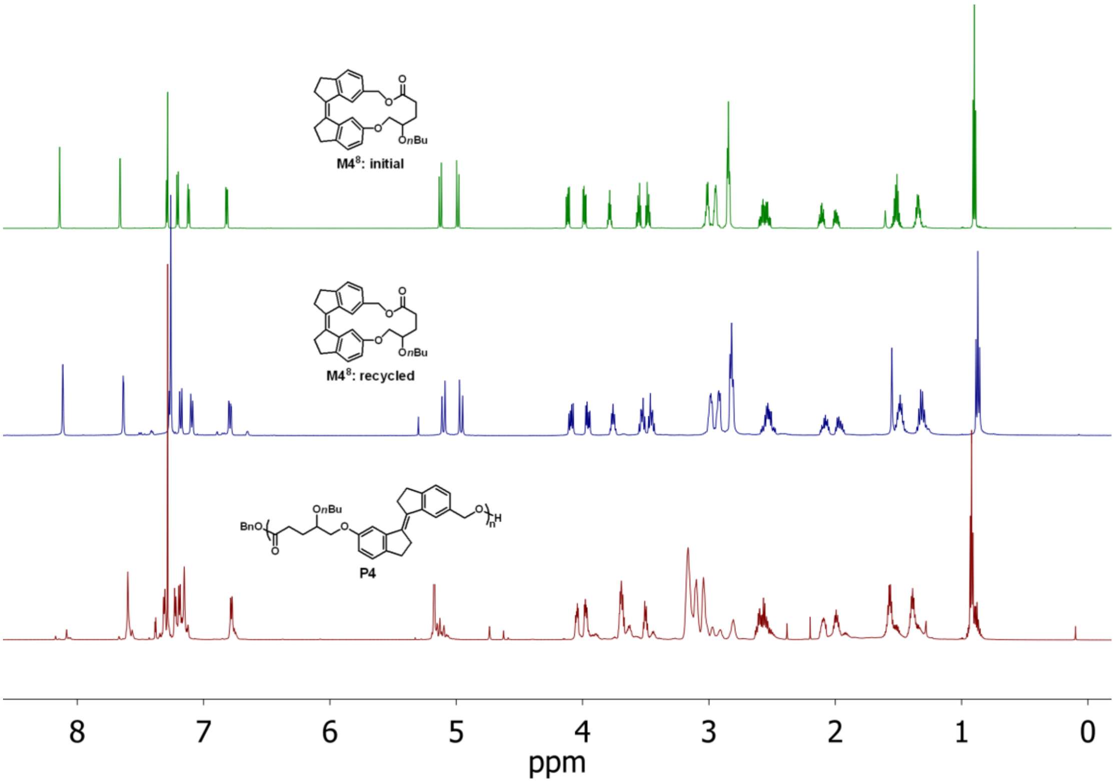

Figure S23. Overlay of ^{1}H-NMR spectra of initial **M4^8**, recycled **M4^8** and **P4**.

Depolymerization of P6:

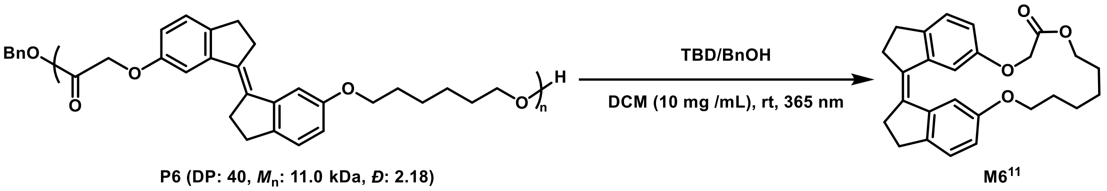

Table S11. Investigation of the amount of TBD and BnOH for the depolymerization of P6[a]

entry	TBD	BnOH	conversion (%)[b]
1	0.5 equiv.	0.5 equiv.	9 (1)
2	0.75 equiv.	0.75 equiv.	16 (3)
3	1.0 equiv.	1.0 equiv.	31 (6)
4[c]	1.0 equiv.	1.0 equiv.	<2

[a]The amount of TBD and BnOH was relative to the repeat unit. [b]The conversion was determined by ^{1}H NMR spectroscopic analysis of the reaction mixture; values in parentheses indicate the amount of *E*-M6[11]. [c]Without light.

9. Polarized optical microscopy (POM) of P4 and P6

Polarized optical microscopy (POM) was completed using a Nikon Eclipse E400 POL with a mounted Qimaging MicroPublisher 3.3 RTV digital lens. The heating microscope stage used was a Linkam LTS 350 supplied by a Linkam CI94 temperature controller and Linksys32 software.

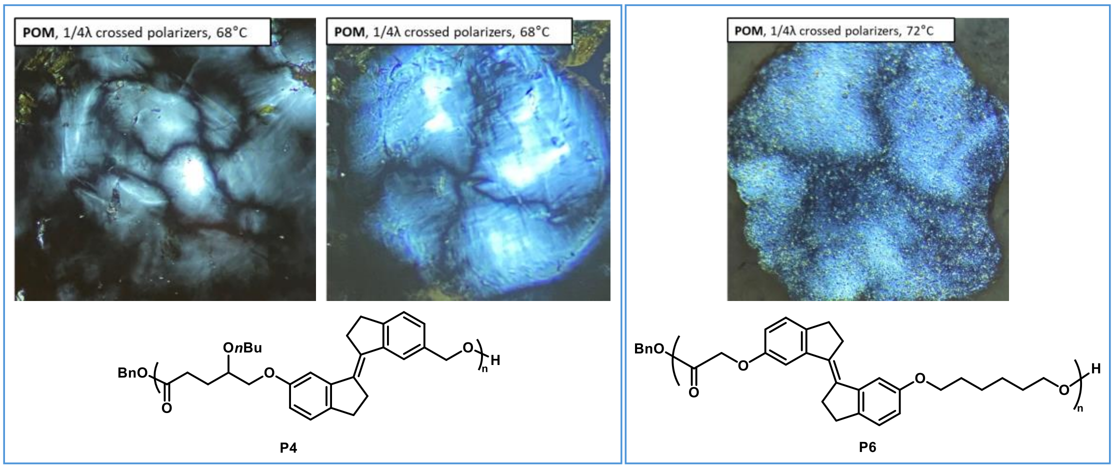

Figure S24. The POM of **P4** and **P6**.

10. X-ray crystal structure of the monomer $\mathbf{M3^{8}}$ and $\mathbf{M6^{11}}$

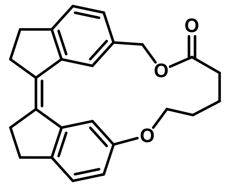

$M3^{8}$

≡

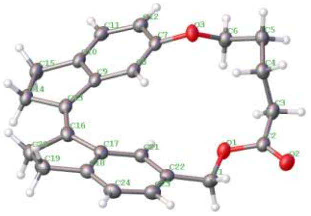

X-Ray of $M3^{8}$; CCDC: 2314748

Experimental. Single crystals of $C_{24}H_{24}O_3$ $\mathbf{M3^{8}}$ were prepared by slow evaporation of hexane/ethyl acetate solution. Single colorless triclinic-shaped crystals of $\mathbf{M3^{8}}$ were chosen from the sample. A suitable crystal was selected and mounted on a loop with paratone on a XtaLAB Synergy-S diffractometer. The crystal was kept at a steady T = 100.00 K during data collection. Using Olex 2,[3] The structure was solved with the SHELXT[4] structure solution program using Intrinsic Phasing and refined with the SHELXL[5]refinement package using Least Squares minimization.

Crystal Data. $C_{24}H_{24}O_3$, M_r = 360.43, triclinic, P-1 (No. 2), a = 9.1188(19) Å, b = 10.524(2) Å, c = 11.670(3) Å, α = 114.779(8)°, β = 93.499(12)°, γ = 113.152(10)°, V = 900.0(4) Å^3, T = 100.00 K, Z = 2, μ (Mo K_{α}) = 0.086 mm^{-1}, $Dcalc$ = 1.330g/cm^3, 28636 reflections measured, 3724 unique (R_{int} = 0.0971, R_{sigma} = 0.0548) which were used in all calculations. The final wR_2 was 0.1349 (all data) and R_1 was 0.0598 (I>2 σ(I)).

Table S12: Crystallographic data and structure refinement for **M3**[8].

**Compound**	**M3**[8]
Formula	$C_{24}H_{24}O_3$
$D_{calc.}$/ g cm^{-3}	1.330
μ/mm^{-1}	0.086
Formula Weight	360.43
Color	colorless
Size/mm^3	0.312×0.138×0.123
T/K	100.00
Crystal System	triclinic
Space Group	*P*-1
a/Å	9.1188(19)
b/Å	10.524(2)
c/Å	11.670(3)
α/°	114.779(8)
β/°	93.499(12)
γ/°	113.152(10)
V/Å^3	900.0(4)
Z	2
Wavelength/Å	0.71073
Radiation type	Mo K_a (λ= 0.71073)
2Θ/°	3.994 to 52.976
Measured Refl's.	28636
Indep't Refl's	3724
R_{int}	0.0971
Parameters	244
Restraints	0
Largest Peak	0.22
Deepest Hole	-0.23
GooF	1.030
wR_2 (all data)	0.1349
wR_2	0.1135
R_1 (all data)	0.1030
R_1	0.0598

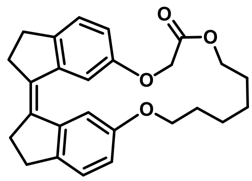

M6[11]

≡

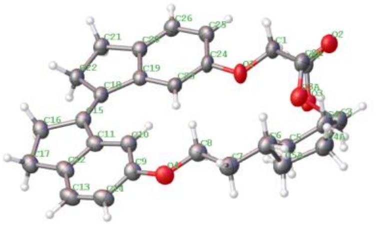

X-Ray of M6[11]; CCDC: 2402447

Experimental. Single crystals of $C_{26}H_{28}O_4$ **M6[11]** were prepared by slow evaporation of hexane/ethyl acetate solution. Single yellowish block-shaped crystal of **M6[11]** was mounted on a nylon loop with NVH oil. Data were collected from a shock-cooled single crystal at 100.00 K on a Bruker D8 VENTURE dual wavelength Mo/Cu three-circle diffractometer with a microfocus sealed X-ray tube using a mirror optics as monochromator and a Bruker PHOTON III detector. The diffractometer was equipped with an Oxford Cryostream 800 low temperature device and used MoK_{α} radiation (λ = 0.71073 Å). All data were integrated with SAINT and a multi-scan absorption correction using SADABS was applied.[6,7] The structure was solved by dual methods using SHELXT and refined by full-matrix least-squares methods against F^2 by SHELXL using Olex2.[3-5] All non-hydrogen atoms were refined with anisotropic displacement parameters. All hydrogen atoms were refined isotropic on calculated positions using a riding model with their U_{iso} values constrained to 1.5 times the U_{eq} of their pivot atoms for terminal sp^3 carbon atoms and 1.2 times for all other carbon atoms. Disordered moieties were refined using bond lengths restraints and displacement parameter restraints. Crystallographic data for the structures reported here have been deposited with the Cambridge Crystallographic Data Centre.[8] CCDC 2402477 contain the supplementary crystallographic data for this paper. These data can be obtained free of charge from The Cambridge Crystallographic Data Centre via www.ccdc.cam.ac.uk/structures. This report and the CIF file were generated using FinalCif.[9]

Crystal Data. $C_{26}H_{28}O_4$, M_r = 404.48, monoclinic, $P2_1/n$ (14), a = 4.9662(13) Å, b = 20.971(4) Å, c = 19.792(9) Å, α = 90°, β = 90.77(3)°, γ = 90°, V = 2061.1(12) Å^3, T = 100.00 K, Z = 4, μ (Mo K_{α}) = 0.087 mm^{-1}, 28656 reflections measured, 4070 unique (R_{int} = 0.0875) which were used in all calculations. The final wR_2 was 0.2115 (all data) and R_1 was 0.1320 ($I \geq 2\sigma(I)$).

Table S13: Crystallographic data and structure refinement for **M6**[11]

**Compound**	**M6**[11]
Formula	$C_{26}H_{28}O_4$
$D_{calc.}$/ g cm^{-3}	1.303
μ/mm^{-1}	0.087
Formula Weight	404.48
Color	yellow
Shape	block-shaped
Size/mm^3	0.19×0.14×0.1
T/K	100.00
Crystal System	monoclinic
Space Group	$P2_1/n$ (14)
a/Å	4.9662(13)
b/Å	20.971(4)
c/Å	19.792(9)
α/°	90
β/°	90.77(3)
γ/°	90
V/Å^3	2061.1(12)
Z	4
Wavelength/Å	0.71073
Radiation type	Mo K_a
2Θ/°	4.40 το 52.18 (0.81 Å)
Measured Refl's.	28656
Indep't Refl's	4070
R_{int}	0.0875
R_{sigma}	0.0570
Parameters	308
Restraints	36
Largest Peak	0.41
Deepest Hole	-0.42
GooF	1.078
wR_2 (all data)	0.2115
wR_2	0.1812
R_1 (all data)	0.1320
R_1	0.0826

11. Computational details

All Density Functional Theory (DFT) calculations were performed using the Gaussian 09 software package.[10] Geometry optimizations and frequency calculations for intermediates and transition states (TS) were carried out employing the (U)B3LYP functional with the 6-31G(d) basis set.[11] In addition, single-point energy calculations were conducted using the 6-31G(d,p) basis set and the Polarizable Continuum Model (PCM) with tetrahydrofuran (THF) as the solvent.[12] To investigate the photoisomerization mechanism, the process was studied at the CASSCF level using Gaussian 09. Initially, the geometries of stationary points and rotary reaction paths on both the S_0 and S_1 potential energy surfaces were optimized using Time-Dependent Density Functional Theory (TD-DFT). The energies were then further corrected with a state-averaged CASSCF (SA-CASSCF) wave function employing the 6-31G(d) basis set. The active space was defined as CAS(10,10), consisting of 10 electrons distributed over 10 frontier π orbitals.[13] Due to computational cost considerations, the rotary minimal-energy paths (MEPs) were not rigorously determined; instead, approximate energy profiles along the C1–C1' rotary coordinate on the S_0 and S_1 surfaces were obtained by performing a series of constrained geometry optimizations where the torsional angle of the ethylenic C=C bond was fixed while all other geometric parameters were relaxed.

Geometric coordinate

M3[8]

Standard orientation:

Center Number	Atomic Number	Atomic Type	Coordinates (Angstroms) X	Y	Z
1	6	0	-4.321107	0.008424	-0.381533
2	6	0	-2.835447	-0.265585	-0.144866
3	6	0	-2.186277	1.061021	-0.074969
4	6	0	-3.073872	2.044149	-0.560376
5	6	0	-4.362728	1.413539	-1.032385
6	6	0	-0.968963	1.454968	0.501102
7	6	0	-0.623851	2.809909	0.548063
8	6	0	-1.493567	3.768805	0.007831
9	6	0	-2.722897	3.390306	-0.536284
10	6	0	0.629220	3.284338	1.256057
11	8	0	2.530647	-1.591768	-0.926575
12	8	0	1.646577	2.261209	1.310681
13	6	0	1.422077	-2.195284	-0.388087
14	6	0	0.231064	-1.463373	-0.462556
15	6	0	-0.956407	-2.031212	0.007923
16	6	0	-0.950460	-3.350320	0.511235
17	6	0	0.241672	-4.058731	0.609071
18	6	0	1.438545	-3.482086	0.168283
19	6	0	-2.341337	-1.521986	-0.027304
20	6	0	-3.238275	-2.750571	0.124826

21	6	0	-2.353813	-3.791357	0.859270
22	6	0	3.805177	-1.822859	-0.312680
23	6	0	4.764433	-0.730373	-0.792944
24	6	0	4.153706	0.680219	-0.803522
25	6	0	3.575799	1.131552	0.546913
26	6	0	2.644466	2.315176	0.394647
27	8	0	2.758818	3.199267	-0.429854
28	1	0	-4.860426	0.029877	0.576172
29	1	0	-4.796545	-0.756986	-1.000246
30	1	0	-5.247206	1.992710	-0.749998
31	1	0	-4.372818	1.327579	-2.127330
32	1	0	-0.300487	0.723646	0.938065
33	1	0	-1.216965	4.819746	0.034490
34	1	0	-3.401339	4.145122	-0.925001
35	1	0	0.414511	3.517557	2.303705
36	1	0	1.035746	4.175162	0.773649
37	1	0	0.259251	-0.476484	-0.908338
38	1	0	0.251076	-5.069982	1.007449
39	1	0	2.364844	-4.042830	0.225400
40	1	0	-4.165346	-2.532078	0.661131
41	1	0	-3.520518	-3.140099	-0.863632
42	1	0	-2.564087	-4.821281	0.554519
43	1	0	-2.523675	-3.737920	1.943252
44	1	0	4.194729	-2.812549	-0.583914
45	1	0	3.687537	-1.800757	0.778374
46	1	0	5.096989	-0.964251	-1.810625
47	1	0	5.656922	-0.770599	-0.155752
48	1	0	4.907755	1.399445	-1.137729
49	1	0	3.355010	0.700469	-1.549321
50	1	0	4.377545	1.420845	1.239234
51	1	0	3.016225	0.331991	1.036144

##

E-M3[8]

Standard orientation:

Center Number	Atomic Number	Atomic Type	Coordinates (Angstroms) X	Y	Z
1	6	0	-0.703766	-2.031285	-2.104021
2	6	0	-0.623823	-2.030575	-0.577829
3	6	0	-1.947748	-1.581446	-0.125440

4	6	0	-2.857229	-1.581702	-1.202003
5	6	0	-2.218110	-2.275852	-2.385245
6	6	0	-2.179302	-0.765284	0.986366
7	6	0	-3.243614	0.138270	0.971182
8	6	0	-4.189769	0.071898	-0.062073
9	6	0	-4.016368	-0.812919	-1.135421
10	6	0	-3.164690	1.316486	1.919347
11	8	0	3.394525	1.377962	-1.368595
12	8	0	-1.811749	1.879402	1.864267
13	6	0	3.266088	0.170326	-0.707259
14	6	0	2.136225	-0.600959	-0.966655
15	6	0	1.836163	-1.670621	-0.112290
16	6	0	2.723776	-2.021231	0.925845
17	6	0	3.926608	-1.336821	1.077258
18	6	0	4.197046	-0.228243	0.264710
19	6	0	0.486317	-2.163945	0.192429
20	6	0	0.539367	-2.596247	1.658149
21	6	0	2.032996	-2.987281	1.863396
22	6	0	3.161206	2.551825	-0.547687
23	6	0	1.953097	3.364277	-1.026554
24	6	0	0.538280	2.799444	-0.790882
25	6	0	0.024098	2.841565	0.663074
26	6	0	-1.471303	2.546967	0.735204
27	8	0	-2.276794	2.873880	-0.113309
28	1	0	-0.047731	-2.777143	-2.561076
29	1	0	-0.434908	-1.050707	-2.521282
30	1	0	-2.432171	-3.352977	-2.385005
31	1	0	-2.544240	-1.874477	-3.348299
32	1	0	-1.428969	-0.679814	1.763885
33	1	0	-5.016975	0.776129	-0.078487
34	1	0	-4.722674	-0.801850	-1.961228
35	1	0	-3.277618	1.037472	2.969012
36	1	0	-3.894506	2.086873	1.664334
37	1	0	1.433283	-0.255458	-1.714994
38	1	0	4.618734	-1.601618	1.872295
39	1	0	5.087996	0.370777	0.424922
40	1	0	0.314258	-1.759169	2.333608
41	1	0	-0.161912	-3.403824	1.885689
42	1	0	2.194825	-4.030639	1.561097
43	1	0	2.360614	-2.890584	2.902015
44	1	0	4.058189	3.176893	-0.623113
45	1	0	3.055131	2.247809	0.499237
46	1	0	2.081411	3.532792	-2.102532
47	1	0	2.028072	4.354008	-0.554669

48	1	0	-0.161380	3.380669	-1.399286
49	1	0	0.480037	1.771674	-1.163878
50	1	0	0.172316	3.847186	1.080605
51	1	0	0.557089	2.146147	1.316634

###

TS-M3[8]

Standard orientation:

Center Number	Atomic Number	Atomic Type	Coordinates (Angstroms) X	Y	Z
1	6	0	2.039399	2.465284	-1.885820
2	6	0	1.538972	2.061232	-0.508686
3	6	0	2.262784	0.909806	-0.081164
4	6	0	3.291681	0.591591	-1.012986
5	6	0	3.367601	1.672709	-2.068353
6	6	0	1.944544	0.004213	0.952054
7	6	0	2.596764	-1.227955	1.012098
8	6	0	3.627625	-1.518705	0.103249
9	6	0	3.986321	-0.601228	-0.900557
10	6	0	2.091314	-2.274921	1.979916
11	8	0	-3.128478	-0.422926	-1.477749
12	8	0	0.648802	-2.429806	1.832642
13	6	0	-2.748154	0.669486	-0.731699
14	6	0	-1.442606	1.125824	-0.894177
15	6	0	-0.978619	2.172571	-0.068393
16	6	0	-1.855362	2.784071	0.874180
17	6	0	-3.168200	2.355200	0.977368
18	6	0	-3.624577	1.295308	0.175061
19	6	0	0.363935	2.595293	0.158940
20	6	0	0.398074	3.462216	1.407416
21	6	0	-1.093405	3.801946	1.693024
22	6	0	-3.784004	-1.500978	-0.779988
23	6	0	-3.160065	-2.830748	-1.202628
24	6	0	-1.656984	-2.969872	-0.914939
25	6	0	-1.282893	-3.020650	0.576555
26	6	0	0.219935	-3.137135	0.761773
27	8	0	0.959620	-3.776565	0.040136
28	1	0	2.178716	3.548813	-1.973691
29	1	0	1.308794	2.188472	-2.661606
30	1	0	4.228075	2.330138	-1.887095

31	1	0	3.487130	1.267828	-3.077910
32	1	0	1.123414	0.217857	1.629494
33	1	0	4.121174	-2.485144	0.147961
34	1	0	4.765238	-0.859791	-1.613232
35	1	0	2.212405	-1.971215	3.022087
36	1	0	2.582175	-3.236588	1.821517
37	1	0	-0.778040	0.606683	-1.576605
38	1	0	-3.845272	2.810144	1.695973
39	1	0	-4.645714	0.941089	0.268104
40	1	0	0.843599	2.910634	2.249836
41	1	0	1.017163	4.356447	1.270896
42	1	0	-1.319065	4.822281	1.355879
43	1	0	-1.342307	3.759821	2.758008
44	1	0	-4.852647	-1.492612	-1.027663
45	1	0	-3.691786	-1.345758	0.300382
46	1	0	-3.321060	-2.959309	-2.279450
47	1	0	-3.721901	-3.633541	-0.706328
48	1	0	-1.283735	-3.880424	-1.393095
49	1	0	-1.130175	-2.135066	-1.389213
50	1	0	-1.747715	-3.894580	1.052681
51	1	0	-1.632493	-2.139153	1.119385

##

M6[11]

Standard orientation:

Center Number	Atomic Number	Atomic Type	Coordinates (Angstroms) X	Y	Z
1	6	0	-3.651758	-2.755134	0.689994
2	6	0	-2.776179	-1.568317	0.273133
3	6	0	-1.389425	-2.079272	0.232405
4	6	0	-1.347521	-3.361774	0.829955
5	6	0	-2.694350	-3.745341	1.388869
6	6	0	-0.242722	-1.574486	-0.390579
7	6	0	0.932964	-2.343092	-0.409936
8	6	0	0.969331	-3.607159	0.196290
9	6	0	-0.179150	-4.108233	0.817229
10	8	0	2.000020	-1.783763	-1.061448
11	8	0	-0.223539	3.606193	0.225558
12	6	0	-1.372967	2.933256	-0.142804
13	6	0	-1.556100	1.561367	0.067230

14	6	0	-2.800807	0.989755	-0.255273
15	6	0	-3.833504	1.812953	-0.759819
16	6	0	-3.629584	3.171601	-0.973237
17	6	0	-2.392083	3.735017	-0.669335
18	6	0	-3.341096	-0.366537	-0.010053
19	6	0	-4.864367	-0.234976	-0.105794
20	6	0	-5.106142	1.029466	-0.957548
21	6	0	1.033954	3.124816	-0.208834
22	6	0	1.836910	2.520159	0.935923
23	8	0	3.147982	2.716125	0.711489
24	6	0	3.174560	-2.583394	-1.262040
25	6	0	5.042172	-0.819355	-0.993337
26	6	0	4.183707	0.159272	-0.185115
27	8	0	1.359772	1.926055	1.879252
28	6	0	4.242384	-1.729661	-1.939964
29	1	0	-4.092180	-3.228217	-0.199308
30	1	0	-4.483139	-2.452863	1.332194
31	1	0	-2.950771	-4.792988	1.202386
32	1	0	-2.720711	-3.599842	2.477374
33	1	0	-0.240822	-0.620640	-0.904544
34	1	0	1.868331	-4.209393	0.175737
35	1	0	-0.152219	-5.095523	1.271013
36	1	0	-0.767895	0.986638	0.539425
37	1	0	-4.430871	3.795240	-1.360346
38	1	0	-2.200140	4.793304	-0.811004
39	1	0	-5.334512	-1.124161	-0.533221
40	1	0	-5.294581	-0.095551	0.896259
41	1	0	-5.998933	1.587055	-0.658263
42	1	0	-5.236441	0.760332	-2.014643
43	1	0	1.593604	3.961868	-0.636085
44	1	0	0.926317	2.357045	-0.985855
45	1	0	2.913221	-3.450706	-1.883010
46	1	0	3.546921	-2.959002	-0.299247
47	1	0	5.626436	-1.445248	-0.304239
48	1	0	5.774549	-0.257881	-1.588622
49	1	0	3.535804	0.716606	-0.871128
50	1	0	3.511543	-0.399344	0.476320
51	1	0	3.758849	-1.136513	-2.725915
52	1	0	4.937556	-2.409766	-2.446517
53	6	0	4.112636	2.014806	1.544948
54	1	0	3.566429	1.415614	2.275997
55	1	0	4.686109	2.783669	2.069087
56	6	0	4.996560	1.154393	0.649754
57	1	0	5.595350	1.798746	-0.006085

58	1	0	5.703786	0.623867	1.301073

##

E-M6[11]

Standard orientation:

Center Number	Atomic Number	Atomic Type	Coordinates (Angstroms) X	Y	Z
1	6	0	0.066763	-1.671183	2.216665
2	6	0	0.445694	-1.867583	0.750182
3	6	0	1.897486	-1.651510	0.682294
4	6	0	2.442341	-1.610423	1.981343
5	6	0	1.378297	-1.974873	2.992553
6	6	0	2.654372	-1.218733	-0.414072
7	6	0	3.910035	-0.655640	-0.185940
8	6	0	4.477467	-0.669202	1.095893
9	6	0	3.746746	-1.166936	2.181031
10	8	0	4.565765	0.002126	-1.214812
11	8	0	-4.484742	0.521213	0.733468
12	6	0	-3.831446	-0.504977	0.052603
13	6	0	-2.574870	-0.921591	0.487355
14	6	0	-1.844755	-1.801685	-0.320077
15	6	0	-2.409508	-2.311553	-1.506845
16	6	0	-3.709330	-1.970956	-1.869698
17	6	0	-4.421184	-1.046855	-1.094401
18	6	0	-0.403293	-2.081007	-0.283669
19	6	0	-0.037930	-2.550738	-1.689764
20	6	0	-1.370308	-3.110117	-2.262254
21	6	0	-4.001331	1.810924	0.367509
22	6	0	-2.689485	2.187346	1.068524
23	8	0	-1.906319	2.918814	0.256612
24	6	0	4.166204	1.393990	-1.316705
25	6	0	2.107663	2.851824	-1.850574
26	6	0	1.079115	2.540659	-0.748551
27	8	0	-2.421932	1.897443	2.216672
28	6	0	2.965361	1.632178	-2.252017
29	1	0	-0.774894	-2.297589	2.524550
30	1	0	-0.215729	-0.626213	2.405791
31	1	0	1.429324	-3.040781	3.252570
32	1	0	1.461010	-1.410014	3.925428
33	1	0	2.248882	-1.212575	-1.416881

34	1	0	5.465668	-0.242809	1.237172
35	1	0	4.177190	-1.141716	3.178624
36	1	0	-2.156867	-0.480334	1.381648
37	1	0	-4.153415	-2.366618	-2.779130
38	1	0	-5.408419	-0.708354	-1.392073
39	1	0	0.277268	-1.700594	-2.310985
40	1	0	0.776500	-3.280166	-1.695993
41	1	0	-1.460384	-4.181732	-2.039244
42	1	0	-1.447950	-2.998219	-3.347470
43	1	0	-4.757456	2.532634	0.695086
44	1	0	-3.883160	1.899729	-0.717064
45	1	0	5.047794	1.925559	-1.687305
46	1	0	3.952548	1.769362	-0.308562
47	1	0	2.761146	3.674632	-1.526427
48	1	0	1.575985	3.223700	-2.734930
49	1	0	0.365519	1.796639	-1.125412
50	1	0	1.581745	2.073011	0.108945
51	1	0	2.327403	0.743827	-2.254478
52	1	0	3.330938	1.748279	-3.278113
53	6	0	-0.705563	3.509336	0.839389
54	1	0	-0.318684	2.830865	1.603028
55	1	0	-1.014177	4.438361	1.328836
56	6	0	0.317689	3.780362	-0.257469
57	1	0	-0.181418	4.283628	-1.095157
58	1	0	1.034083	4.504364	0.152181

##

TS-M6[11]

Standard orientation:

Center Number	Atomic Number	Atomic Type	Coordinates (Angstroms) X	Y	Z
1	6	0	1.893891	-2.785933	-2.009558
2	6	0	1.522795	-2.234122	-0.643628
3	6	0	0.123812	-2.390285	-0.439464
4	6	0	-0.461491	-3.102599	-1.526724
5	6	0	0.609936	-3.507886	-2.516057
6	6	0	-0.686567	-1.870899	0.587883
7	6	0	-2.070792	-2.049066	0.521140
8	6	0	-2.644615	-2.798524	-0.523338
9	6	0	-1.827673	-3.314478	-1.548358

10	8	0	-2.778532	-1.429498	1.514724
11	8	0	2.471213	3.219057	-0.745124
12	6	0	2.908524	2.088534	-0.059492
13	6	0	2.446875	0.824274	-0.407940
14	6	0	2.885718	-0.283916	0.355392
15	6	0	3.805684	-0.094194	1.428449
16	6	0	4.281524	1.171882	1.726618
17	6	0	3.829160	2.274833	0.984252
18	6	0	2.465071	-1.639810	0.297605
19	6	0	3.075546	-2.402828	1.462348
20	6	0	4.093933	-1.417252	2.102869
21	6	0	1.185676	3.681476	-0.366894
22	6	0	0.058344	3.075745	-1.201793
23	8	0	-1.125145	3.339688	-0.620937
24	6	0	-4.207962	-1.329773	1.452381
25	6	0	-4.637874	1.210715	1.265250
26	6	0	-3.335930	1.522830	0.511726
27	8	0	0.204219	2.486641	-2.251734
28	6	0	-4.620249	-0.039253	2.163343
29	1	0	2.757810	-3.460024	-1.954226
30	1	0	2.190981	-1.974055	-2.690041
31	1	0	0.754311	-4.595821	-2.517513
32	1	0	0.353682	-3.227349	-3.543264
33	1	0	-0.266693	-1.283969	1.398120
34	1	0	-3.712166	-2.978838	-0.555113
35	1	0	-2.290254	-3.871290	-2.359558
36	1	0	1.736917	0.708586	-1.218683
37	1	0	4.984371	1.323489	2.541782
38	1	0	4.167230	3.280488	1.211030
39	1	0	2.295908	-2.698193	2.180107
40	1	0	3.547145	-3.336739	1.132303
41	1	0	5.123793	-1.739601	1.903759
42	1	0	3.991777	-1.357522	3.191563
43	1	0	1.165580	4.764060	-0.538520
44	1	0	0.986633	3.510292	0.696605
45	1	0	-4.646493	-2.211380	1.937228
46	1	0	-4.548371	-1.314068	0.410535
47	1	0	-5.444738	1.093459	0.527568
48	1	0	-4.917499	2.076814	1.879617
49	1	0	-2.510441	1.660397	1.220195
50	1	0	-3.059176	0.667788	-0.116752
51	1	0	-3.947524	0.103141	3.017471
52	1	0	-5.625112	-0.180339	2.579118
53	6	0	-2.324583	2.968787	-1.357933

54	1	0	-2.114254	2.063272	-1.931459
55	1	0	-2.539319	3.780597	-2.060462
56	6	0	-3.472786	2.763657	-0.382925
57	1	0	-3.602899	3.667067	0.226018
58	1	0	-4.381053	2.671150	-0.993763

##

MeOH

Standard orientation:

Center Number	Atomic Number	Atomic Type	Coordinates (Angstroms) X	Y	Z
1	8	0	0.749387	0.122466	-0.000003
2	1	0	1.134157	-0.766631	-0.000010
3	6	0	-0.662737	-0.019506	0.000010
4	1	0	-1.079110	0.991305	-0.000658
5	1	0	-1.036868	-0.544239	-0.892885
6	1	0	-1.036852	-0.543127	0.893518

##

TBD

Standard orientation:

Center Number	Atomic Number	Atomic Type	Coordinates (Angstroms) X	Y	Z
1	6	0	0.071526	-0.736690	-0.092487
2	7	0	1.133297	-1.469216	-0.109009
3	6	0	2.413538	-0.793575	-0.219175
4	1	0	3.184180	-1.434684	0.227253
5	1	0	2.694855	-0.672611	-1.279810
6	6	0	2.411363	0.577956	0.463726
7	1	0	3.361048	1.106597	0.318558
8	1	0	2.269523	0.439413	1.543067
9	6	0	1.263750	1.417330	-0.095299
10	1	0	1.514832	1.788916	-1.101380
11	1	0	1.095580	2.300039	0.536718
12	6	0	-1.205786	1.428474	0.015435
13	1	0	-1.259837	1.829209	1.043429

14	1	0	-1.155272	2.297188	-0.655580
15	6	0	-2.461162	0.611273	-0.281395
16	1	0	-2.557524	0.446078	-1.360263
17	1	0	-3.343519	1.163421	0.060650
18	6	0	-2.355208	-0.742439	0.414709
19	1	0	-3.218125	-1.372681	0.174563
20	1	0	-2.346025	-0.600943	1.511289
21	7	0	0.019021	0.649788	-0.147239
22	7	0	-1.158279	-1.393868	-0.085966
23	1	0	-1.026113	-2.370842	0.143926

##

E-M3[8]

Standard orientation:

Center Number	Atomic Number	Atomic Type	Coordinates (Angstroms) X	Y	Z
1	6	0	0.700049	-2.032892	2.102419
2	6	0	0.621601	-2.034590	0.575951
3	6	0	1.946349	-1.587715	0.124666
4	6	0	2.854389	-1.586528	1.201929
5	6	0	2.213725	-2.281444	2.384365
6	6	0	2.179251	-0.771941	-0.986670
7	6	0	3.240792	0.134375	-0.969380
8	6	0	4.182038	0.073442	0.067697
9	6	0	4.009448	-0.812470	1.139441
10	6	0	3.160508	1.312102	-1.918686
11	8	0	-3.384564	1.384546	1.367267
12	8	0	1.810807	1.871635	-1.870488
13	6	0	-3.261679	0.177363	0.707265
14	6	0	-2.134446	-0.597708	0.965302
15	6	0	-1.837700	-1.668252	0.111856
16	6	0	-2.727112	-2.017497	-0.924648
17	6	0	-3.927350	-1.329402	-1.075706
18	6	0	-4.193900	-0.219814	-0.263890
19	6	0	-0.489661	-2.165649	-0.192489
20	6	0	-0.544336	-2.598965	-1.657909
21	6	0	-2.039204	-2.987848	-1.860507
22	6	0	-3.153675	2.556332	0.552881
23	6	0	-1.942638	3.366517	1.032985
24	6	0	-0.529430	2.799847	0.790576

25	6	0	-0.022629	2.838349	-0.666135
26	6	0	1.475535	2.546611	-0.739661
27	8	0	2.280906	2.878521	0.102193
28	1	0	0.040365	-2.776353	2.560487
29	1	0	0.434068	-1.050246	2.519231
30	1	0	2.424946	-3.360135	2.384532
31	1	0	2.539300	-1.881397	3.349616
32	1	0	1.428399	-0.686298	-1.764435
33	1	0	5.001668	0.787033	0.091188
34	1	0	4.711926	-0.796248	1.969309
35	1	0	3.278254	1.032728	-2.968840
36	1	0	3.887769	2.085157	-1.660447
37	1	0	-1.430772	-0.253569	1.714099
38	1	0	-4.622197	-1.594123	-1.869498
39	1	0	-5.085805	0.379958	-0.419845
40	1	0	-0.318271	-1.762706	-2.335776
41	1	0	0.156261	-3.408525	-1.885397
42	1	0	-2.202123	-4.031521	-1.556358
43	1	0	-2.366690	-2.893763	-2.900840
44	1	0	-4.050643	3.182752	0.633703
45	1	0	-3.049206	2.257854	-0.497135
46	1	0	-2.069638	3.526120	2.111226
47	1	0	-2.016110	4.361203	0.567620
48	1	0	0.177850	3.378530	1.393864
49	1	0	-0.472728	1.771384	1.163924
50	1	0	-0.176534	3.843165	-1.086705
51	1	0	-0.556819	2.138050	-1.315216

##

INT1

Standard orientation:

Center Number	Atomic Number	Atomic Type	Coordinates (Angstroms) X	Y	Z
1	6	0	-3.756232	-1.177796	2.441988
2	6	0	-3.905796	-1.329016	0.928844
3	6	0	-2.994123	-2.420627	0.562099
4	6	0	-2.542882	-3.090022	1.716782
5	6	0	-3.344857	-2.608514	2.907516
6	6	0	-2.244092	-2.476292	-0.615330
7	6	0	-0.993944	-3.095647	-0.616072

8	6	0	-0.591041	-3.827000	0.509133
9	6	0	-1.377545	-3.849302	1.669876
10	6	0	-0.049354	-2.718344	-1.740215
11	8	0	-2.442707	3.818282	0.918790
12	8	0	-0.024161	-1.274000	-1.856397
13	6	0	-3.508314	3.092438	0.419103
14	6	0	-3.734918	1.797797	0.879355
15	6	0	-4.625038	0.975722	0.175889
16	6	0	-5.368585	1.495399	-0.902934
17	6	0	-5.248516	2.837279	-1.253027
18	6	0	-4.301396	3.636797	-0.601272
19	6	0	-4.541206	-0.486803	0.074196
20	6	0	-5.053837	-0.820704	-1.327525
21	6	0	-6.027659	0.352467	-1.644681
22	6	0	-1.173728	3.558379	0.270814
23	6	0	-0.369154	2.382760	0.875946
24	6	0	-0.380676	1.005917	0.163072
25	6	0	0.628279	0.869634	-0.996752
26	6	0	1.058371	-0.590633	-1.285986
27	8	0	1.516923	-1.247009	-0.177362
28	1	0	-4.664712	-0.802223	2.923594
29	1	0	-2.935313	-0.490886	2.696034
30	1	0	-4.241407	-3.223466	3.071029
31	1	0	-2.774075	-2.604649	3.841729
32	1	0	-2.522159	-1.867979	-1.468609
33	1	0	0.387471	-4.300454	0.517857
34	1	0	-1.014014	-4.362158	2.557631
35	1	0	-0.411808	-3.072781	-2.712029
36	1	0	0.956322	-3.108356	-1.572495
37	1	0	-3.104329	1.403133	1.666702
38	1	0	-5.819877	3.246776	-2.083176
39	1	0	-4.121886	4.660847	-0.914989
40	1	0	-4.242870	-0.797220	-2.069709
41	1	0	-5.522604	-1.808454	-1.381415
42	1	0	-7.028155	0.132619	-1.245246
43	1	0	-6.136557	0.537547	-2.718317
44	1	0	-0.618106	4.490747	0.410893
45	1	0	-1.339729	3.424564	-0.806734
46	1	0	-0.729616	2.265166	1.905483
47	1	0	0.677718	2.706253	0.969122
48	1	0	-0.120931	0.244399	0.906403
49	1	0	-1.387196	0.758123	-0.188016
50	1	0	1.554395	1.399370	-0.739350
51	1	0	0.235401	1.323731	-1.913779

52	8	0	2.174795	-0.606338	-2.230912
53	1	0	3.965031	-0.494229	-1.421699
54	6	0	1.923513	-0.073862	-3.523710
55	1	0	1.917473	1.025457	-3.528097
56	1	0	2.733928	-0.421162	-4.172123
57	1	0	0.969086	-0.439615	-3.917152
58	6	0	4.794176	0.149979	0.299628
59	7	0	4.872135	-0.312235	-0.991242
60	6	0	6.001620	-0.034256	-1.855417
61	1	0	5.965312	-0.730532	-2.699653
62	1	0	5.973567	0.987948	-2.273625
63	6	0	7.280408	-0.225160	-1.045163
64	1	0	8.166897	0.005023	-1.645973
65	1	0	7.347217	-1.271030	-0.724936
66	6	0	7.238407	0.694472	0.171490
67	1	0	7.437895	1.734562	-0.137564
68	1	0	8.030242	0.419537	0.881598
69	6	0	5.938635	1.235471	2.202102
70	1	0	6.372973	0.536813	2.934431
71	1	0	6.589859	2.120217	2.180906
72	6	0	4.522267	1.626952	2.614334
73	1	0	4.203193	2.509675	2.046445
74	1	0	4.507606	1.890717	3.678009
75	6	0	3.576590	0.464652	2.306867
76	1	0	2.541816	0.741826	2.539223
77	1	0	3.822113	-0.389645	2.960959
78	7	0	5.957242	0.627185	0.871337
79	7	0	3.641362	0.075221	0.907771
80	1	0	2.309614	-0.751709	0.212914

##

TS1

Standard orientation:

Center Number	Atomic Number	Atomic Type	Coordinates (Angstroms) X	Y	Z
1	6	0	-3.795232	1.747700	-2.050587
2	6	0	-3.913484	1.512758	-0.545505
3	6	0	-2.985094	2.472541	0.065526
4	6	0	-2.553997	3.412944	-0.890353
5	6	0	-3.380985	3.248410	-2.148387

6	6	0	-2.212451	2.227674	1.203954
7	6	0	-0.961020	2.829618	1.339263
8	6	0	-0.578693	3.823650	0.428490
9	6	0	-1.385868	4.138229	-0.673613
10	6	0	0.007911	2.184154	2.311810
11	8	0	-2.549761	-3.495214	-1.860520
12	8	0	0.043392	0.761527	2.063186
13	6	0	-3.595066	-2.904096	-1.175176
14	6	0	-3.801430	-1.531780	-1.290655
15	6	0	-4.662552	-0.898330	-0.385068
16	6	0	-5.401204	-1.661563	0.541924
17	6	0	-5.304867	-3.050047	0.540056
18	6	0	-4.384577	-3.675343	-0.310186
19	6	0	-4.546440	0.489159	0.082300
20	6	0	-5.026711	0.463603	1.533982
21	6	0	-6.022752	-0.732931	1.562780
22	6	0	-1.270395	-3.446908	-1.181478
23	6	0	-0.400179	-2.221569	-1.542126
24	6	0	-0.394897	-0.983989	-0.608399
25	6	0	0.588683	-1.061262	0.583726
26	6	0	0.953450	0.336872	1.108867
27	8	0	1.480410	1.178737	0.342607
28	1	0	-4.716502	1.509004	-2.591682
29	1	0	-2.985768	1.144050	-2.487224
30	1	0	-4.275742	3.887309	-2.135612
31	1	0	-2.826981	3.478248	-3.064452
32	1	0	-2.476335	1.422717	1.880868
33	1	0	0.401029	4.284883	0.523029
34	1	0	-1.038044	4.861011	-1.408692
35	1	0	-0.332106	2.271885	3.348994
36	1	0	1.011827	2.603492	2.222430
37	1	0	-3.172556	-0.964719	-1.966352
38	1	0	-5.871943	-3.645784	1.251979
39	1	0	-4.221234	-4.748082	-0.266999
40	1	0	-4.203641	0.234799	2.226053
41	1	0	-5.469796	1.413670	1.849141
42	1	0	-7.023749	-0.400188	1.252549
43	1	0	-6.119608	-1.182936	2.556202
44	1	0	-0.764216	-4.358771	-1.514447
45	1	0	-1.431089	-3.527647	-0.098391
46	1	0	-0.707631	-1.918583	-2.551319
47	1	0	0.637776	-2.570955	-1.643864
48	1	0	-0.092762	-0.117331	-1.208435
49	1	0	-1.404122	-0.767515	-0.243589

50	1	0	1.527222	-1.515575	0.246579
51	1	0	0.194811	-1.683420	1.394089
52	8	0	2.393459	-0.121670	2.251860
53	1	0	3.575494	-0.458034	1.500600
54	6	0	2.115248	-0.943892	3.345382
55	1	0	2.150535	-2.027833	3.111622
56	1	0	2.832698	-0.767307	4.168068
57	1	0	1.111158	-0.729362	3.743465
58	6	0	4.782123	-0.145452	-0.226611
59	7	0	4.514989	-0.721347	0.943163
60	6	0	5.473820	-1.596793	1.596952
61	1	0	5.265580	-1.571291	2.670564
62	1	0	5.343489	-2.639455	1.266747
63	6	0	6.897433	-1.131467	1.300734
64	1	0	7.635309	-1.820369	1.725326
65	1	0	7.058485	-0.145172	1.751366
66	6	0	7.087791	-1.049516	-0.210994
67	1	0	7.173911	-2.058402	-0.642982
68	1	0	8.016273	-0.516100	-0.451940
69	6	0	6.259042	0.181895	-2.198346
70	1	0	6.913062	1.064108	-2.120172
71	1	0	6.820469	-0.586620	-2.745162
72	6	0	4.977373	0.538181	-2.946866
73	1	0	4.450559	-0.375610	-3.244806
74	1	0	5.229402	1.093935	-3.856030
75	6	0	4.079284	1.362540	-2.029213
76	1	0	3.104725	1.553732	-2.486678
77	1	0	4.541880	2.340837	-1.823920
78	7	0	5.982239	-0.336287	-0.855627
79	7	0	3.846129	0.623614	-0.801559
80	1	0	2.929052	0.774539	-0.308915

##

INT2

Standard orientation:

Center Number	Atomic Number	Atomic Type	Coordinates (Angstroms) X	Y	Z
1	6	0	-1.129870	-2.051131	2.148670
2	6	0	-0.664073	-2.263756	0.709950
3	6	0	0.770414	-2.590291	0.778413

4	6	0	1.142503	-2.836976	2.116558
5	6	0	-0.058592	-2.746259	3.028886
6	6	0	1.751405	-2.599827	-0.226140
7	6	0	3.089373	-2.850289	0.099144
8	6	0	3.434824	-3.113777	1.431903
9	6	0	2.466544	-3.112589	2.440657
10	6	0	4.150241	-2.825493	-0.995672
11	8	0	-5.591709	0.168965	0.988174
12	8	0	3.807452	-2.030269	-2.104779
13	6	0	-4.893078	-0.576580	0.065111
14	6	0	-3.614493	-1.005963	0.432049
15	6	0	-2.844813	-1.739696	-0.479558
16	6	0	-3.372151	-2.045116	-1.752568
17	6	0	-4.658833	-1.647681	-2.095223
18	6	0	-5.428198	-0.913224	-1.186198
19	6	0	-1.445185	-2.187660	-0.395679
20	6	0	-1.028543	-2.574674	-1.812891
21	6	0	-2.357714	-2.799785	-2.579723
22	6	0	-6.171225	1.408868	0.554117
23	6	0	-5.509432	2.589770	1.270192
24	6	0	-3.984136	2.684242	1.106939
25	6	0	-3.505023	2.834901	-0.339730
26	6	0	-1.993611	2.919585	-0.452380
27	8	0	-1.220915	3.042640	0.477088
28	1	0	-2.135666	-2.444263	2.324082
29	1	0	-1.160602	-0.977625	2.387108
30	1	0	-0.395882	-3.749911	3.324224
31	1	0	0.147561	-2.198685	3.955573
32	1	0	1.519332	-2.376086	-1.260463
33	1	0	4.476643	-3.298899	1.686845
34	1	0	2.754267	-3.301912	3.472774
35	1	0	4.299075	-3.845491	-1.377547
36	1	0	5.110855	-2.517432	-0.547827
37	1	0	-3.237464	-0.708338	1.402214
38	1	0	-5.071474	-1.895082	-3.070701
39	1	0	-6.435786	-0.602411	-1.445484
40	1	0	-0.466695	-1.756176	-2.284650
41	1	0	-0.379203	-3.454808	-1.830333
42	1	0	-2.604951	-3.870351	-2.614886
43	1	0	-2.309382	-2.453057	-3.617971
44	1	0	-7.241210	1.379942	0.793208
45	1	0	-6.076167	1.504880	-0.533216
46	1	0	-5.739444	2.518902	2.340599
47	1	0	-5.992100	3.509829	0.907709

48	1	0	-3.613425	3.532159	1.693633
49	1	0	-3.522312	1.792828	1.543173
50	1	0	-3.923473	3.739466	-0.804434
51	1	0	-3.830609	1.998617	-0.968423
52	8	0	-1.606518	2.845486	-1.742064
53	1	0	3.815142	-1.082820	-1.800715
54	6	0	-0.189648	2.918073	-1.983472
55	1	0	0.198258	3.895666	-1.681911
56	1	0	0.342867	2.136713	-1.435245
57	1	0	-0.071633	2.781194	-3.058819
58	6	0	3.590202	1.304217	-0.253522
59	7	0	4.149003	0.600616	-1.193658
60	6	0	5.395737	1.085694	-1.764283
61	1	0	5.928701	0.227631	-2.189631
62	1	0	5.194052	1.771490	-2.604899
63	6	0	6.259255	1.799219	-0.722947
64	1	0	7.172656	2.211533	-1.167022
65	1	0	6.558347	1.080243	0.049793
66	6	0	5.442761	2.922914	-0.088953
67	1	0	5.363716	3.776908	-0.779505
68	1	0	5.940369	3.291560	0.817953
69	6	0	3.499117	3.175674	1.416488
70	1	0	4.063455	2.939239	2.334777
71	1	0	3.617484	4.254086	1.244251
72	6	0	2.025537	2.831911	1.608289
73	1	0	1.403913	3.316992	0.850512
74	1	0	1.693701	3.194329	2.587144
75	6	0	1.836313	1.323356	1.502707
76	1	0	0.773165	1.071491	1.545473
77	1	0	2.340740	0.810214	2.339258
78	7	0	4.101572	2.467887	0.285394
79	7	0	2.349546	0.894536	0.207759
80	1	0	2.152209	-0.064348	-0.057241

##

TS2

Standard orientation:

Center Number	Atomic Number	Atomic Type	Coordinates (Angstroms) X	Y	Z
1	6	0	-4.442670	2.207030	-1.086909

2	6	0	-3.658793	1.918813	0.193677
3	6	0	-2.416770	2.693283	0.063334
4	6	0	-2.530281	3.609577	-1.000218
5	6	0	-3.969105	3.643106	-1.470279
6	6	0	-1.155645	2.236531	0.470167
7	6	0	-0.023811	2.598888	-0.257607
8	6	0	-0.135740	3.588095	-1.248702
9	6	0	-1.383409	4.113139	-1.607880
10	6	0	1.251731	1.777363	-0.117221
11	8	0	-3.466156	-3.109537	-1.377489
12	8	0	1.028115	0.437651	0.324429
13	6	0	-4.057708	-2.479994	-0.298997
14	6	0	-4.199631	-1.096633	-0.347571
15	6	0	-4.427771	-0.395957	0.845139
16	6	0	-4.617062	-1.093068	2.054308
17	6	0	-4.654809	-2.485356	2.055337
18	6	0	-4.366198	-3.184335	0.876300
19	6	0	-3.926456	0.958176	1.114786
20	6	0	-3.503264	0.937931	2.583056
21	6	0	-4.461527	-0.123986	3.207933
22	6	0	-2.067874	-3.432794	-1.138136
23	6	0	-1.145168	-2.946646	-2.255480
24	6	0	-1.018807	-1.427822	-2.489143
25	6	0	-0.615375	-0.500184	-1.323481
26	6	0	0.816894	-0.658027	-0.785652
27	8	0	1.770751	-0.586121	-1.678387
28	1	0	-5.525268	2.123185	-0.948099
29	1	0	-4.156232	1.525630	-1.901508
30	1	0	-4.562111	4.391947	-0.925725
31	1	0	-4.071231	3.855183	-2.539494
32	1	0	-1.067331	1.432181	1.191177
33	1	0	0.745584	3.877707	-1.817994
34	1	0	-1.456146	4.815511	-2.435548
35	1	0	1.930388	2.232012	0.621074
36	1	0	1.780187	1.765842	-1.079624
37	1	0	-3.924775	-0.583387	-1.261854
38	1	0	-4.805766	-3.035265	2.981886
39	1	0	-4.282787	-4.267252	0.881234
40	1	0	-2.470554	0.578869	2.702873
41	1	0	-3.571335	1.923105	3.055873
42	1	0	-5.416085	0.347349	3.483031
43	1	0	-4.051431	-0.588499	4.110674
44	1	0	-1.991517	-4.526045	-1.060223
45	1	0	-1.753338	-3.004475	-0.181825

46	1	0	-1.471133	-3.406550	-3.198830
47	1	0	-0.157211	-3.369286	-2.032757
48	1	0	-0.297080	-1.278864	-3.301796
49	1	0	-1.984008	-1.064544	-2.867114
50	1	0	-1.306193	-0.606747	-0.482140
51	1	0	-0.718372	0.525220	-1.689748
52	8	0	0.793442	-1.832243	0.042398
53	1	0	2.593101	0.064132	1.043431
54	6	0	1.907164	-2.701800	-0.022821
55	1	0	2.265280	-2.820753	-1.051073
56	1	0	2.745883	-2.360755	0.601424
57	1	0	1.565647	-3.668139	0.362293
58	6	0	4.518296	-0.034611	0.363622
59	7	0	3.587527	0.027436	1.341220
60	6	0	3.919638	0.084581	2.753876
61	1	0	3.074957	0.540941	3.277249
62	1	0	4.055735	-0.925069	3.171652
63	6	0	5.194391	0.904587	2.934041
64	1	0	5.514701	0.909195	3.981064
65	1	0	5.003825	1.941578	2.634379
66	6	0	6.298960	0.305142	2.068066
67	1	0	6.668861	-0.630501	2.515531
68	1	0	7.153001	0.992194	2.009920
69	6	0	6.900577	-0.176109	-0.289113
70	1	0	7.355048	0.791029	-0.552364
71	1	0	7.684255	-0.781804	0.184148
72	6	0	6.368730	-0.872927	-1.538751
73	1	0	6.158472	-1.926052	-1.318496
74	1	0	7.129271	-0.840104	-2.326074
75	6	0	5.079941	-0.183256	-1.982020
76	1	0	4.612974	-0.710046	-2.819418
77	1	0	5.299638	0.839719	-2.325827
78	7	0	5.842021	0.041240	0.701316
79	7	0	4.118240	-0.166486	-0.891056
80	1	0	2.988685	-0.368117	-1.181312

###

M3[8]

Standard orientation:

Center Number	Atomic Number	Atomic Type	Coordinates (Angstroms) X	Y	Z

1	6	0	4.380460	-0.506737	-0.112397
2	6	0	2.921301	-0.056864	-0.028033
3	6	0	2.113656	-1.291269	-0.103664
4	6	0	2.921035	-2.360743	-0.541976
5	6	0	4.328634	-1.885490	-0.819669
6	6	0	0.791051	-1.540648	0.283836
7	6	0	0.256835	-2.827653	0.168697
8	6	0	1.058043	-3.869908	-0.318451
9	6	0	2.394277	-3.642941	-0.657701
10	6	0	-1.156960	-3.121363	0.613611
11	8	0	-2.193177	1.855312	-1.142777
12	8	0	-1.955419	-1.935704	0.426859
13	6	0	-1.065252	2.334942	-0.526247
14	6	0	0.050894	1.492550	-0.563506
15	6	0	1.248164	1.908624	0.023351
16	6	0	1.338202	3.198078	0.590809
17	6	0	0.222724	4.025547	0.631474
18	6	0	-0.991207	3.594694	0.083269
19	6	0	2.565091	1.244845	0.091383
20	6	0	3.574146	2.355408	0.383652
21	6	0	2.743711	3.465149	1.080404
22	6	0	-3.470269	2.213747	-0.616410
23	6	0	-4.482128	1.170659	-1.094305
24	6	0	-4.059298	-0.283490	-0.827161
25	6	0	-3.912756	-0.641302	0.666431
26	6	0	-3.231496	-1.979609	0.881798
27	8	0	-3.727341	-2.961738	1.389018
28	1	0	4.800656	-0.626334	0.897444
29	1	0	5.014024	0.212569	-0.640003
30	1	0	5.093503	-2.581362	-0.457531
31	1	0	4.491478	-1.767336	-1.900436
32	1	0	0.171366	-0.748013	0.681914
33	1	0	0.639469	-4.869022	-0.418552
34	1	0	3.013383	-4.465635	-1.008005
35	1	0	-1.203386	-3.404813	1.672210
36	1	0	-1.593826	-3.946896	0.041401
37	1	0	-0.047964	0.535845	-1.060932
38	1	0	0.286465	5.016521	1.075401
39	1	0	-1.857959	4.247388	0.102622
40	1	0	4.414903	2.012881	0.994442
41	1	0	3.994497	2.745391	-0.555438
42	1	0	3.096222	4.474478	0.840098
43	1	0	2.806199	3.357528	2.172830

44	1	0	-3.767229	3.213205	-0.965793
45	1	0	-3.417258	2.249579	0.480450
46	1	0	-4.626680	1.293380	-2.174852
47	1	0	-5.447960	1.393677	-0.620116
48	1	0	-4.794563	-0.957347	-1.284124
49	1	0	-3.104472	-0.460398	-1.328315
50	1	0	-4.885990	-0.680947	1.163818
51	1	0	-3.300533	0.115328	1.171498

###

INT3

Standard orientation:

Center Number	Atomic Number	Atomic Type	Coordinates (Angstroms) X	Y	Z
1	6	0	3.994678	-4.034099	-0.224674
2	6	0	3.674478	-2.542149	-0.125862
3	6	0	2.302384	-2.386988	-0.652597
4	6	0	1.926558	-3.557629	-1.339168
5	6	0	3.052542	-4.565857	-1.335338
6	6	0	1.364272	-1.358790	-0.470900
7	6	0	0.087480	-1.470392	-1.024640
8	6	0	-0.252551	-2.622856	-1.751289
9	6	0	0.656587	-3.671179	-1.898141
10	6	0	-0.964884	-0.397508	-0.827314
11	8	0	3.254721	2.928445	-1.029790
12	8	0	-0.361774	0.763941	-0.272054
13	6	0	3.987087	2.112067	-0.207827
14	6	0	3.814985	0.738617	-0.403663
15	6	0	4.532402	-0.164730	0.382667
16	6	0	5.473457	0.314587	1.318952
17	6	0	5.636576	1.681292	1.511558
18	6	0	4.885131	2.589947	0.756971
19	6	0	4.560099	-1.639264	0.359738
20	6	0	5.855645	-2.041104	1.065072
21	6	0	6.189120	-0.838205	1.987276
22	6	0	2.727056	4.143944	-0.494814
23	6	0	1.478427	4.505684	-1.298964
24	6	0	0.404267	3.404889	-1.337557
25	6	0	-0.223473	3.087429	0.025801
26	6	0	-1.175291	1.885117	-0.015897

27	8	0	-2.169769	2.097948	-0.940285
28	1	0	3.763921	-4.537439	0.726377
29	1	0	5.050083	-4.226047	-0.441014
30	1	0	2.709277	-5.590165	-1.150309
31	1	0	3.571912	-4.574844	-2.304491
32	1	0	1.601006	-0.479240	0.111636
33	1	0	-1.245429	-2.706415	-2.189510
34	1	0	0.369377	-4.570245	-2.439311
35	1	0	-1.754251	-0.779901	-0.166341
36	1	0	-1.438501	-0.151720	-1.786573
37	1	0	3.123860	0.415482	-1.171630
38	1	0	6.355332	2.054697	2.237871
39	1	0	5.020664	3.657920	0.895839
40	1	0	5.762832	-2.981086	1.617868
41	1	0	6.664127	-2.176963	0.330958
42	1	0	7.266928	-0.669953	2.093371
43	1	0	5.793345	-1.013431	2.998181
44	1	0	3.473167	4.949030	-0.568588
45	1	0	2.492277	4.003569	0.568508
46	1	0	1.786485	4.733739	-2.327382
47	1	0	1.068325	5.436782	-0.881200
48	1	0	-0.390658	3.707114	-2.028246
49	1	0	0.849691	2.493363	-1.744686
50	1	0	-0.775384	3.962624	0.387079
51	1	0	0.544175	2.847950	0.769538
52	8	0	-1.750746	1.621108	1.293947
53	1	0	-3.063084	0.152874	1.358738
54	6	0	-2.550318	2.667869	1.839091
55	1	0	-1.930664	3.468360	2.262356
56	1	0	-3.228395	3.086867	1.087800
57	1	0	-3.140620	2.224510	2.646558
58	6	0	-4.757202	-0.282360	0.346814
59	7	0	-3.858732	-0.485444	1.369216
60	6	0	-4.213913	-1.140921	2.612821
61	1	0	-3.289133	-1.434176	3.119292
62	1	0	-4.766087	-0.473896	3.298445
63	6	0	-5.068748	-2.359798	2.280553
64	1	0	-5.393487	-2.874150	3.191419
65	1	0	-4.473326	-3.062151	1.686420
66	6	0	-6.292404	-1.906157	1.489617
67	1	0	-7.018591	-1.424359	2.165955
68	1	0	-6.801358	-2.773952	1.048139
69	6	0	-7.007393	-0.739591	-0.566450
70	1	0	-7.055844	-1.577703	-1.279364

71	1	0	-7.965502	-0.723167	-0.028690
72	6	0	-6.788717	0.577546	-1.305772
73	1	0	-7.012127	1.418142	-0.637318
74	1	0	-7.472921	0.639022	-2.159755
75	6	0	-5.326309	0.663061	-1.746050
76	1	0	-5.127247	1.626655	-2.228922
77	1	0	-5.127518	-0.111501	-2.506646
78	7	0	-5.944482	-0.985697	0.408571
79	7	0	-4.415187	0.522643	-0.620485
80	1	0	-2.939479	1.466913	-0.778115

###

TS3

Standard orientation:

Center Number	Atomic Number	Atomic Type	Coordinates (Angstroms) X	Y	Z
1	6	0	3.948539	-3.999669	-0.211630
2	6	0	3.607442	-2.511514	-0.123227
3	6	0	2.264604	-2.367872	-0.724107
4	6	0	1.939742	-3.539737	-1.434675
5	6	0	3.076450	-4.534341	-1.376358
6	6	0	1.305866	-1.351394	-0.587985
7	6	0	0.059115	-1.477751	-1.204372
8	6	0	-0.228166	-2.632024	-1.950811
9	6	0	0.701549	-3.667097	-2.058040
10	6	0	-1.016373	-0.419128	-1.057763
11	8	0	3.195695	2.964910	-1.012826
12	8	0	-0.486416	0.721781	-0.405298
13	6	0	3.880395	2.147364	-0.153417
14	6	0	3.734133	0.773757	-0.370474
15	6	0	4.410483	-0.129528	0.451507
16	6	0	5.287102	0.349886	1.448083
17	6	0	5.423047	1.716180	1.663058
18	6	0	4.710920	2.624140	0.870930
19	6	0	4.454406	-1.603519	0.418448
20	6	0	5.710505	-1.998463	1.195289
21	6	0	5.974119	-0.801747	2.147704
22	6	0	2.629413	4.175141	-0.502379
23	6	0	1.439605	4.545006	-1.387429
24	6	0	0.381807	3.436993	-1.520320

25	6	0	-0.326867	3.068443	-0.206165
26	6	0	-1.263509	1.883107	-0.412896
27	8	0	-2.261269	1.971051	-1.171403
28	1	0	3.669240	-4.511055	0.722019
29	1	0	5.016377	-4.180020	-0.368871
30	1	0	2.736750	-5.564422	-1.218381
31	1	0	3.648802	-4.528808	-2.315289
32	1	0	1.499731	-0.470807	0.008953
33	1	0	-1.196917	-2.727417	-2.438669
34	1	0	0.454532	-4.567050	-2.617490
35	1	0	-1.853996	-0.831090	-0.475970
36	1	0	-1.417647	-0.134669	-2.039170
37	1	0	3.094829	0.450298	-1.182103
38	1	0	6.090561	2.089766	2.436666
39	1	0	4.824894	3.691941	1.028746
40	1	0	5.596734	-2.945475	1.732002
41	1	0	6.562689	-2.116680	0.509113
42	1	0	7.041707	-0.623029	2.319948
43	1	0	5.520743	-0.992060	3.131350
44	1	0	3.378706	4.980561	-0.514378
45	1	0	2.318594	4.020968	0.538855
46	1	0	1.817723	4.798431	-2.386504
47	1	0	0.992002	5.462494	-0.978560
48	1	0	-0.375257	3.750016	-2.249042
49	1	0	0.864780	2.543642	-1.928763
50	1	0	-0.913123	3.921471	0.149901
51	1	0	0.390194	2.799384	0.574151
52	8	0	-1.934560	1.720081	1.328421
53	1	0	-2.817504	0.615630	1.314700
54	6	0	-2.731742	2.815468	1.693615
55	1	0	-2.152963	3.578474	2.239354
56	1	0	-3.194223	3.298083	0.815183
57	1	0	-3.554326	2.496086	2.358578
58	6	0	-4.583447	-0.252899	0.500649
59	7	0	-3.600934	-0.201464	1.396777
60	6	0	-3.660704	-0.955554	2.638163
61	1	0	-2.633283	-1.112757	2.979150
62	1	0	-4.177447	-0.380111	3.422745
63	6	0	-4.378199	-2.281614	2.405217
64	1	0	-4.496878	-2.836010	3.342007
65	1	0	-3.786210	-2.900410	1.720785
66	6	0	-5.751936	-2.005306	1.799039
67	1	0	-6.435203	-1.600375	2.560977
68	1	0	-6.199491	-2.934864	1.425539

69	6	0	-6.789088	-1.088299	-0.267204
70	1	0	-6.706134	-1.967376	-0.925061
71	1	0	-7.709926	-1.209000	0.316849
72	6	0	-6.850346	0.192438	-1.095359
73	1	0	-7.162470	1.030885	-0.462059
74	1	0	-7.591734	0.077418	-1.892710
75	6	0	-5.468625	0.480225	-1.674088
76	1	0	-5.441609	1.449093	-2.180632
77	1	0	-5.203781	-0.285802	-2.419905
78	7	0	-5.665541	-1.069777	0.673073
79	7	0	-4.495670	0.519276	-0.596049
80	1	0	-3.613501	1.061681	-0.759786

##

INT4

Standard orientation:

Center Number	Atomic Number	Atomic Type	Coordinates (Angstroms) X	Y	Z
1	6	0	4.980197	-2.815517	0.421925
2	6	0	4.064171	-1.595353	0.300849
3	6	0	2.752465	-2.015950	0.835264
4	6	0	2.728928	-3.420448	0.977069
5	6	0	4.023573	-4.031074	0.494353
6	6	0	1.659578	-1.263796	1.285806
7	6	0	0.537736	-1.900917	1.827528
8	6	0	0.519083	-3.298767	1.922586
9	6	0	1.616064	-4.060718	1.510055
10	6	0	-0.632134	-1.069089	2.333105
11	8	0	0.725105	2.518328	-1.469875
12	8	0	-0.911993	0.062021	1.532968
13	6	0	2.061021	2.375564	-1.201023
14	6	0	2.466761	1.084224	-0.839535
15	6	0	3.811300	0.835663	-0.555588
16	6	0	4.757133	1.877236	-0.691424
17	6	0	4.347007	3.155269	-1.042210
18	6	0	2.993999	3.418080	-1.288716
19	6	0	4.508305	-0.418232	-0.203656
20	6	0	5.989056	-0.167580	-0.496305
21	6	0	6.153980	1.370839	-0.416362
22	6	0	0.175830	3.831499	-1.508631

23	6	0	-1.341103	3.720738	-1.632421
24	6	0	-2.029425	2.844678	-0.573166
25	6	0	-1.682522	3.217884	0.870663
26	6	0	-2.586182	2.555273	1.889016
27	8	0	-3.605740	1.933480	1.647797
28	1	0	5.571602	-2.754222	1.347885
29	1	0	5.692354	-2.890850	-0.405699
30	1	0	4.392349	-4.826649	1.151797
31	1	0	3.895764	-4.477597	-0.502387
32	1	0	1.660818	-0.182108	1.239484
33	1	0	-0.358490	-3.794595	2.333009
34	1	0	1.597423	-5.144195	1.608700
35	1	0	-0.401805	-0.687102	3.337579
36	1	0	-1.516261	-1.720143	2.435227
37	1	0	1.712378	0.308750	-0.800049
38	1	0	5.072222	3.960479	-1.138294
39	1	0	2.685017	4.418761	-1.568249
40	1	0	6.651896	-0.696175	0.195494
41	1	0	6.240284	-0.513722	-1.510315
42	1	0	6.896746	1.757114	-1.123723
43	1	0	6.488434	1.665844	0.588821
44	1	0	0.578513	4.391575	-2.365636
45	1	0	0.459894	4.373687	-0.594940
46	1	0	-1.585778	3.325576	-2.626349
47	1	0	-1.740293	4.744860	-1.595975
48	1	0	-3.114116	2.919136	-0.711903
49	1	0	-1.766024	1.795657	-0.739421
50	1	0	-1.754778	4.303718	1.034469
51	1	0	-0.655821	2.931165	1.118848
52	8	0	-2.149160	2.779711	3.141421
53	1	0	-1.264625	-0.282646	0.663350
54	6	0	-2.905656	2.146706	4.183731
55	1	0	-3.940715	2.499773	4.182367
56	1	0	-2.895718	1.062132	4.049114
57	1	0	-2.408854	2.422485	5.114638
58	6	0	-3.320475	-1.178412	-0.834910
59	7	0	-2.028776	-1.017481	-0.771920
60	6	0	-1.197269	-1.729996	-1.729433
61	1	0	-0.255276	-1.181513	-1.840855
62	1	0	-0.929085	-2.727054	-1.341748
63	6	0	-1.889197	-1.875658	-3.085503
64	1	0	-1.283681	-2.461696	-3.786526
65	1	0	-2.036506	-0.880507	-3.523056
66	6	0	-3.242305	-2.552359	-2.884991

67	1	0	-3.109799	-3.630200	-2.699963
68	1	0	-3.856093	-2.458013	-3.791435
69	6	0	-5.432499	-2.099692	-1.836984
70	1	0	-5.829557	-1.492046	-2.667960
71	1	0	-5.657827	-3.147701	-2.079029
72	6	0	-6.116966	-1.700230	-0.531881
73	1	0	-5.953307	-2.469773	0.231096
74	1	0	-7.195883	-1.611306	-0.700020
75	6	0	-5.522508	-0.383958	-0.043296
76	1	0	-5.943589	-0.089148	0.922196
77	1	0	-5.753928	0.423819	-0.759782
78	7	0	-3.979697	-1.948307	-1.774972
79	7	0	-4.095690	-0.578456	0.132844
80	1	0	-3.612115	0.140377	0.663600

##

TS4

Standard orientation:

Center Number	Atomic Number	Atomic Type	Coordinates (Angstroms) X	Y	Z
1	6	0	4.592900	-3.115748	0.178189
2	6	0	3.835421	-1.787755	0.113651
3	6	0	2.485102	-2.062875	0.650327
4	6	0	2.289024	-3.459816	0.735680
5	6	0	3.490792	-4.203451	0.201037
6	6	0	1.504377	-1.201351	1.160073
7	6	0	0.326542	-1.715212	1.720182
8	6	0	0.139551	-3.103743	1.759809
9	6	0	1.119844	-3.978546	1.277982
10	6	0	-0.713410	-0.765112	2.295772
11	8	0	1.002427	2.748699	-1.489939
12	8	0	-1.028025	0.255152	1.385000
13	6	0	2.306532	2.435847	-1.209294
14	6	0	2.564005	1.093850	-0.911125
15	6	0	3.872258	0.683877	-0.643197
16	6	0	4.927453	1.617832	-0.730103
17	6	0	4.663268	2.952279	-1.012906
18	6	0	3.348930	3.372656	-1.243900
19	6	0	4.418672	-0.654523	-0.347317
20	6	0	5.918488	-0.562631	-0.634344

21	6	0	6.257057	0.942709	-0.482467
22	6	0	0.498330	4.010733	-1.042569
23	6	0	-1.026329	3.930537	-1.027076
24	6	0	-1.597099	2.752516	-0.220822
25	6	0	-1.234292	2.757742	1.264381
26	6	0	-1.932252	1.653531	2.069884
27	8	0	-3.168370	1.426324	1.920669
28	1	0	5.183385	-3.170177	1.105118
29	1	0	5.292411	-3.243446	-0.653772
30	1	0	3.761386	-5.073708	0.809721
31	1	0	3.300489	-4.575010	-0.816622
32	1	0	1.642509	-0.127200	1.157226
33	1	0	-0.775463	-3.504300	2.191907
34	1	0	0.970954	-5.054836	1.340737
35	1	0	-0.314682	-0.321040	3.220909
36	1	0	-1.603292	-1.355867	2.588670
37	1	0	1.726607	0.407997	-0.898970
38	1	0	5.474300	3.675222	-1.068567
39	1	0	3.142498	4.412181	-1.476771
40	1	0	6.515867	-1.195658	0.028846
41	1	0	6.130263	-0.885621	-1.664896
42	1	0	7.041476	1.274437	-1.172353
43	1	0	6.619188	1.150242	0.534814
44	1	0	0.828413	4.815958	-1.715760
45	1	0	0.895836	4.224595	-0.041900
46	1	0	-1.380576	3.859636	-2.064353
47	1	0	-1.401601	4.885843	-0.632296
48	1	0	-2.690255	2.766655	-0.309850
49	1	0	-1.249149	1.819476	-0.672403
50	1	0	-1.553904	3.707062	1.719913
51	1	0	-0.155639	2.669346	1.417209
52	8	0	-1.442013	1.722526	3.379170
53	1	0	-1.819274	-0.404088	0.161490
54	6	0	-2.342766	1.290385	4.392384
55	1	0	-3.287761	1.840395	4.343459
56	1	0	-2.565383	0.219229	4.321124
57	1	0	-1.837051	1.492865	5.340712
58	6	0	-3.587687	-0.731221	-0.882179
59	7	0	-2.283313	-0.909301	-0.662301
60	6	0	-1.475806	-1.828459	-1.450433
61	1	0	-0.440401	-1.483371	-1.399917
62	1	0	-1.497374	-2.838917	-1.016483
63	6	0	-1.991723	-1.852245	-2.885609
64	1	0	-1.451042	-2.593475	-3.483045

65	1	0	-1.835314	-0.869898	-3.346152
66	6	0	-3.479679	-2.192926	-2.878840
67	1	0	-3.629232	-3.259118	-2.650376
68	1	0	-3.919482	-2.009476	-3.867249
69	6	0	-5.664181	-1.285422	-2.113274
70	1	0	-5.871001	-0.597601	-2.947514
71	1	0	-6.022535	-2.277031	-2.417095
72	6	0	-6.386814	-0.823337	-0.850069
73	1	0	-6.378775	-1.624597	-0.102159
74	1	0	-7.430729	-0.596010	-1.089551
75	6	0	-5.674712	0.402562	-0.286009
76	1	0	-6.091154	0.697833	0.680956
77	1	0	-5.794186	1.258569	-0.968361
78	7	0	-4.216371	-1.379464	-1.906339
79	7	0	-4.271212	0.091388	-0.080501
80	1	0	-3.756983	0.606855	0.691477

###

12. NMR spectrum of products

^{1}H NMR (700 MHz, $CDCl_3$) and ^{13}C NMR (176 MHz, $CDCl_3$) spectra for M1[4]

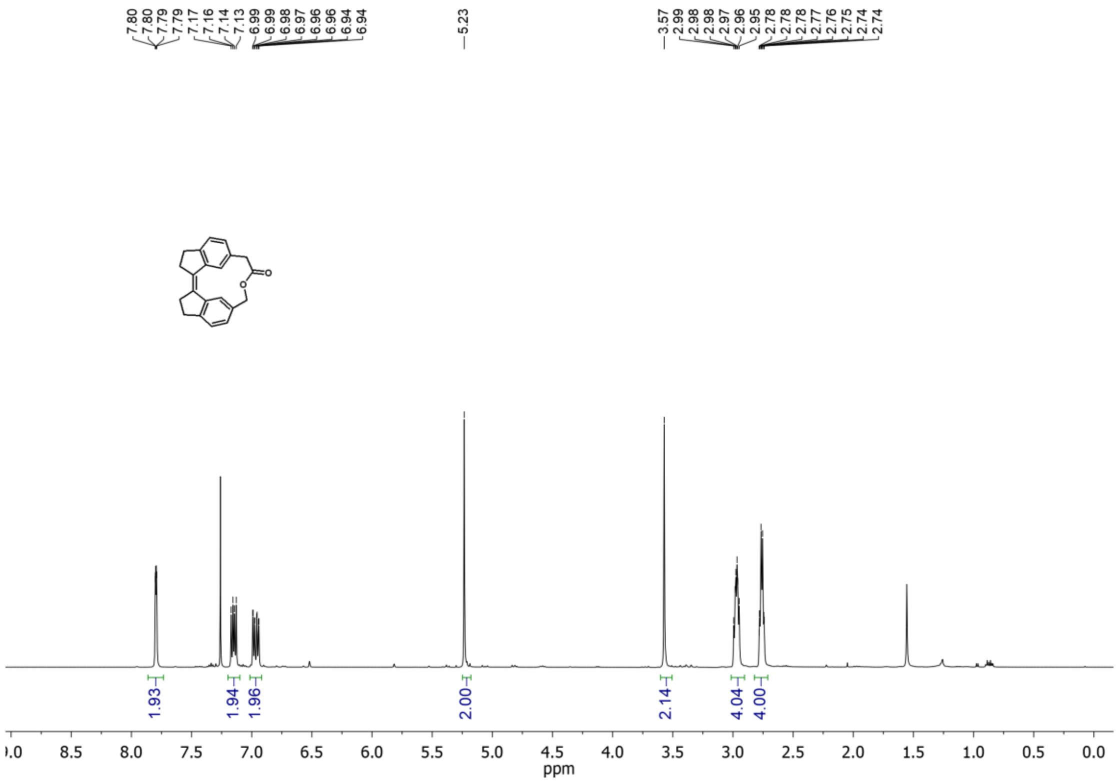

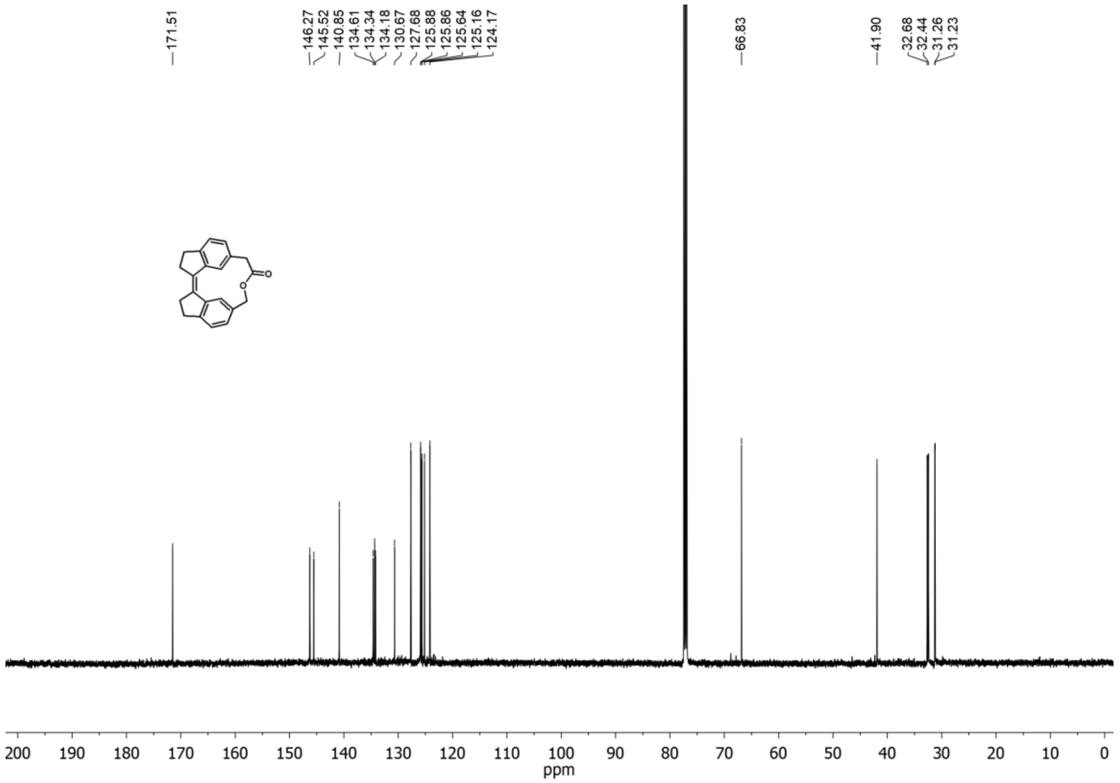

^{1}H NMR (700 MHz, $CDCl_3$) and ^{13}C NMR (176 MHz, $CDCl_3$) spectra for M2[5]

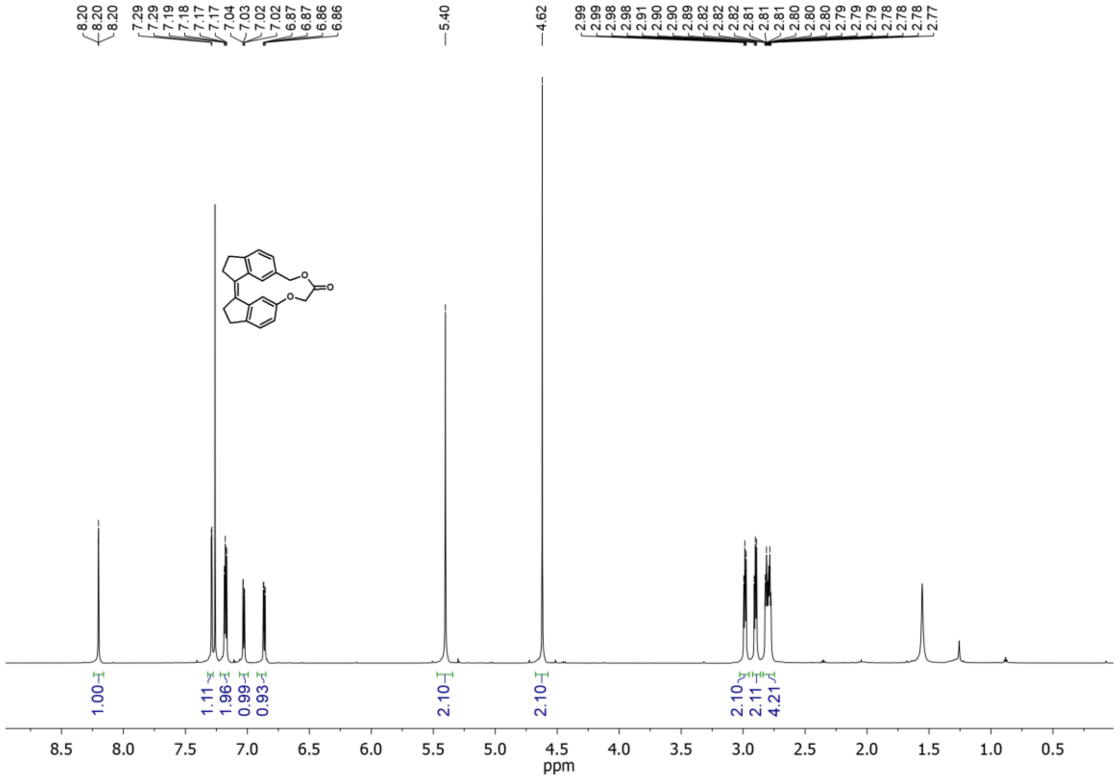

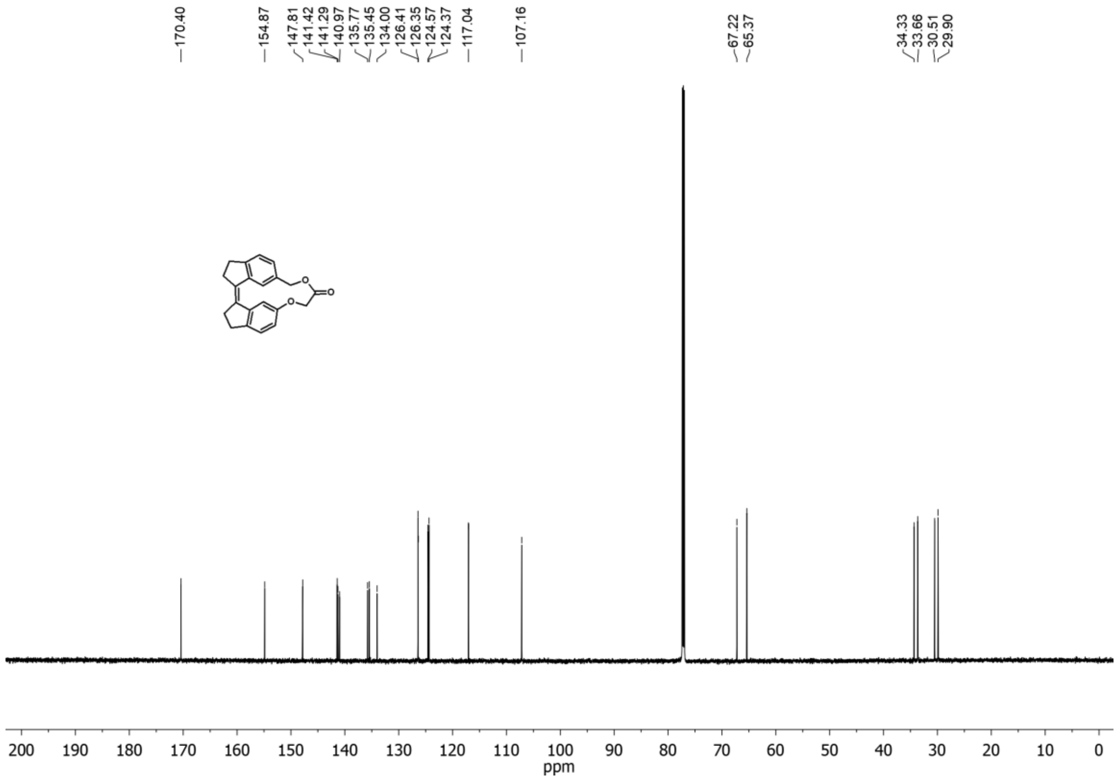

^{1}H NMR (700 MHz, $CDCl_3$) and ^{13}C NMR (126 MHz, $CDCl_3$) spectra for M3[8]

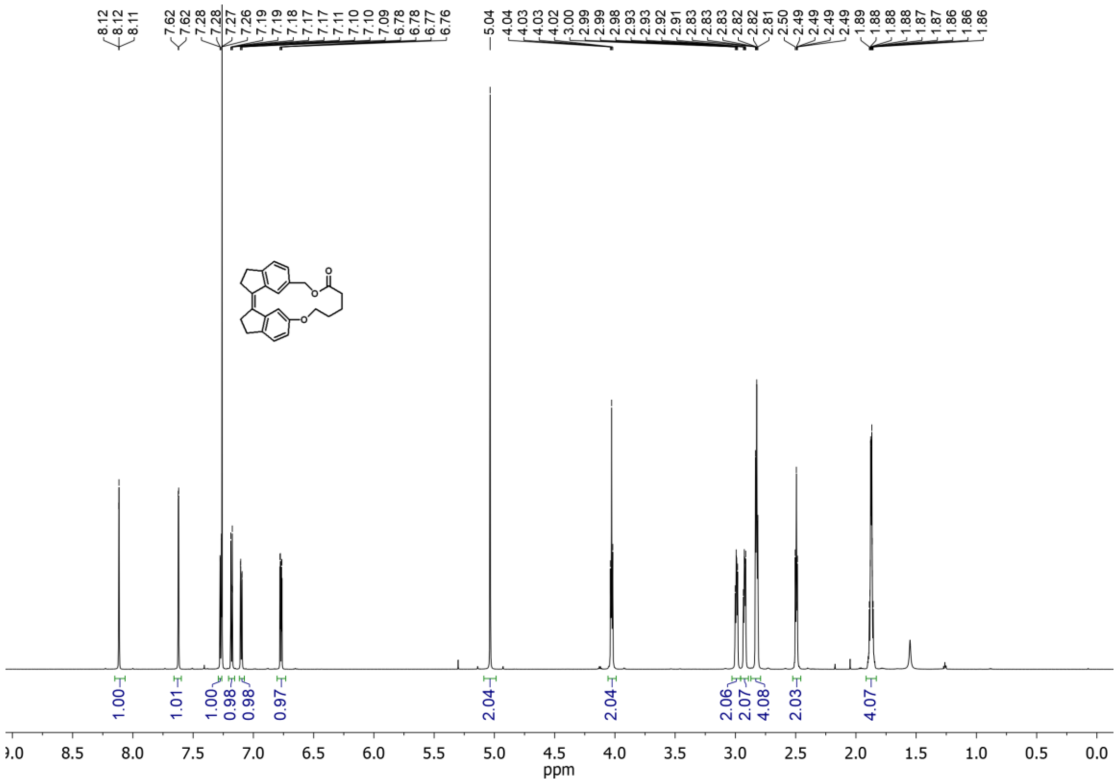

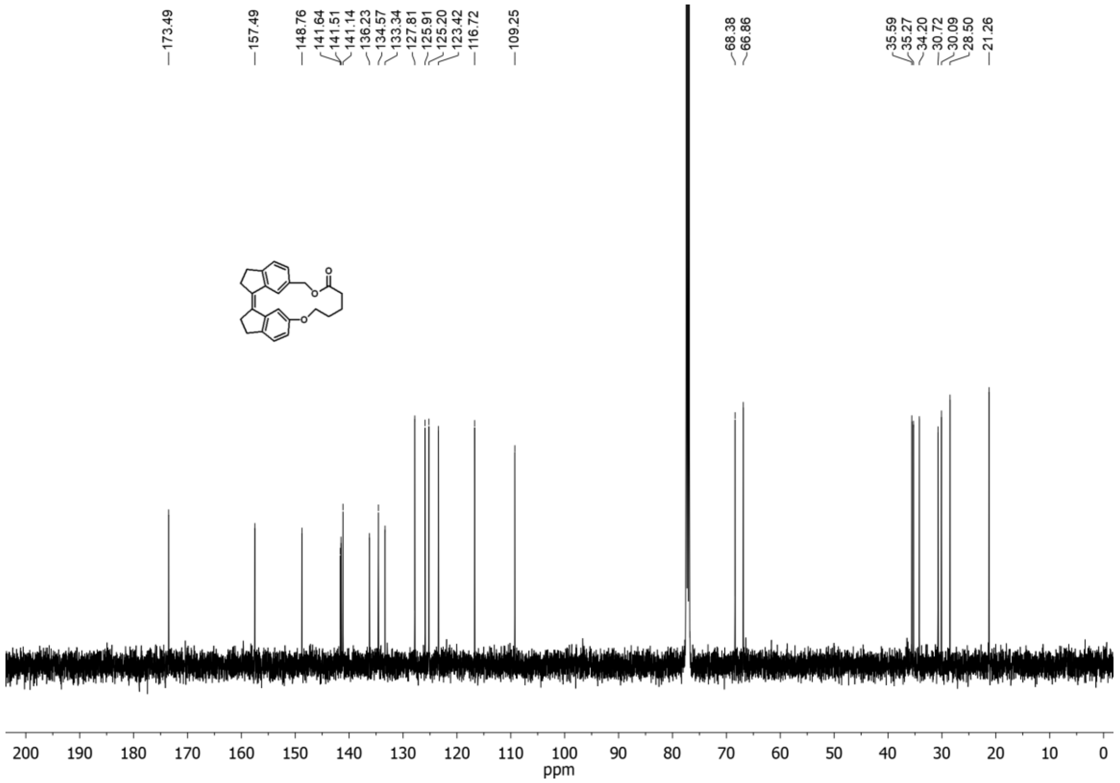

^{1}H NMR (700 MHz, $CDCl_3$) and ^{13}C NMR (176 MHz, $CDCl_3$) spectra for M4[8]

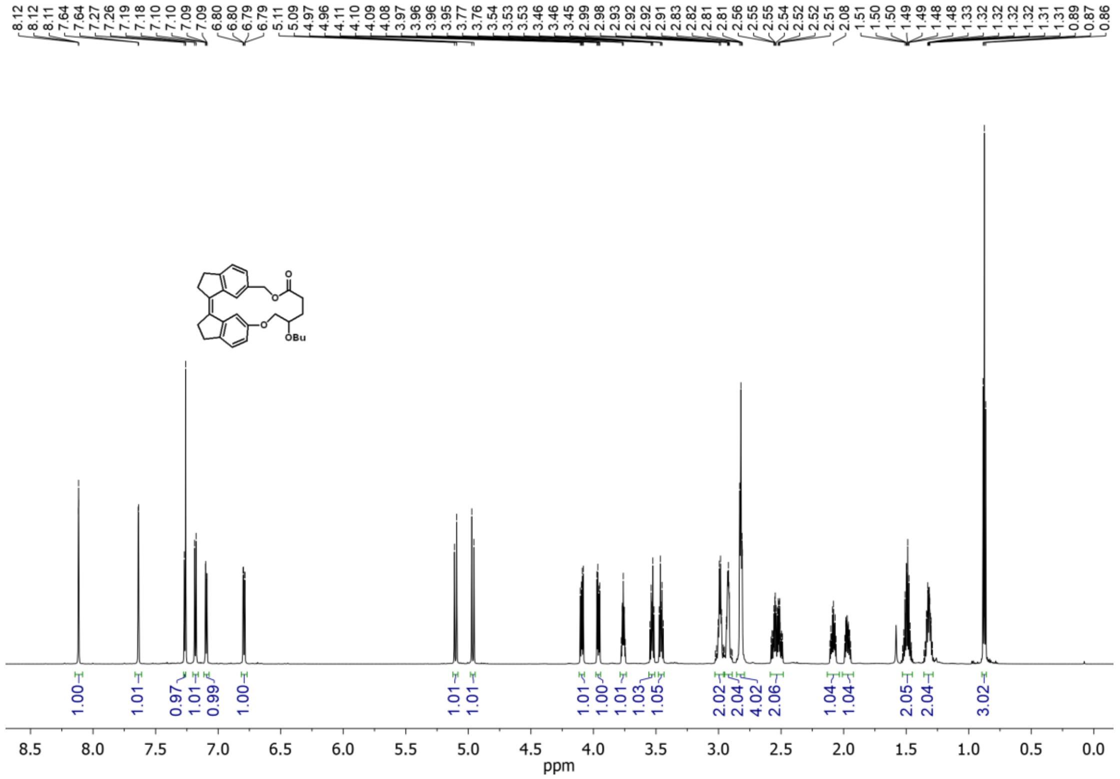

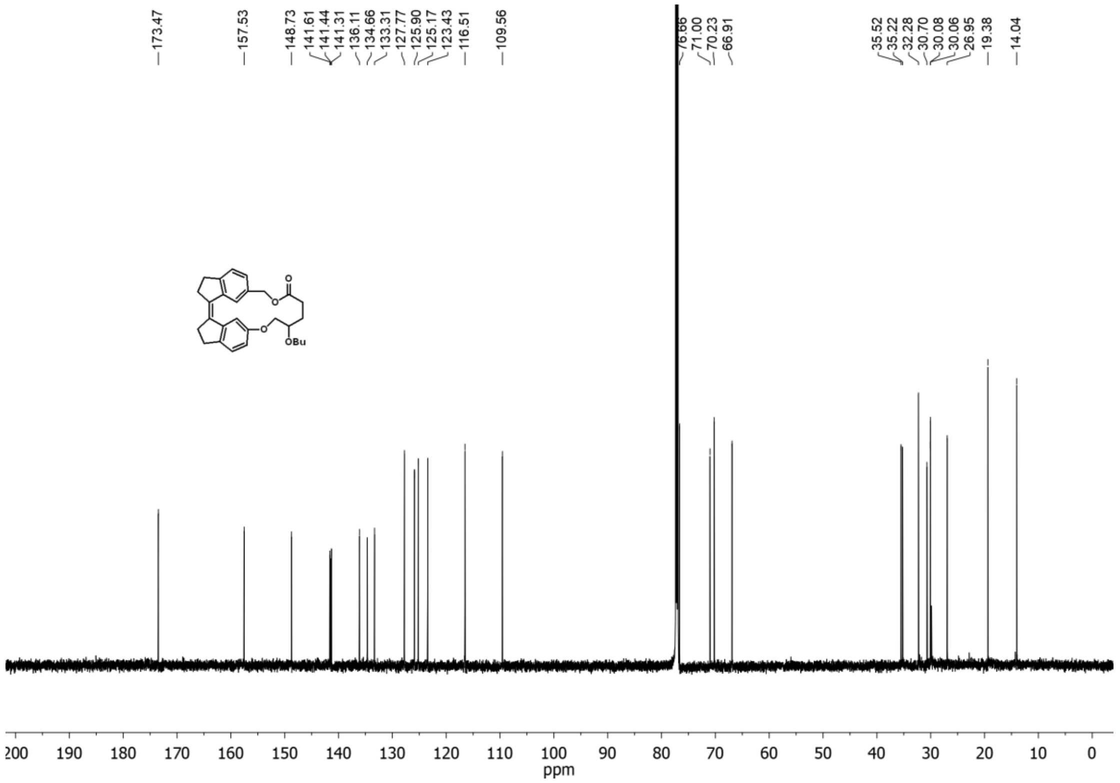

^{1}H NMR (700 MHz, CDCl$_3$) and ^{13}C NMR (126 MHz, CDCl$_3$) spectra for M5[9]

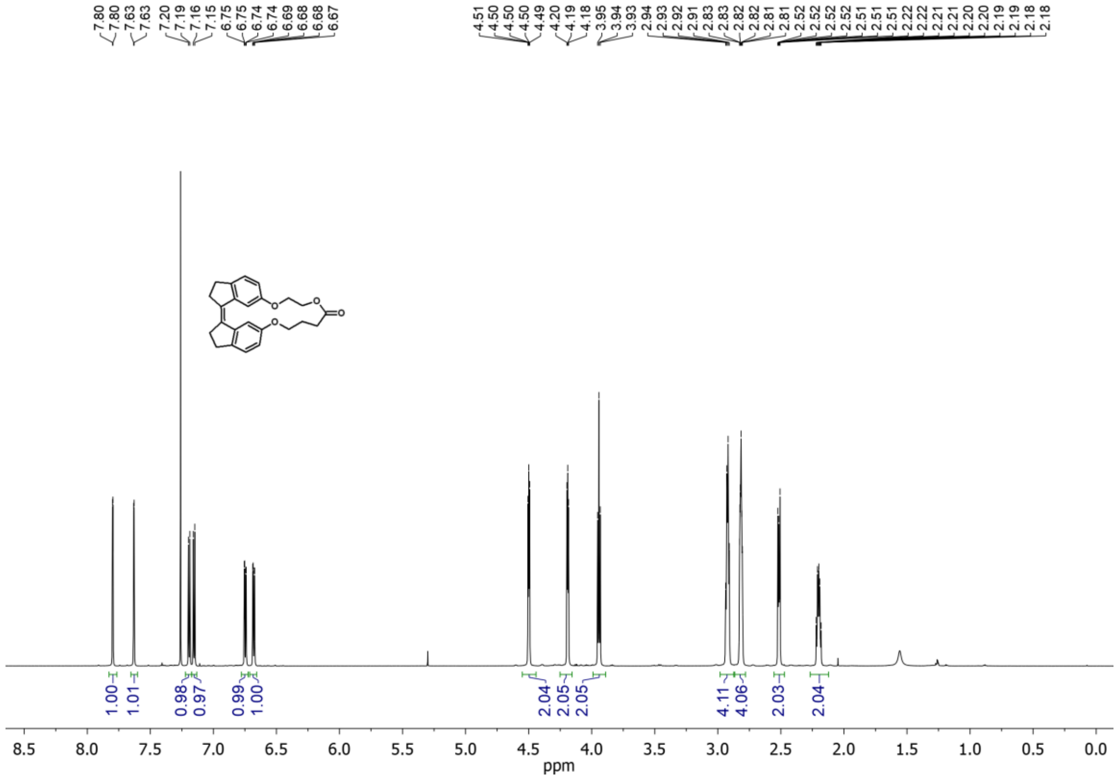

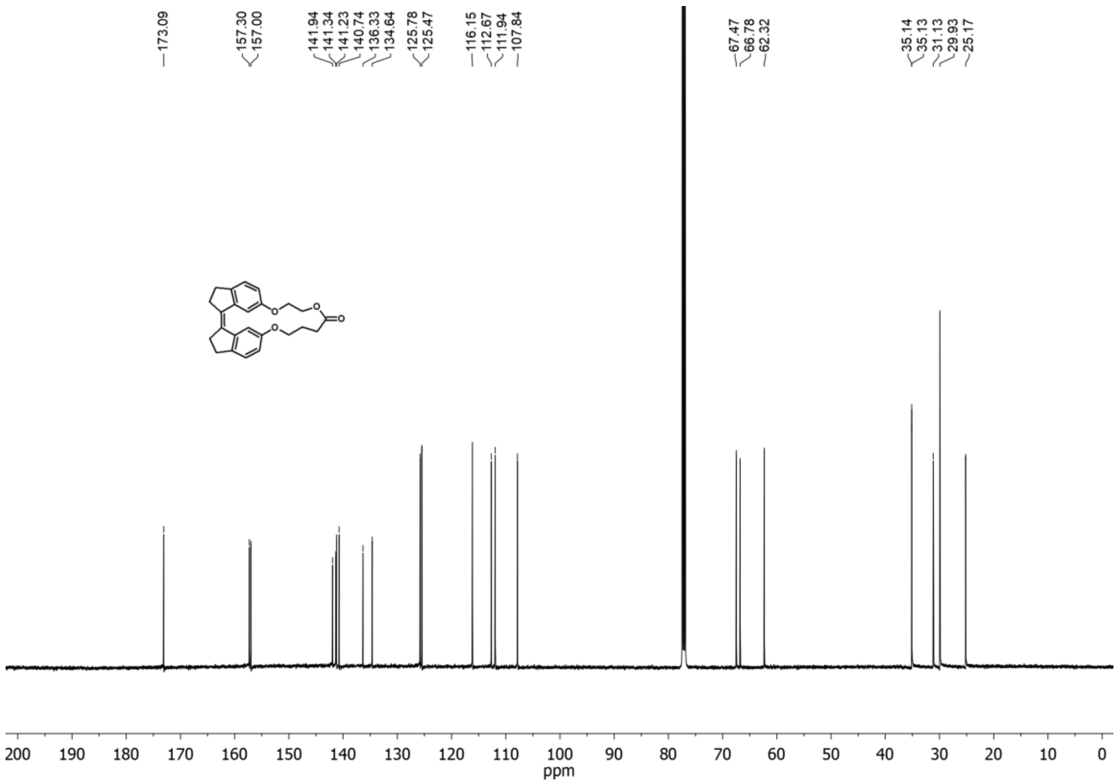

^{1}H NMR (500 MHz, $CDCl_3$) and ^{13}C NMR (176 MHz, $CDCl_3$) spectra for M6[11]

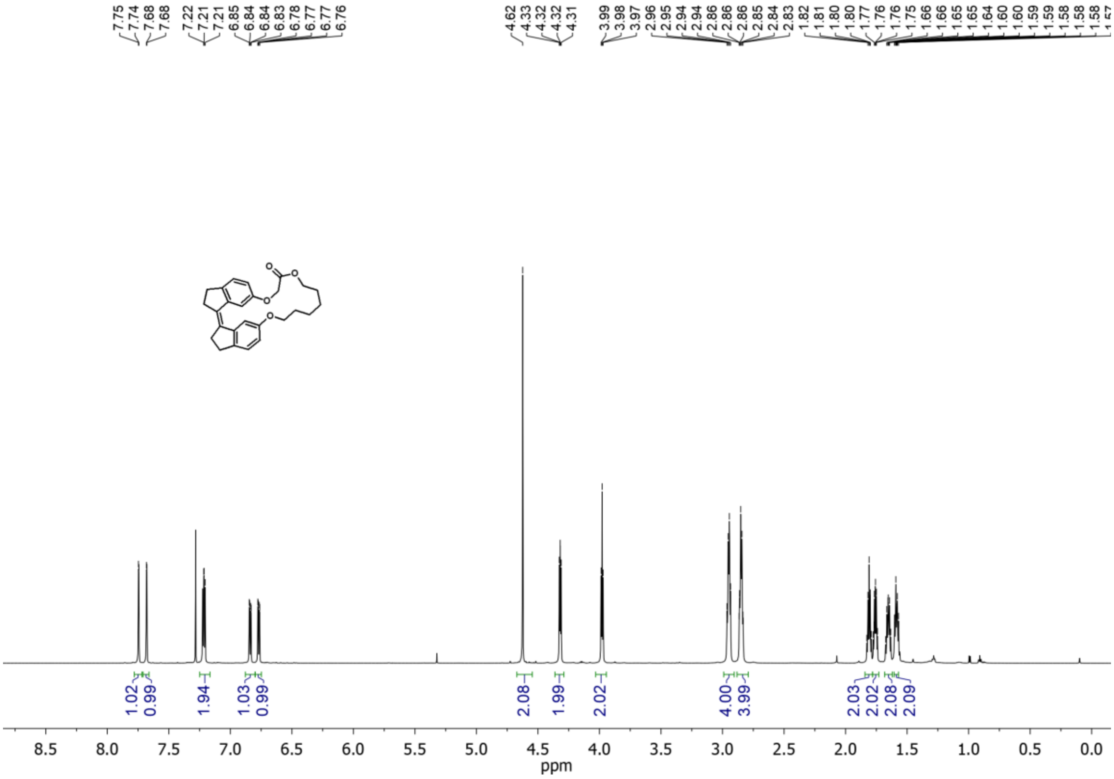

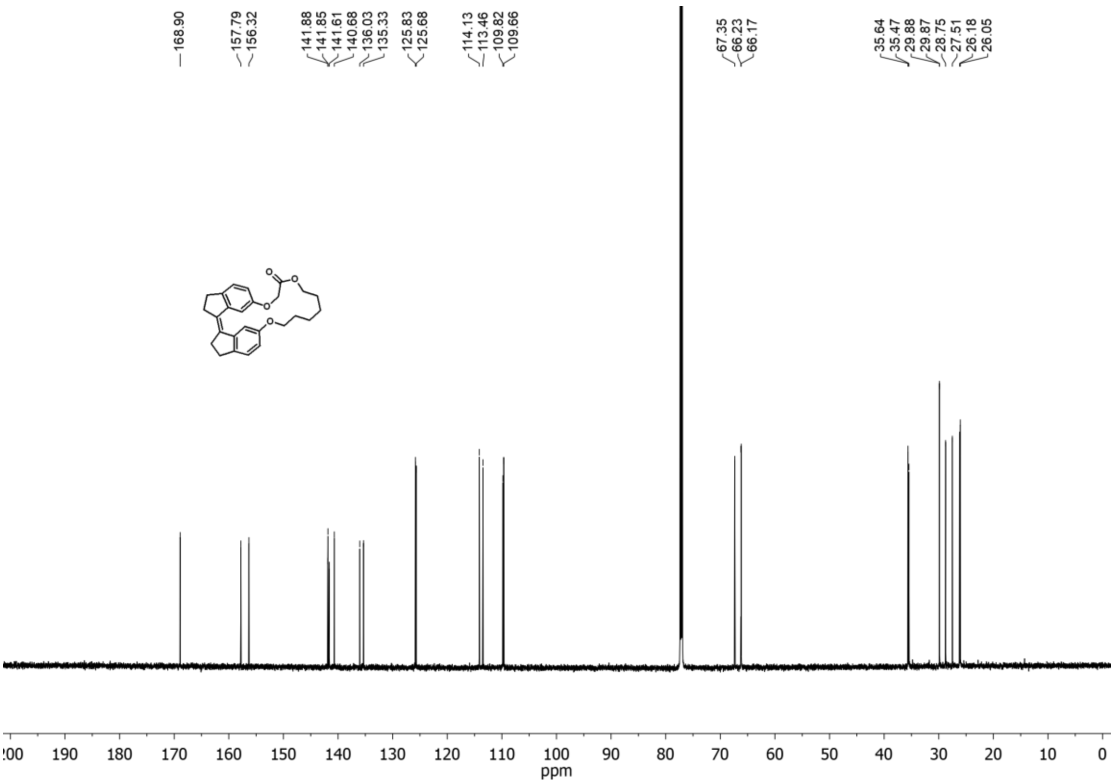

^{1}H NMR (500 MHz, $CDCl_3$) and ^{13}C NMR (126 MHz, $CDCl_3$) spectra for *E*-M6[11]

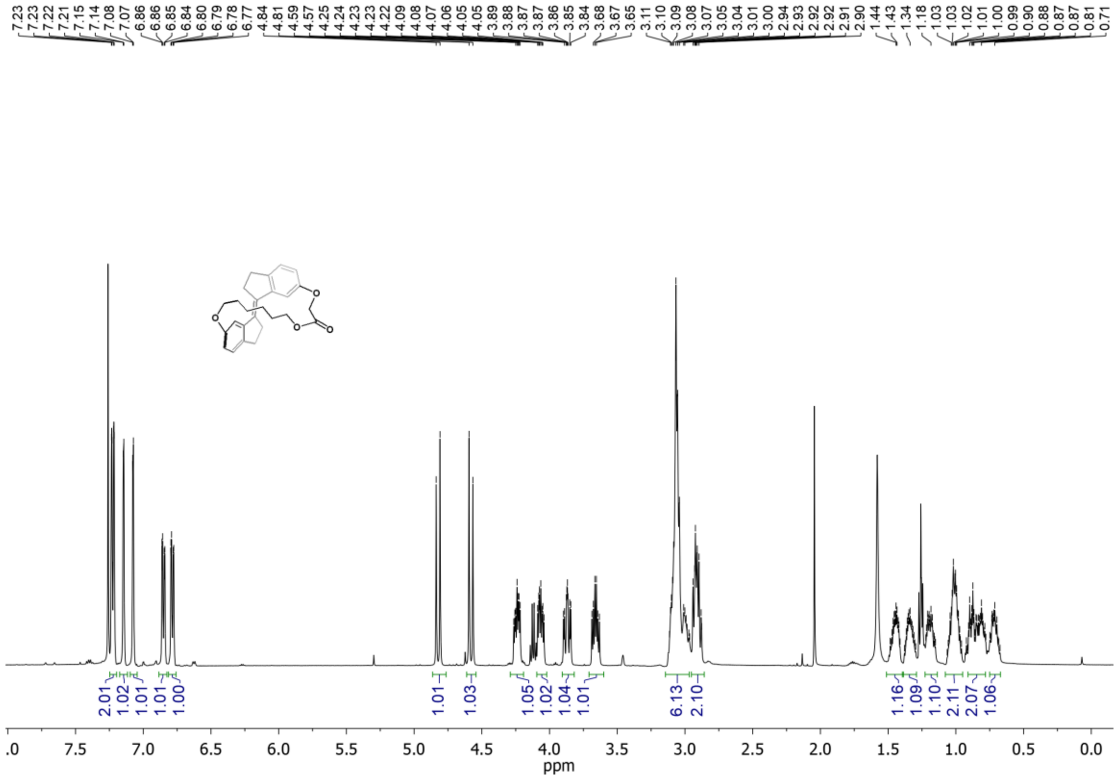

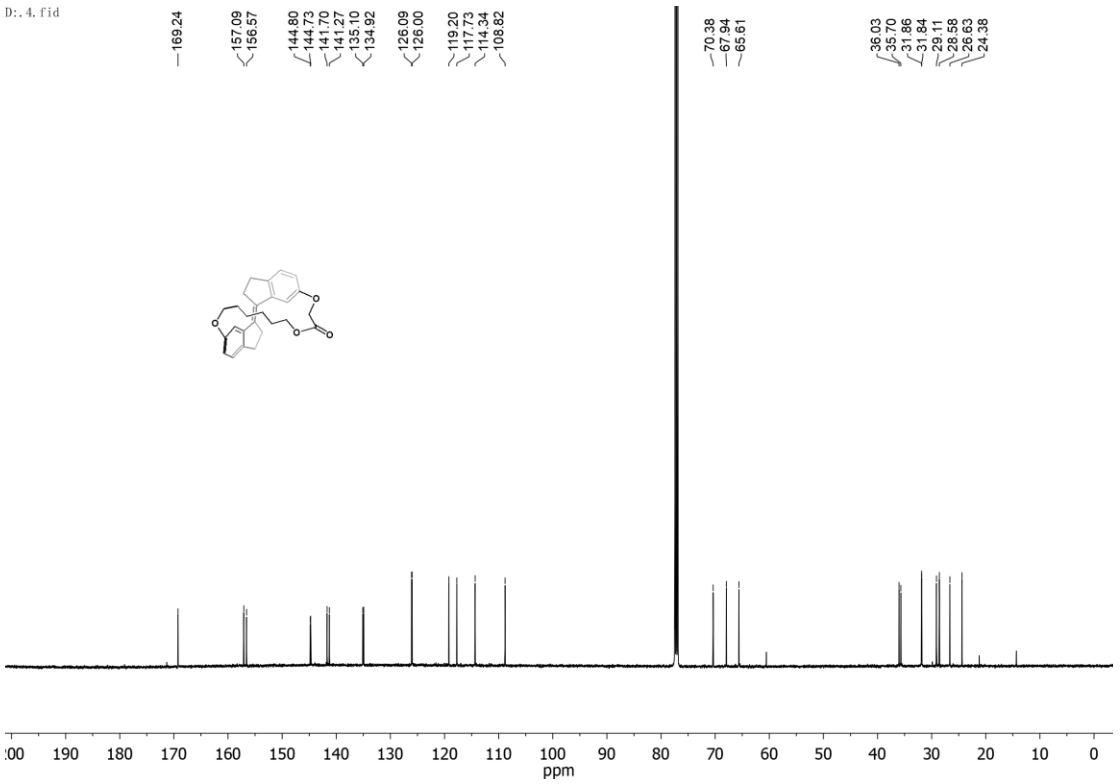

^{1}H NMR (400 MHz, CDCl$_3$) and ^{13}C NMR (126 MHz, CDCl$_3$) spectra for (3-oxo-2,3-dihydro-1H-inden-5-yl)methyl 2-(3-oxo-2,3-dihydro-1*H*-inden-5-yl)acetate

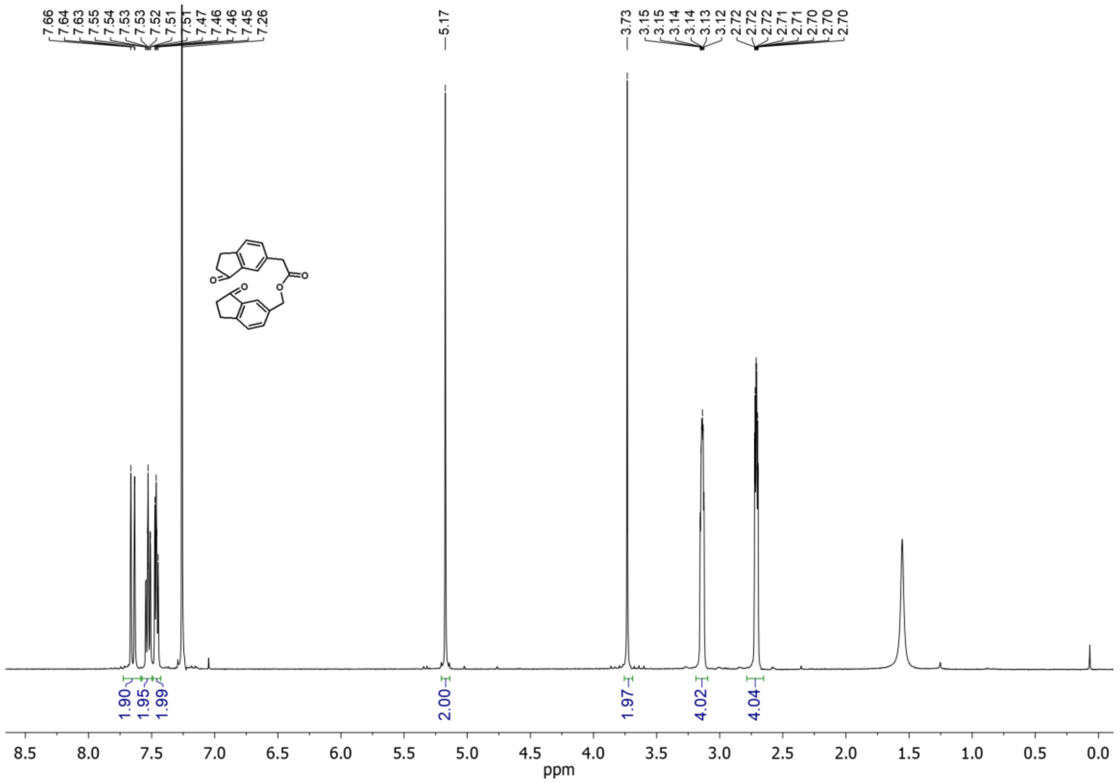

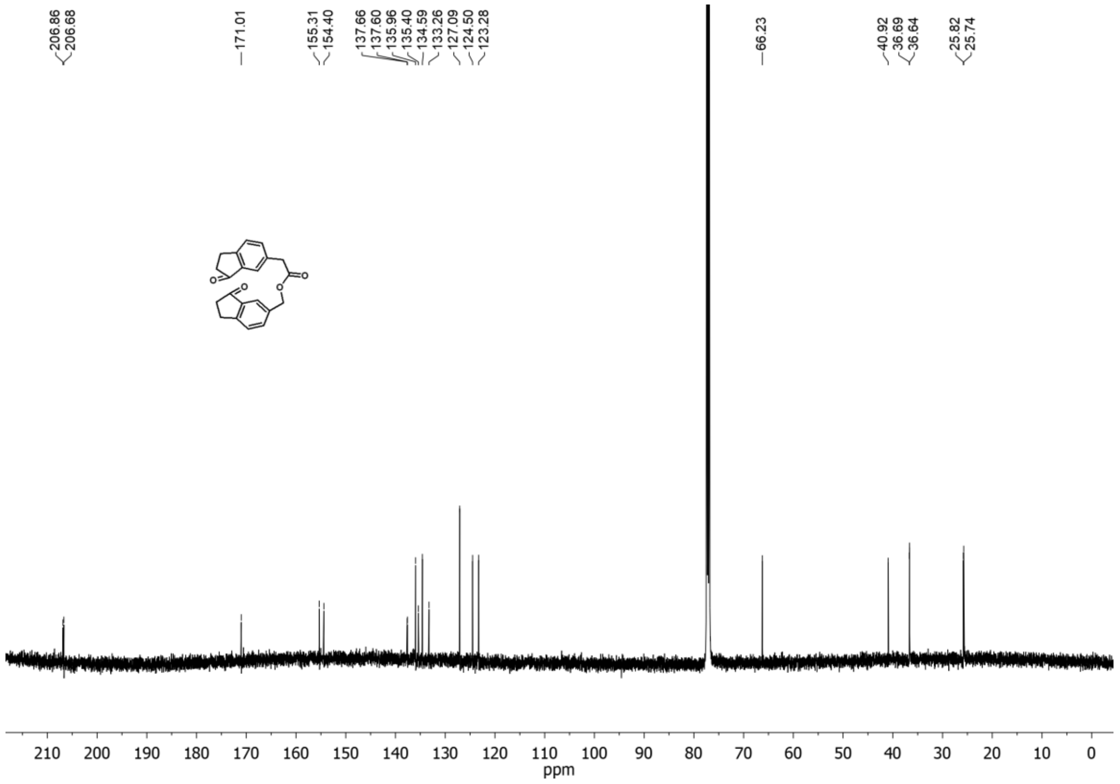

^{1}H NMR (700 MHz, CDCl$_3$) and ^{13}C NMR (126 MHz, CDCl$_3$) spectra for (3-oxo-2,3-dihydro-1H-inden-5-yl)methyl 2-((3-oxo-2,3-dihydro-1*H*-inden-5-yl)oxy)acetate

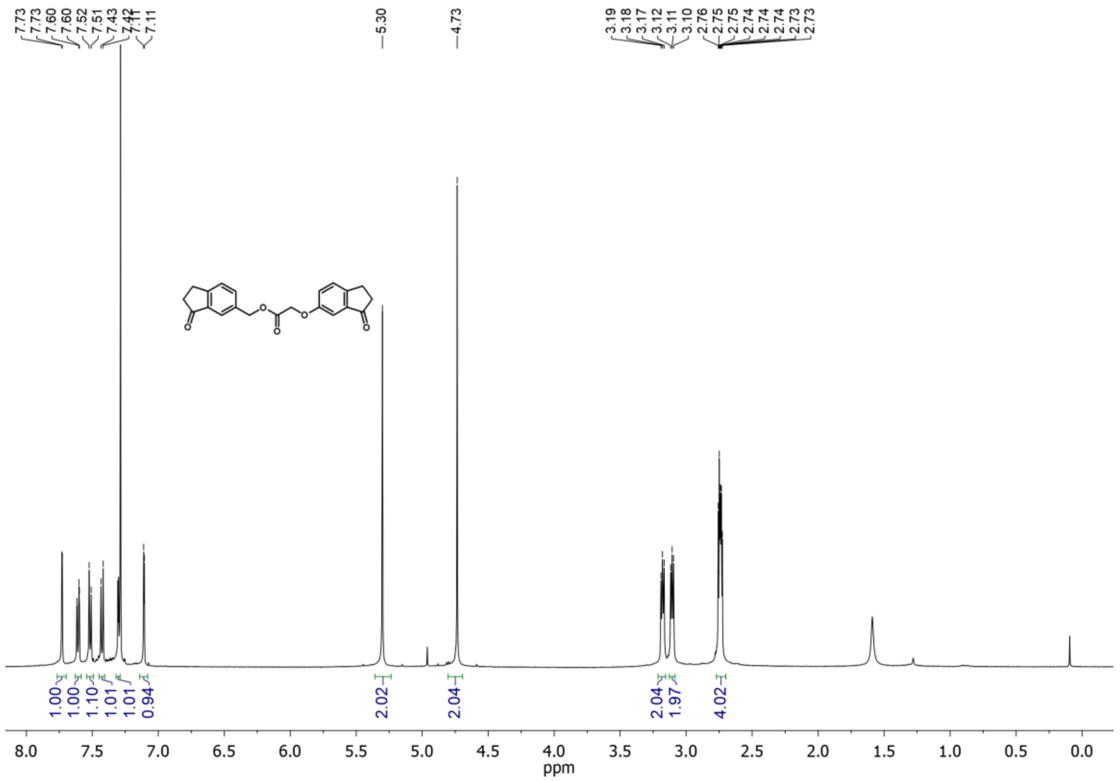

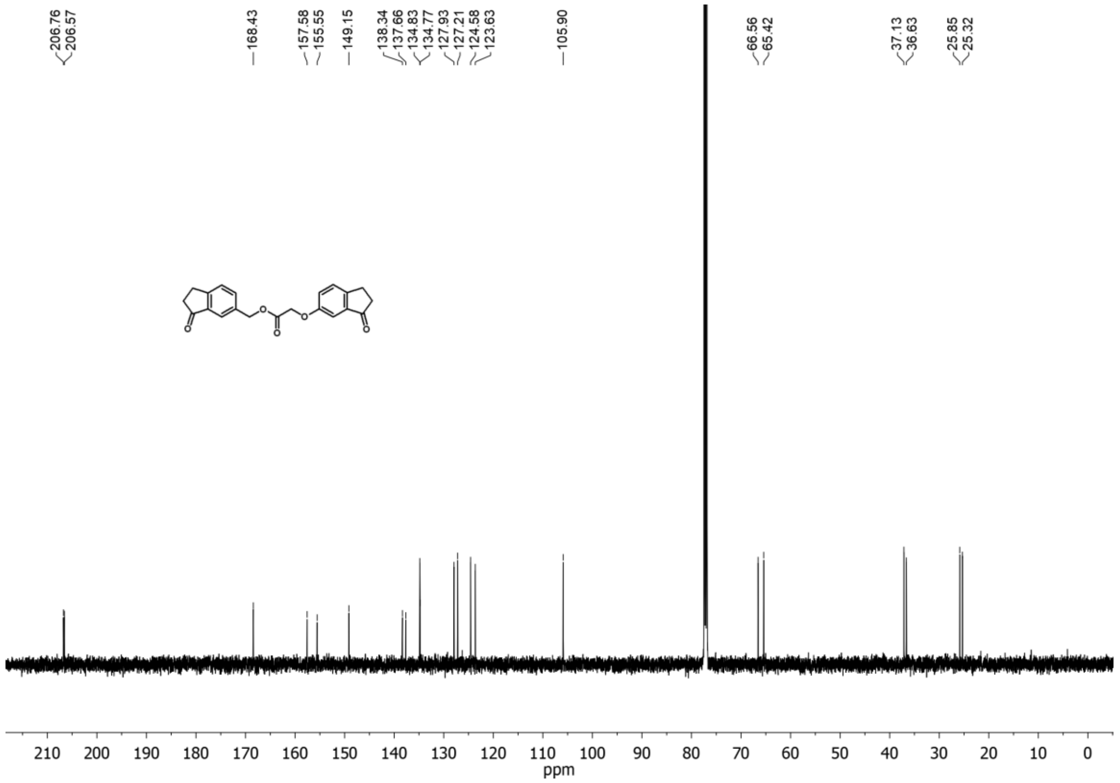

^{1}H NMR (500 MHz, $CDCl_3$) and ^{13}C NMR (126 MHz, $CDCl_3$) spectra for (3-oxo-2,3-dihydro-1*H*-inden-5-yl)methyl 5-bromopentanoate

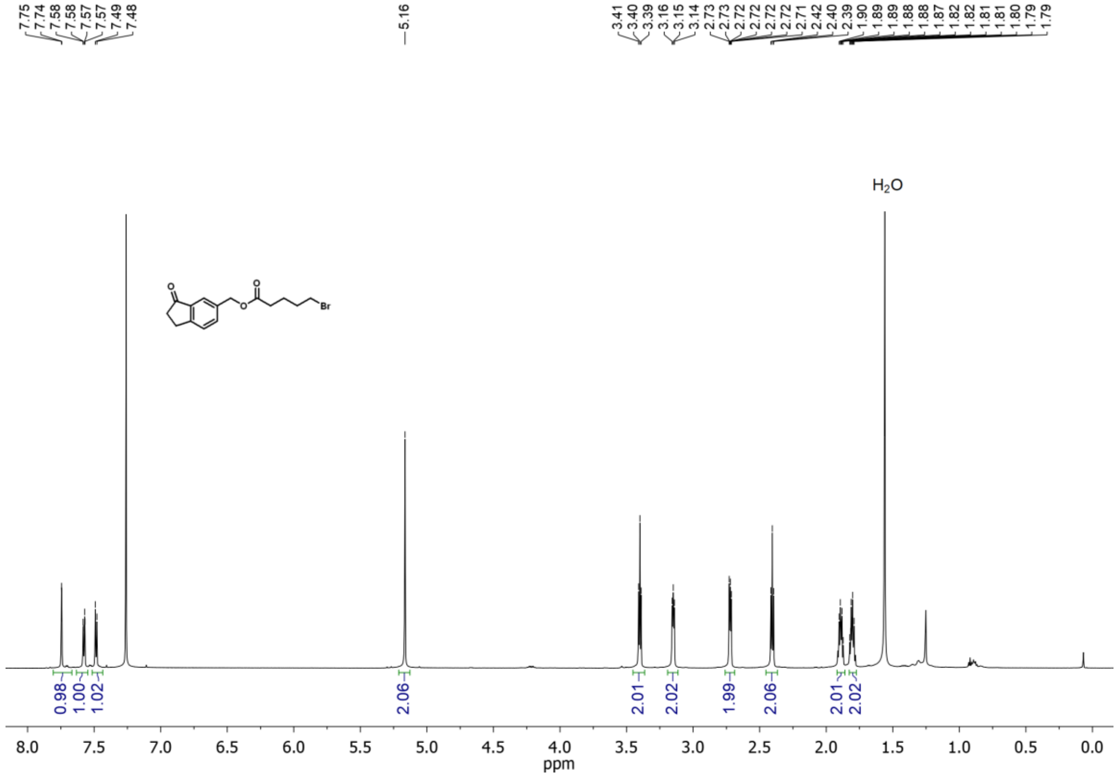

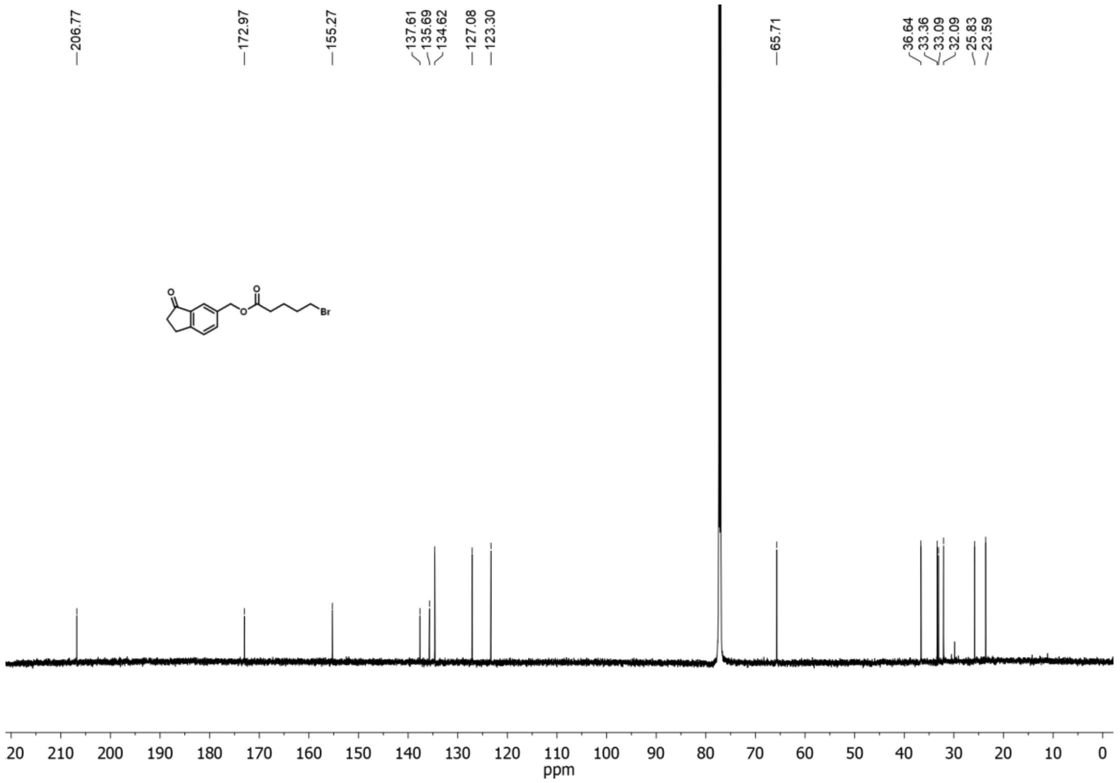

^{1}H NMR (500 MHz, CDCl$_3$) and ^{13}C NMR (126 MHz, CDCl$_3$) spectra for (3-oxo-2,3-dihydro-1*H*-inden-5-yl)methyl 5-((3-oxo-2,3-dihydro-1*H*-inden-5-yl)oxy)pentano- ate

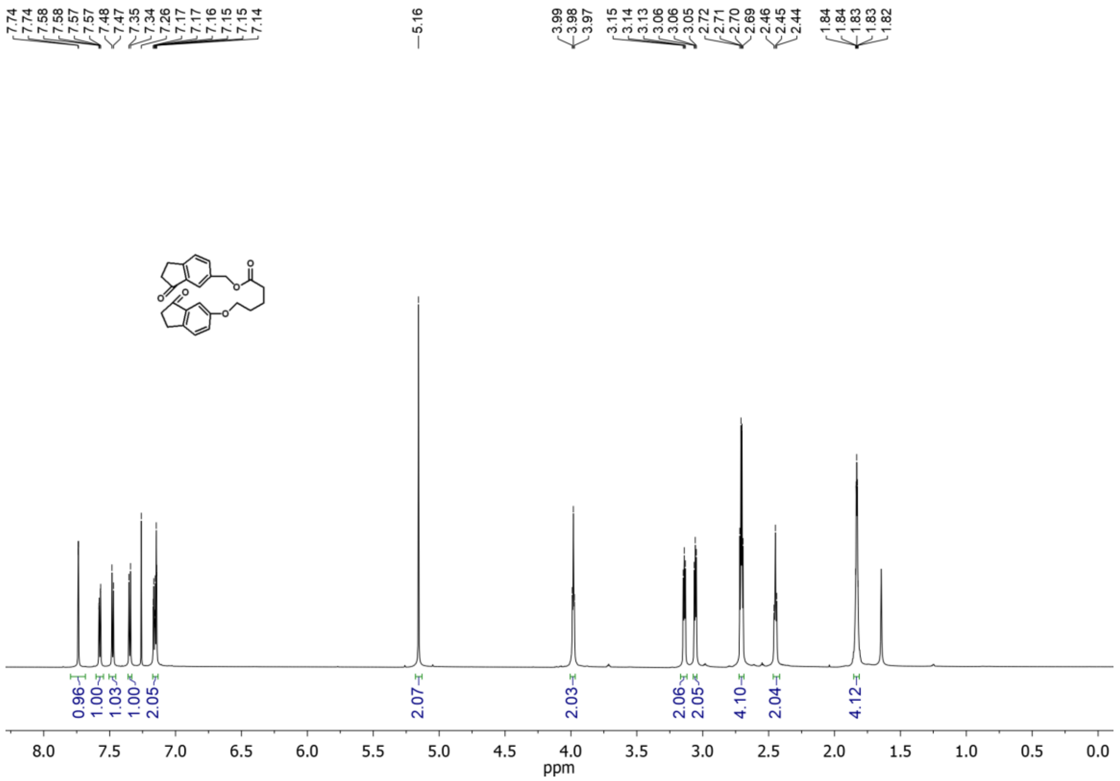

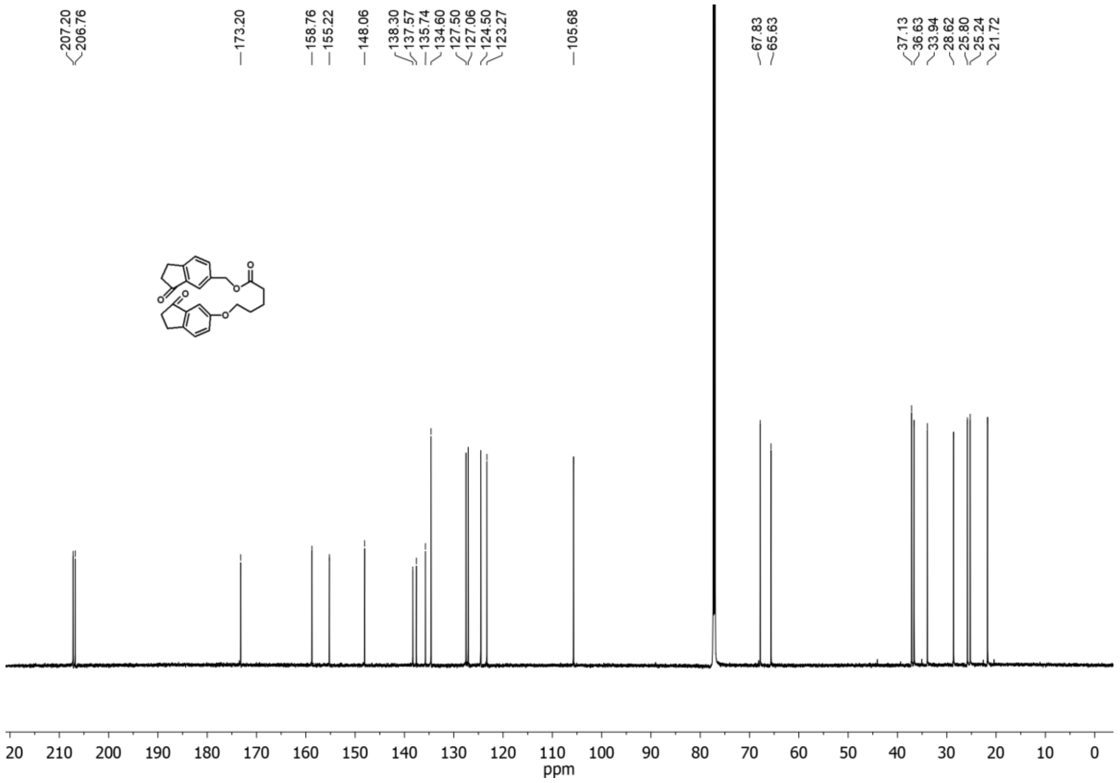

^{1}H NMR (700 MHz, $CDCl_3$) and ^{13}C NMR (176 MHz, $CDCl_3$) spectra for benzyl 4-butoxy-5-((3-oxo-2,3-dihydro-1*H*-inden-5-yl)oxy)pentanoate

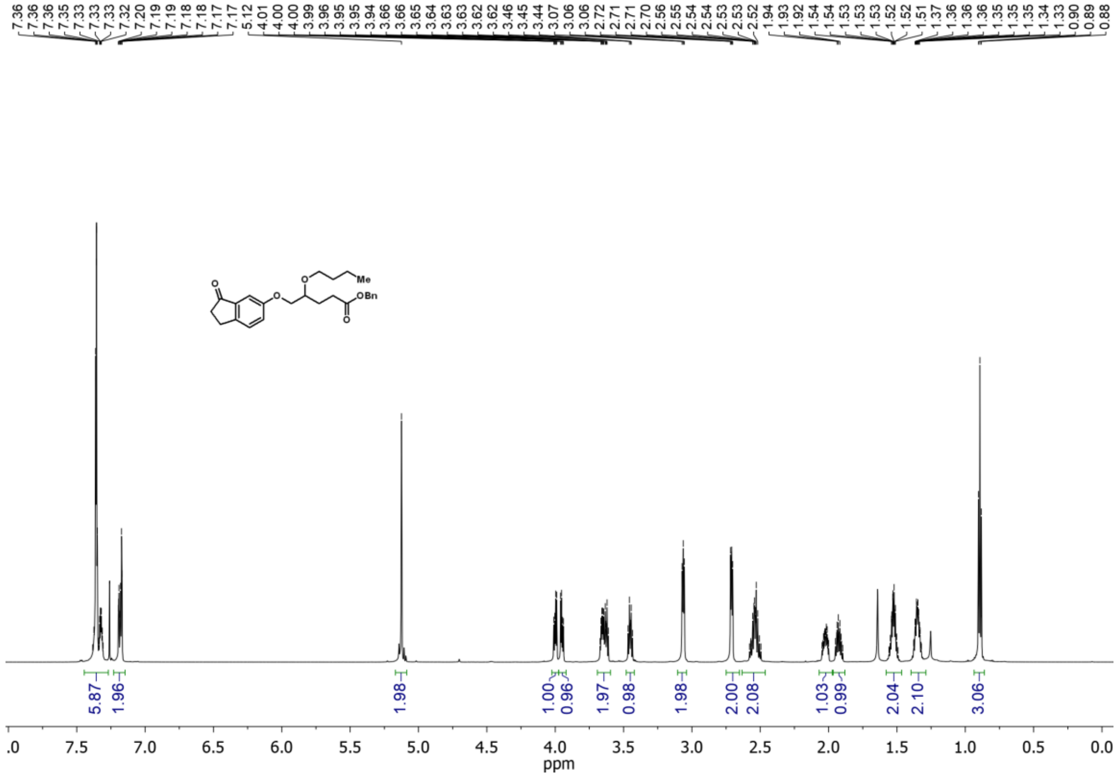

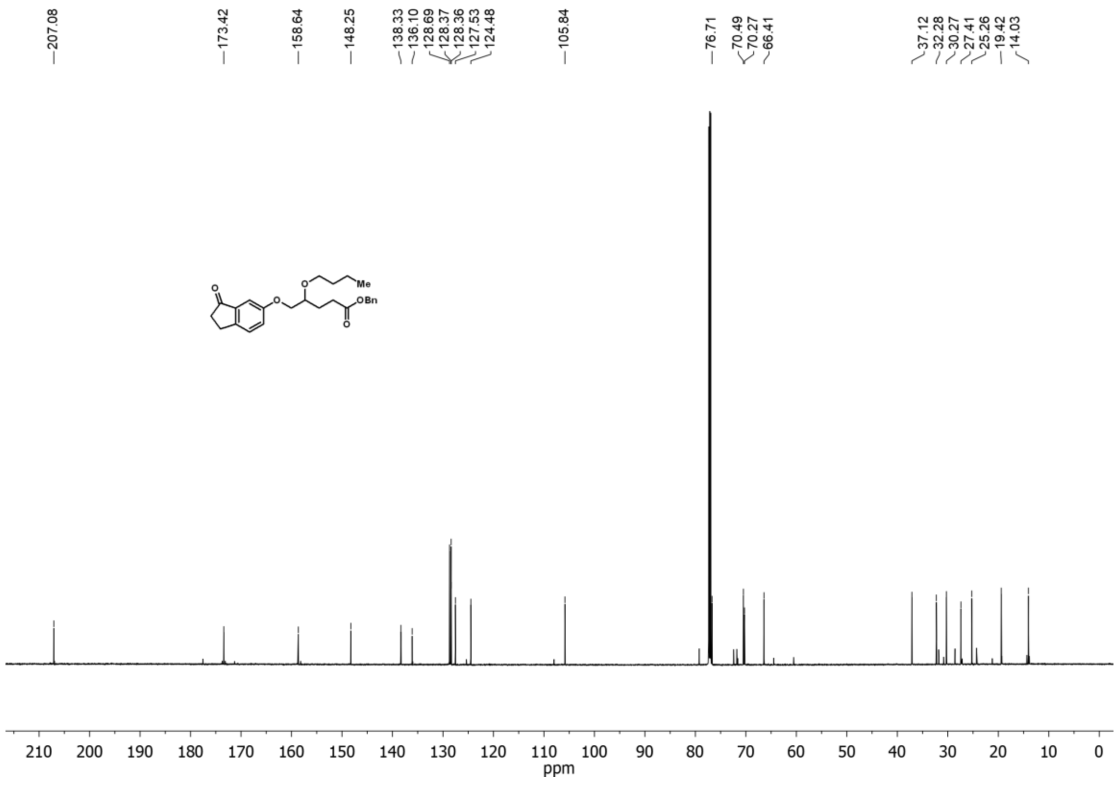

^{1}H NMR (700 MHz, $CDCl_3$) and ^{13}C NMR (176 MHz, $CDCl_3$) spectra for (3-oxo-2,3-dihydro-1H-inden-5-yl)methyl 4-butoxy-5-((3-oxo-2,3-dihydro-1H-inden-5-yl)oxy)pentanoate

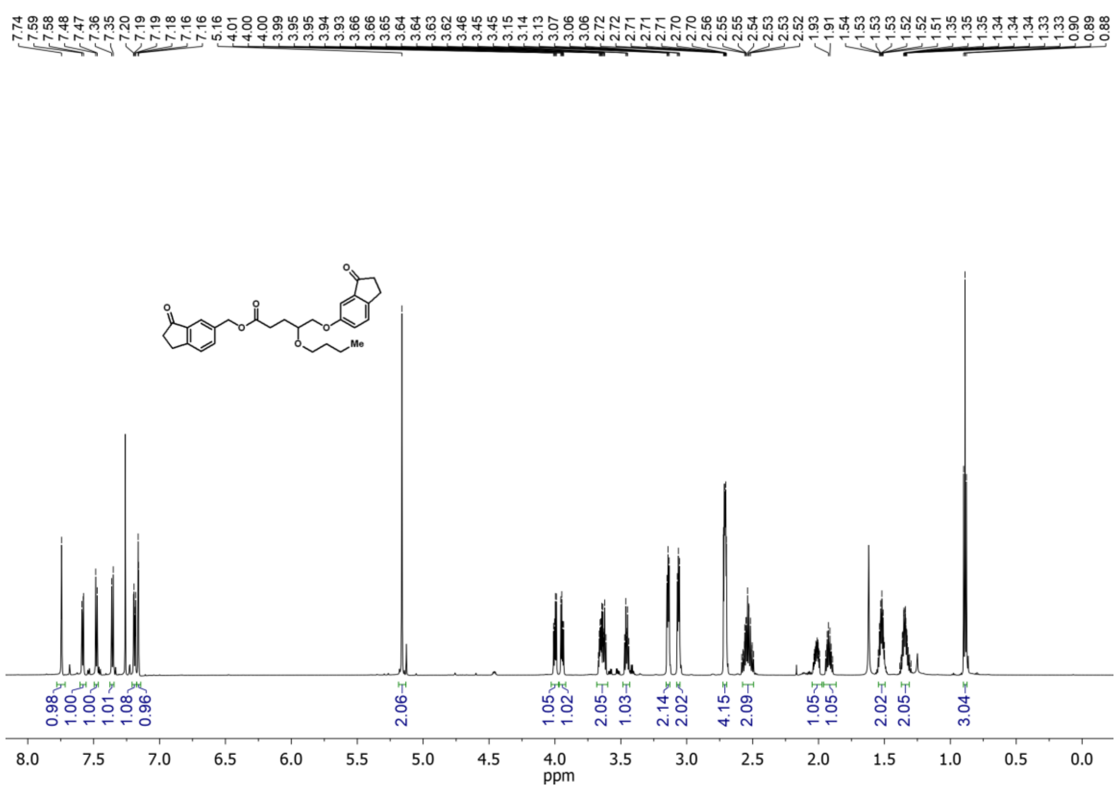

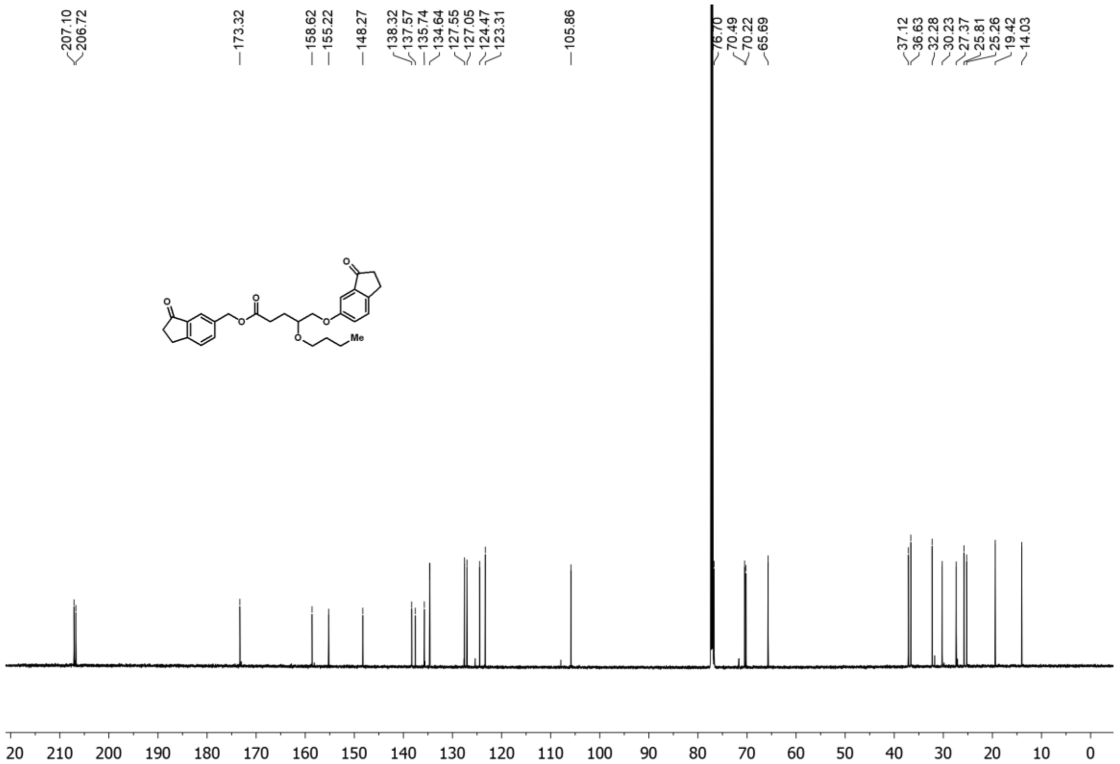

^{1}H NMR (500 MHz, CDCl3) and ^{13}C NMR (126 MHz, CDCl3) spectra for 2-((3-oxo-2,3-dihydro-1*H*-inden-5-yl)oxy)ethyl 4-((3-oxo-2,3-dihydro-1*H*-inden-5-yl)oxy)butanoate

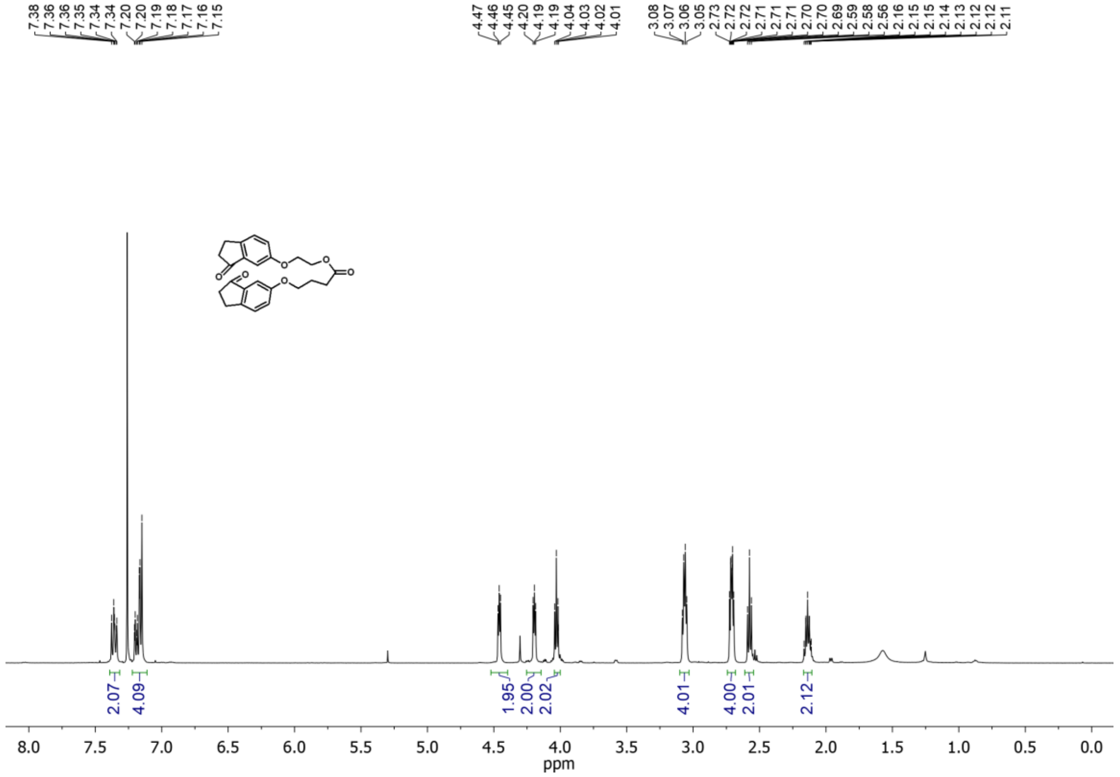

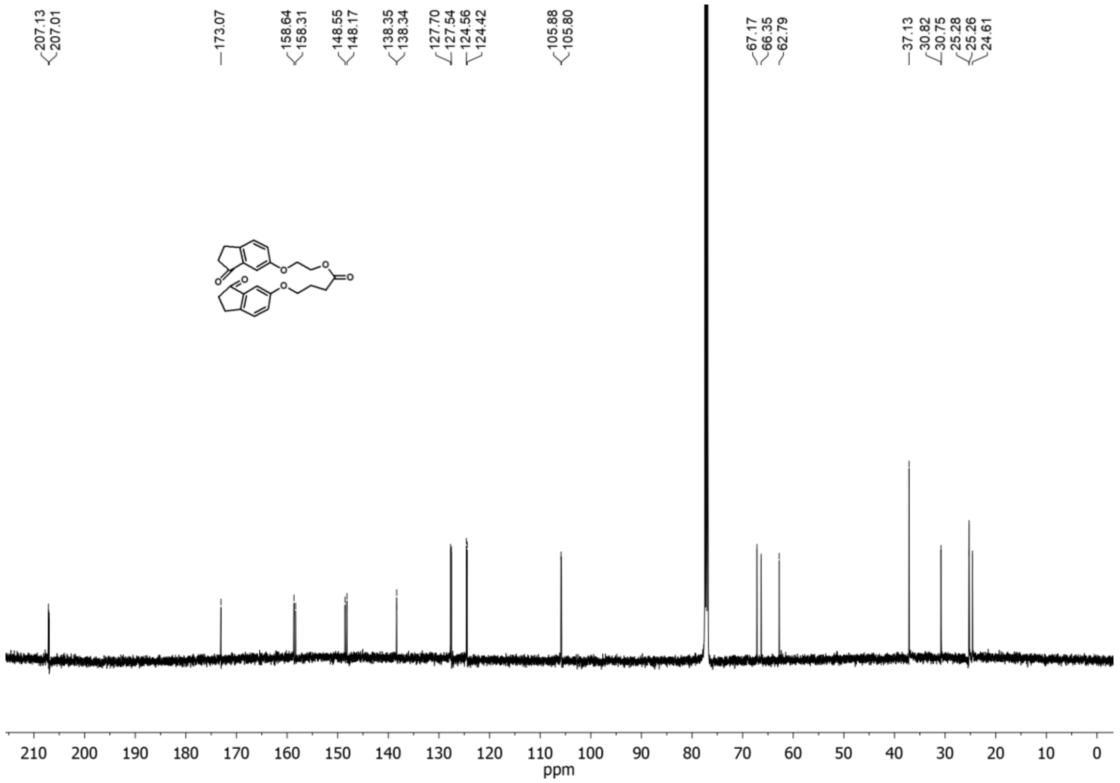

^{1}H NMR (500 MHz, CDCl3) and ^{13}C NMR (126 MHz, CDCl3) spectra for 6-((3-oxo-2,3-

dihydro-1*H*-inden-5-yl)oxy)hexyl 2-((3-oxo-2,3-dihydro-1*H*-inden-5-yl)oxy)acetate

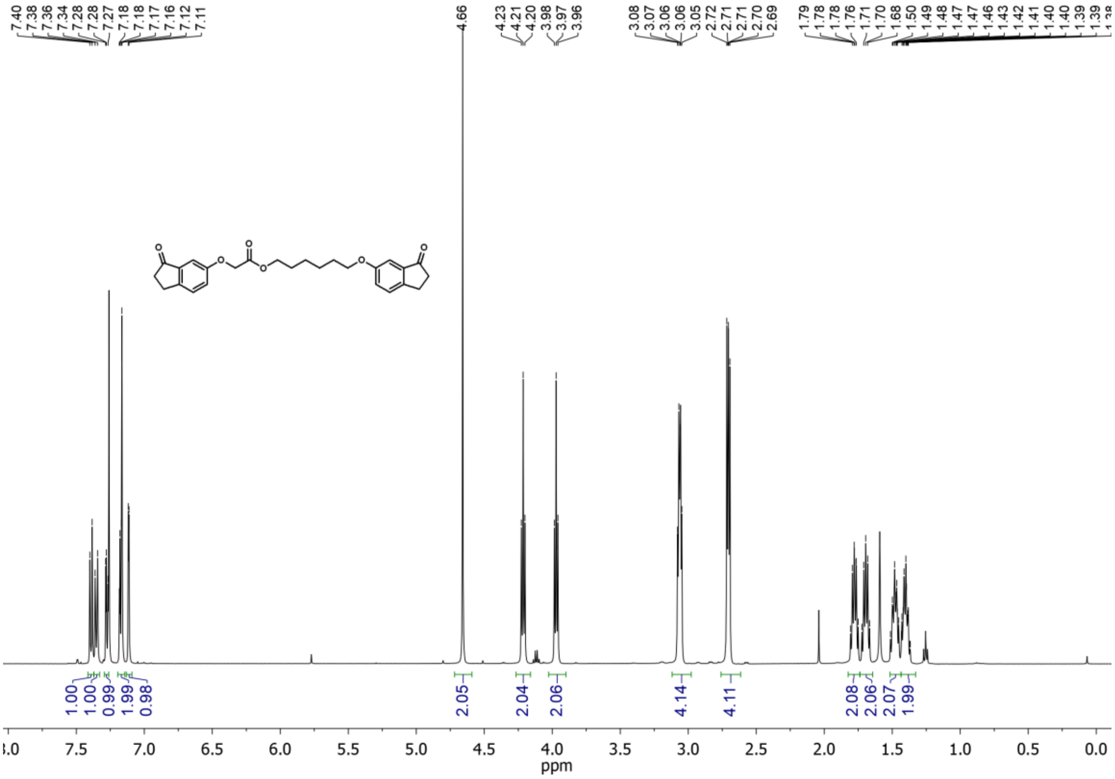

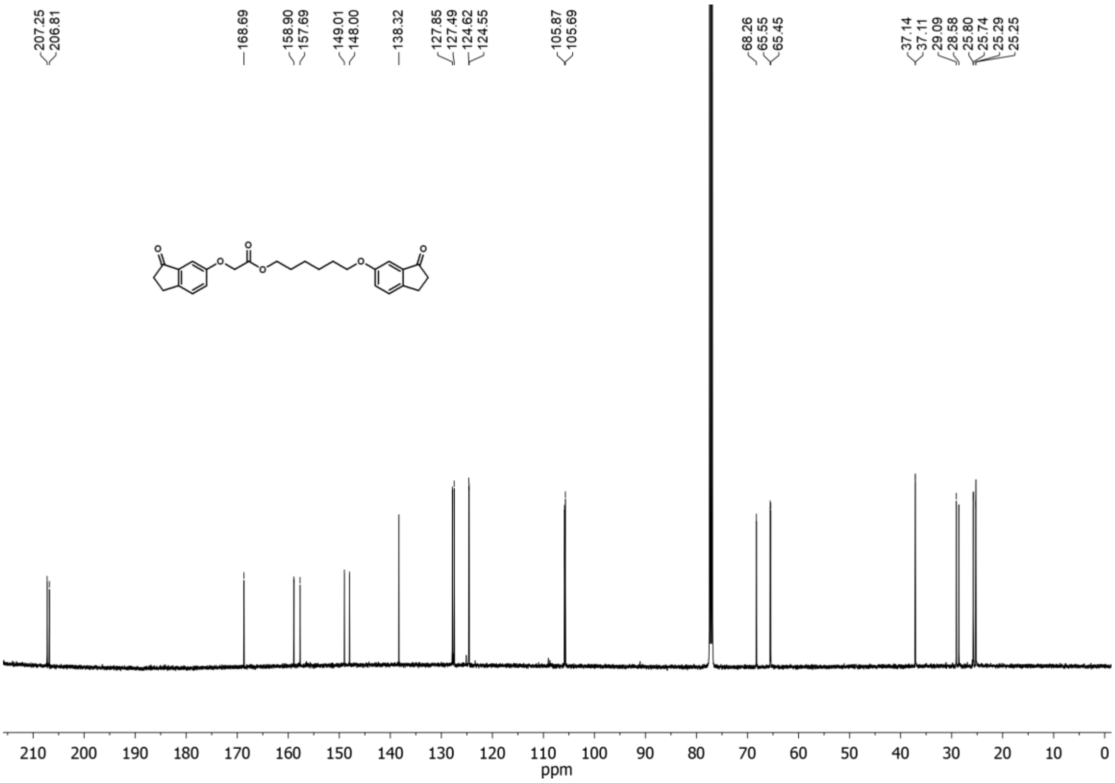

^{1}H NMR (500 MHz, $CDCl_3$) and ^{13}C NMR (126 MHz, $CDCl_3$) spectra for benzyl 4-butoxy-5-hydroxypentanoate

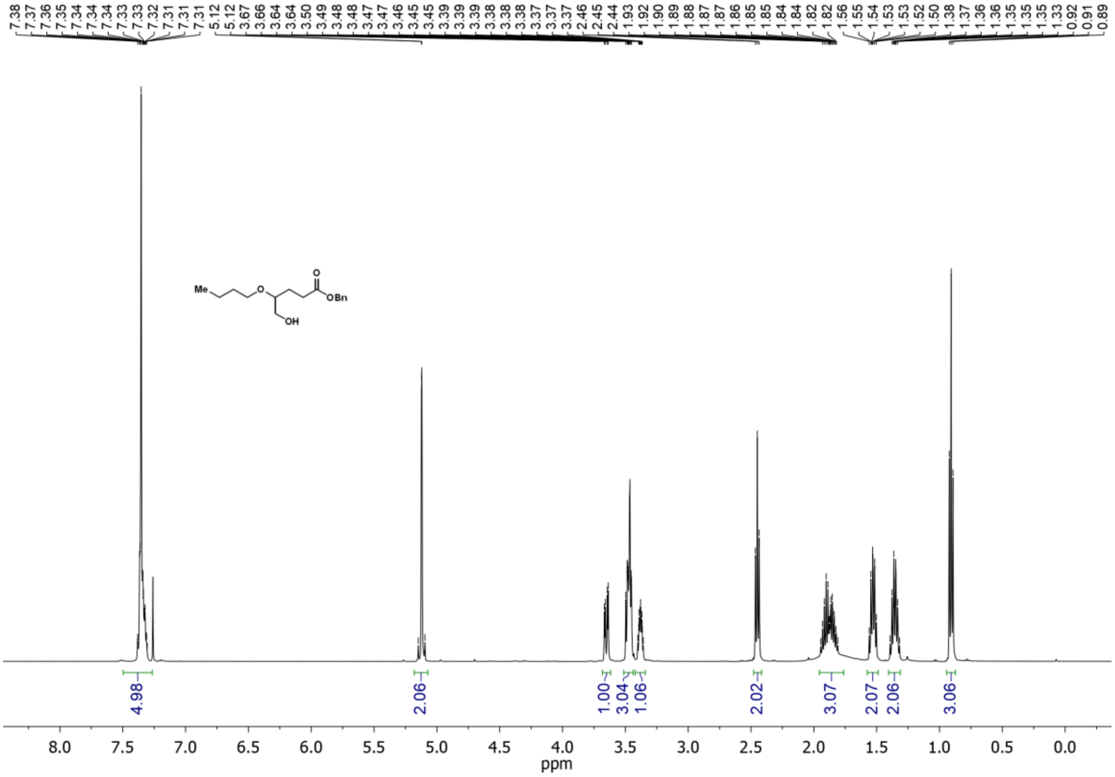

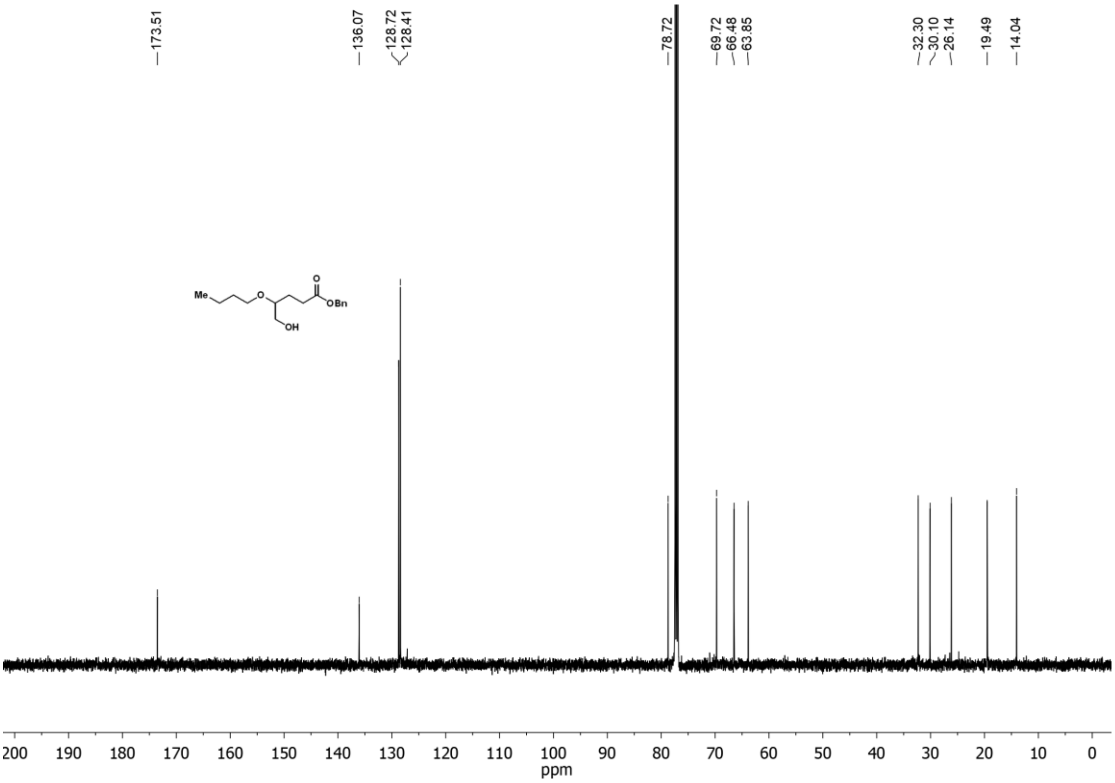

^{1}H NMR (500 MHz, $CDCl_3$) spectra for P3

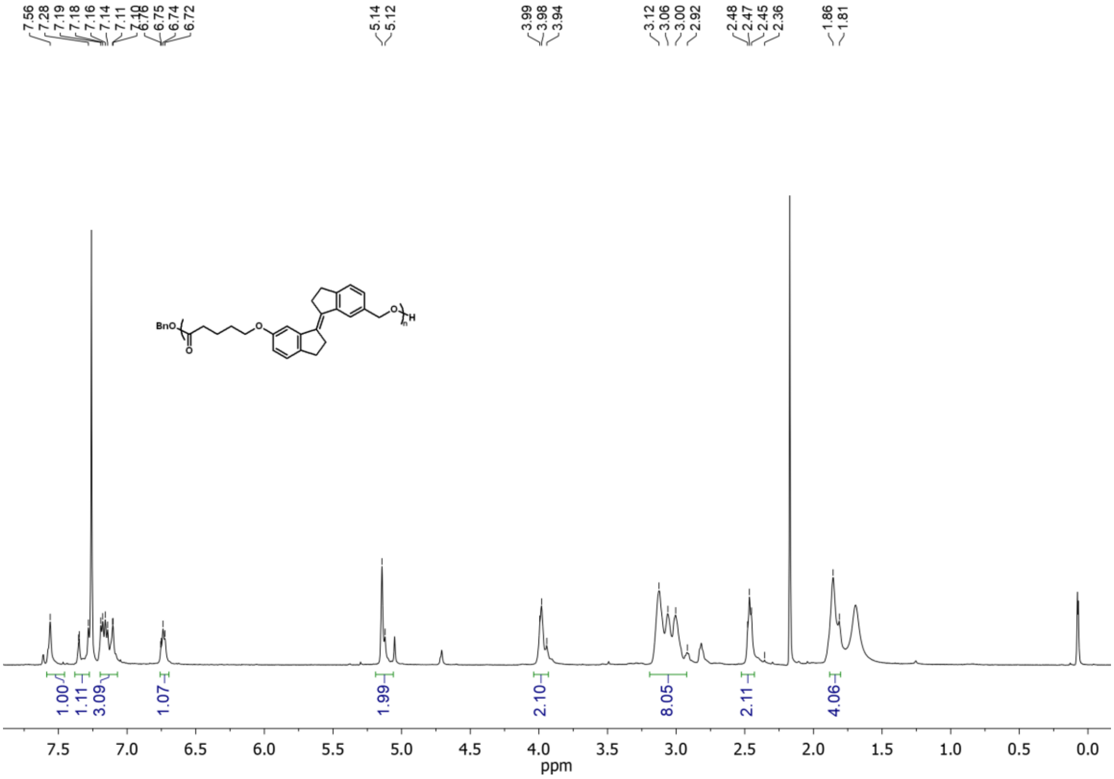

^{1}H NMR (700 MHz, $CDCl_3$) spectra for P4

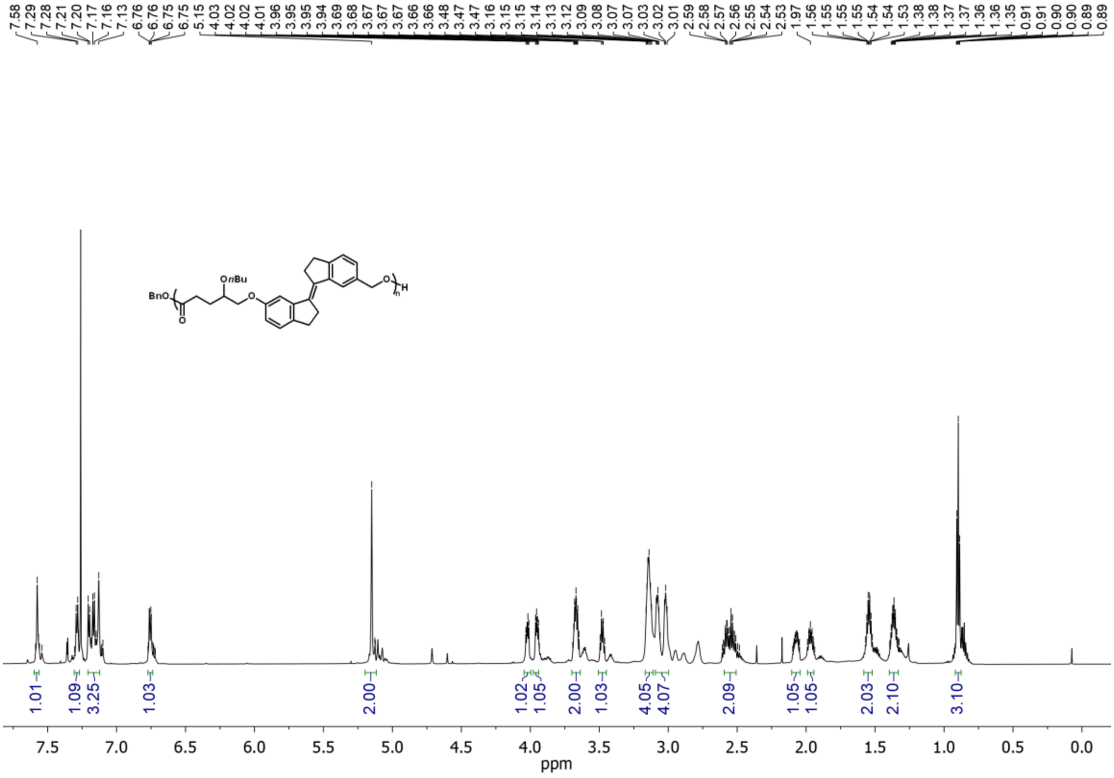

^{1}H NMR (700 MHz, $CDCl_3$) spectra for P5

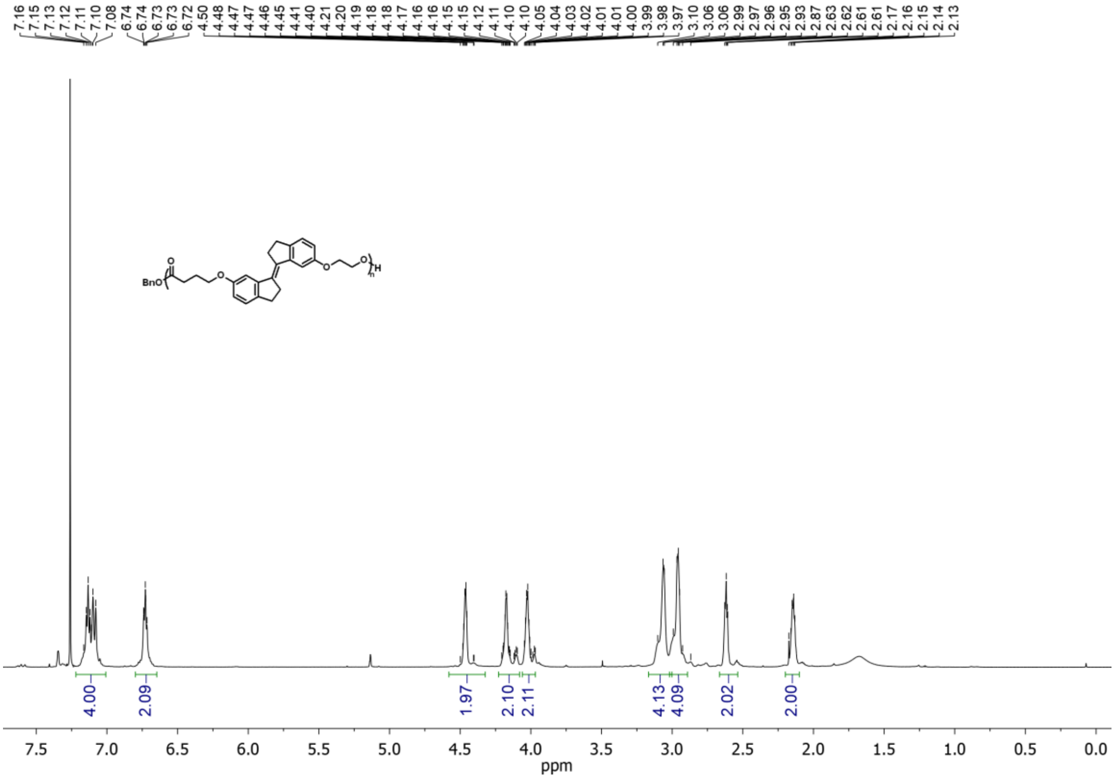

^{1}H NMR (700 MHz, $CDCl_3$) spectra for P6

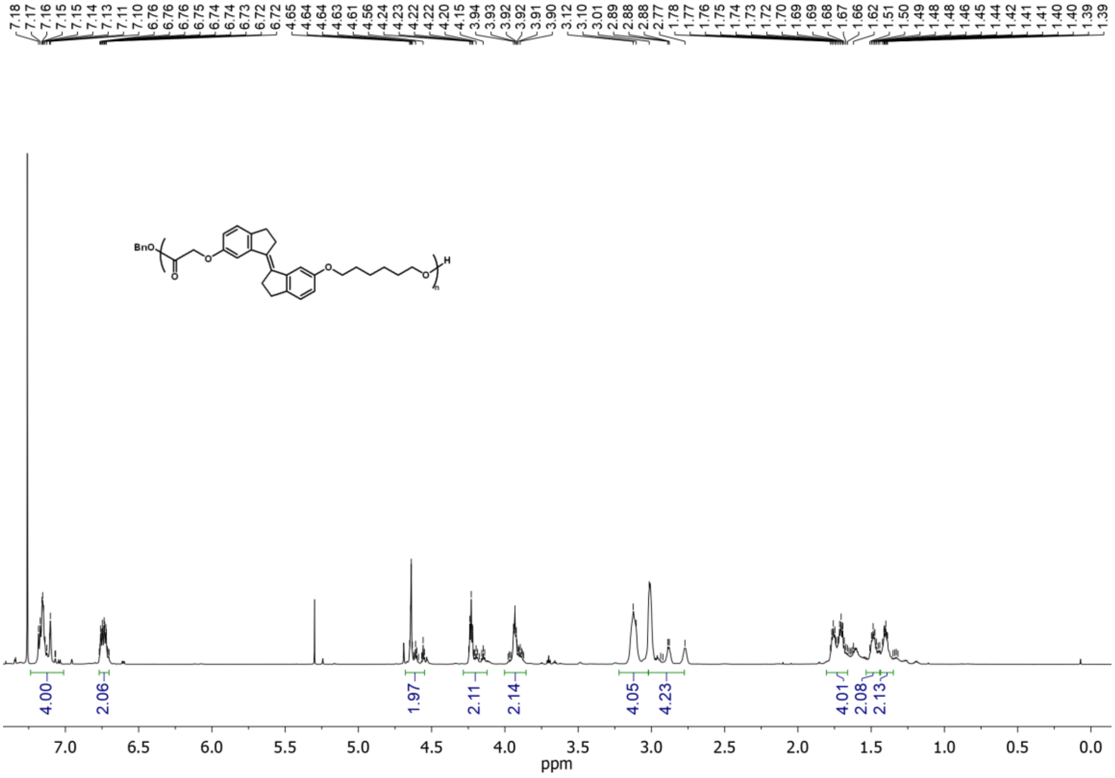

^{1}H NMR (500 MHz, CDCl$_3$) spectra for P4-co-CL

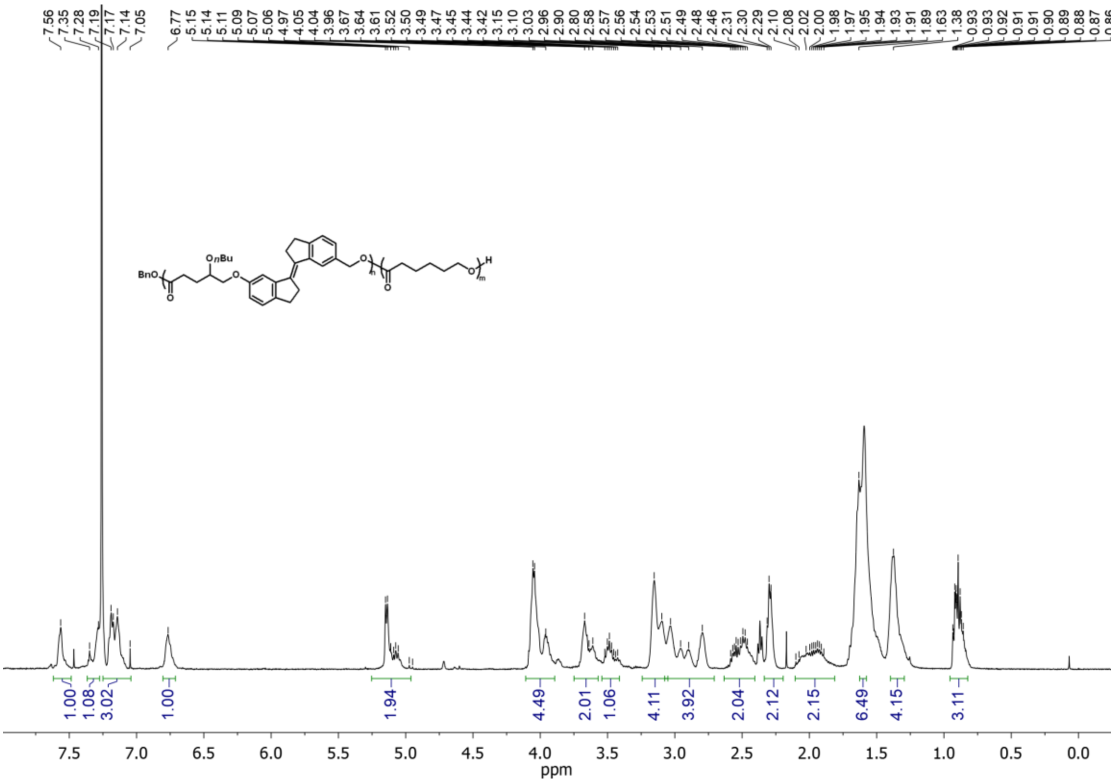

^{1}H NMR (700 MHz, CDCl$_3$) and ^{13}C NMR (176 MHz, CDCl$_3$) spectra for *E*-ROEM4[8]

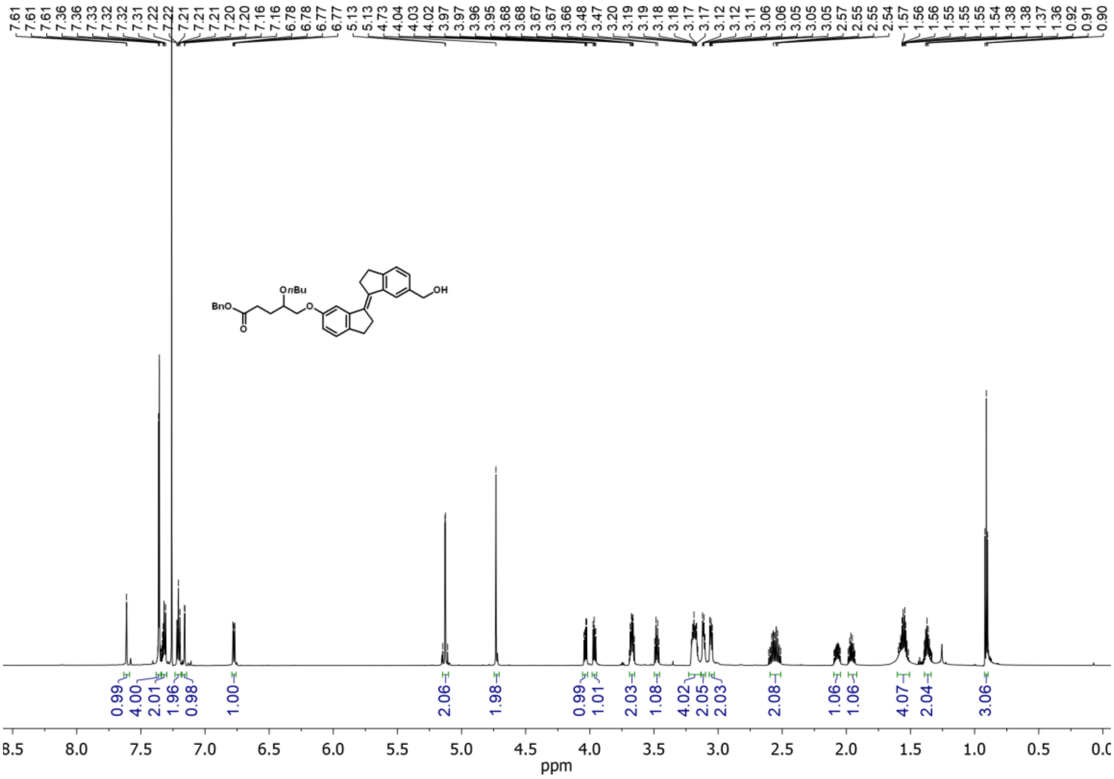

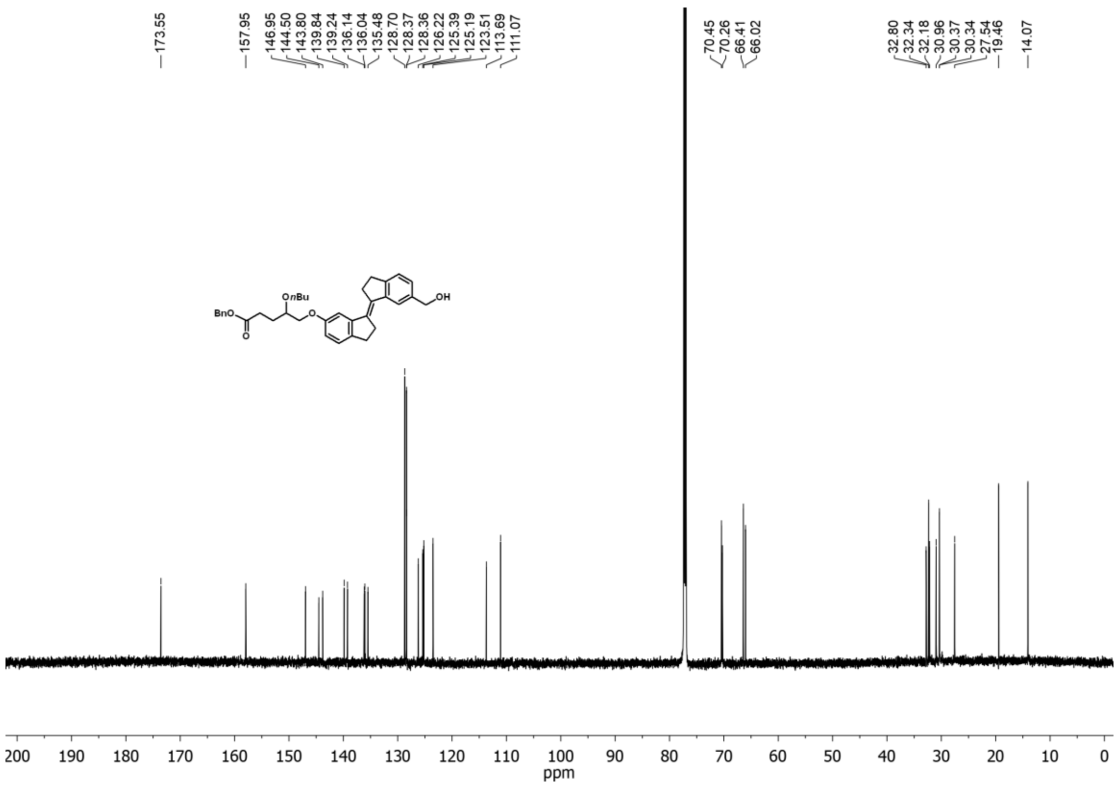

^{1}H NMR (700 MHz, $CDCl_3$) and ^{13}C NMR (176 MHz, $CDCl_3$) spectra for *Z*-ROEM4[8]

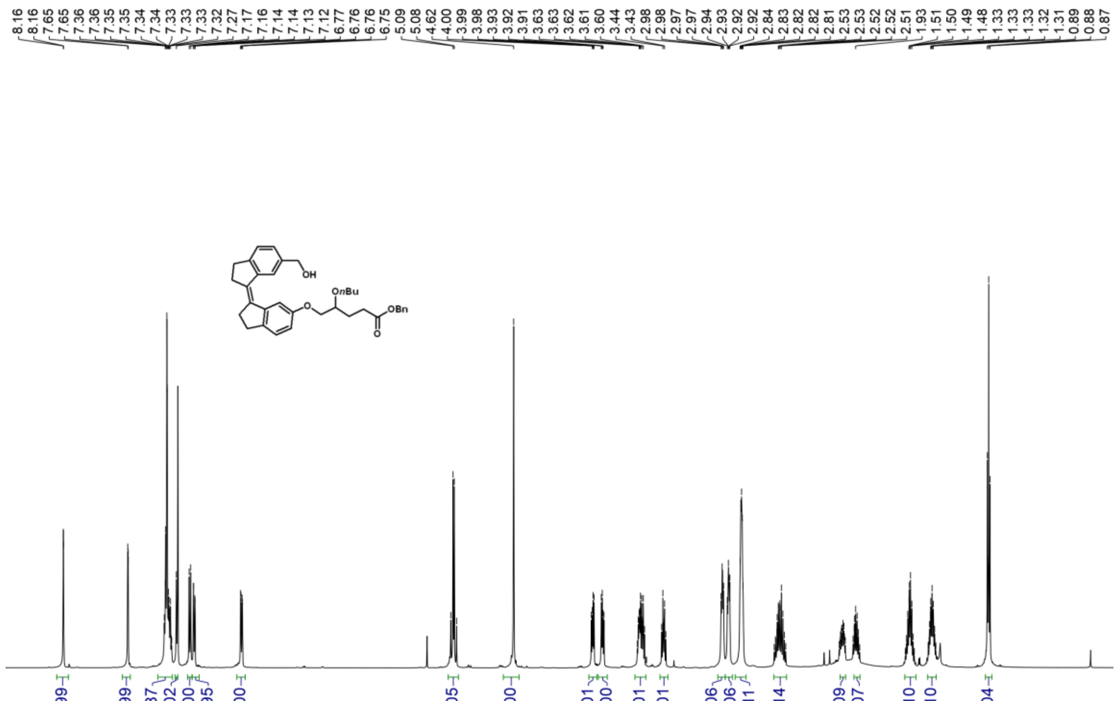

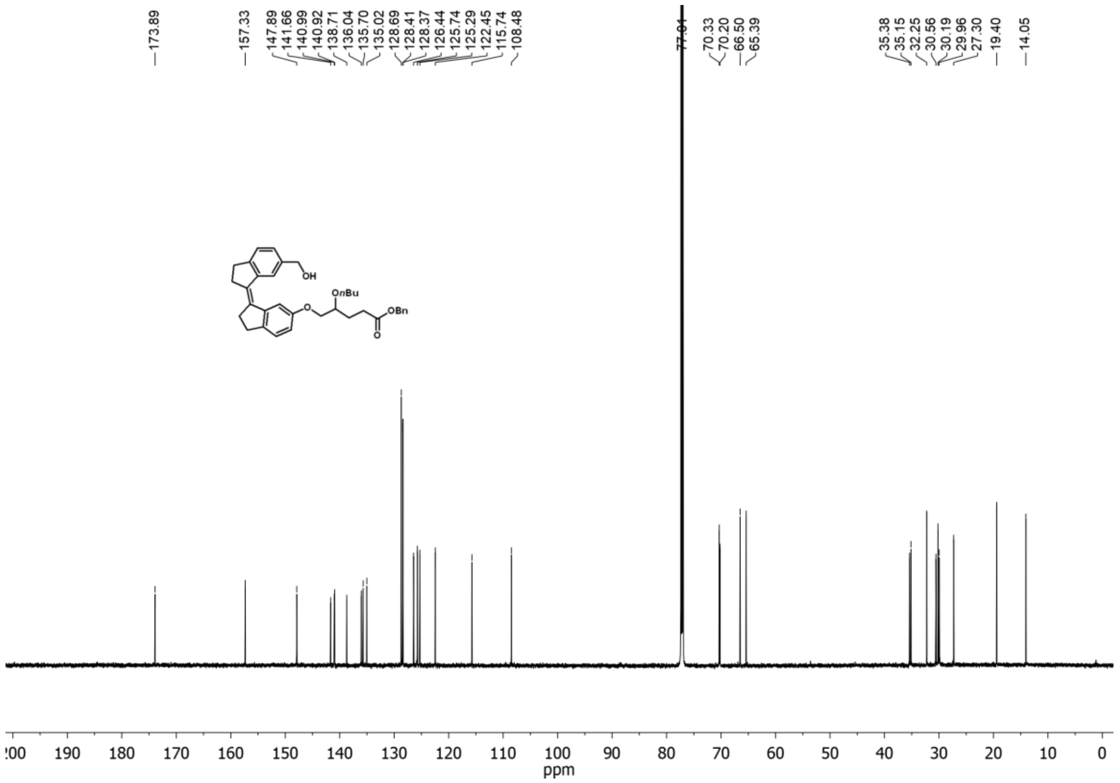

13. References

1. Akbulatov, S.; Tian, Y.; Kapustin, E.; Boulatov, R. Model Studies of the Kinetics of Ester Hydrolysis under Stretching Force. *Angew. Chem., Int. Ed.* **2013**, *52*, 6992−6995.
2. Bhagat, M. N.; Bennett, C. K.; Chang, G.-F.; Zhu, Y.; Raghuraman, A.; Belowich, M. E.; Nguyen, S. T.; Broadbelt, L. J.; Notestein, J. M. Enhancing the Regioselectivity of $B(C_6F_5)_3$-Catalyzed Epoxide Alcoholysis Reactions Using Hydrogen-Bond Acceptors. *ACS Catal.* **2019**, *9* (10), 9663−9670.
3. Dolomanov, O.V.; Bourhis, L. J.; Gildea, R. J.; Howard, J. A. K.; Puschmann, H. *J. Appl. Cryst*. **2009**, *42*, 339−341.
4. Sheldrick, G.M. *Acta Cryst*. **2015**, *A71*, 3−8.
5. Sheldrick, G.M. *Acta Cryst*. **2015**, *C71*, 3−8.
6. Bruker, *SAINT*, *V8.40B*, Bruker AXS Inc., Madison, Wisconsin, USA.
7. L. Krause, R. Herbst-Irmer, G. M. Sheldrick, D. Stalke, *J. Appl. Cryst.* **2015**, *48*, 3–10.
8. C. R. Groom, I. J. Bruno, M. P. Lightfoot, S. C. Ward, *Acta Cryst.* **2016**, *B72*, 171–179.
9. D. Kratzert, *FinalCif*, *V125*, https://dkratzert.de/finalcif.html.
10. Frisch, M. J., Trucks, G. W., Schlegel, H. B., Scuseria, G. E., Robb, M. A., Cheeseman, J. R., Scalmani, G., Barone, V., Mennucci, B., Petersson, G. A., Nakatsuji, H., Caricato, M., Li, X., Hratchian, H. P., Izmaylov, A. F., Bloino, J., Zheng, G., Sonnenberg, J. L., Hada, M., Ehara, M., Toyota, K., Fukuda, R., Hasegawa, J., Ishida, M., Nakajima, T., Honda, Y., Kitao, O., Nakai, H., Vreven, T., Montgomery, J. A., Jr., Peralta, J. E., Ogliaro, F., Bearpark, M., Heyd, J. J., Brothers, E., Kudin, K. N., Staroverov, V. N., Kobayashi, R., Normand, J., Raghavachari, K., Rendell, A., Burant, J. C., Iyengar, S. S., Tomasi, J., Cossi, M., Rega, N.,Millam, J. M., Klene, M., Knox, J. E., Cross, J. B., Bakken, V., Adamo, C., Jaramillo, J.,

Gomperts, R., Stratmann, R. E., Yazyev, O., Austin, A. J., Cammi, R., Pomelli, C., Ochterski, J. W., Martin, R. L., Morokuma, K., Zakrzewski, V. G., Voth, G. A., Salvador, P., Dannenberg, J. J., Dapprich, S., Daniels, A. D., Farkas, O., Foresman, J. B., Ortiz, J. V., Cioslowski, J., and Fox, D. J.; Gaussian 09, revision D.01; Gaussian Inc.: Wallingford, CT, 2013.

11. Hehre, W. J., Ditchfield, R., and Pople, J. A., *J. Chem. Phys.* **1972**, *56*, 2257-2261.
12. Zhao, Y., and Truhlar, D. G., *Theor. Chem. Acc.* **2008**, *120*, 215-241.
13. Yuanying Li; Fengyi Liu; Bin Wang; Qingqing Su; Wenliang Wang; Keiji Morokuma, *J. Chem. Phys.* **2016**, *145*, 244311.
